# Stabilization of the PP2A-B56**α** complex overcomes venetoclax-azacitidine resistance by impairing OXPHOS in AML

**DOI:** 10.1172/jci.insight.205218

**Published:** 2026-09-15

**Authors:** Silvia Romero-Murillo, Irene Peris, Anna Maria Lucianò, Nerea Marcotegui, Carmen Vicente, Brian Tran, Kelsey Barrie, Caitlin M. O’Connor, Andrea Torres-López, Maria C. Mateos, Maria L. Cayuela, Victoriano Mulero, Joaquín Fernández-Irigoyen, Enrique Santamaría, Maria D. Odero, Goutham Narla

**Affiliations:** 1 Clinical Neuroproteomics Unit, Navarrabiomed, Hospital Universitario de Navarra (HUN), UPNA, Navarra, Spain.; 2 Instituto de Investigación Sanitaria de Navarra, Pamplona, Spain.; 3 Centro de Investigación Médica Aplicada and; 4 Department of Biochemistry and Genetics, University of Navarra, Pamplona, Spain.; 5 Department of Internal Medicine, Division of Genetic Medicine, The University of Michigan Medical School, Ann Arbor, Michigan, USA.; 6 Roger Cancer Center, The University of Michigan Health, Ann Arbor, Michigan, USA.; 7 Departamento de Biología Celular e Histología, Facultad de Biología, Universidad de Murcia, Murcia, Spain.; 8 Instituto Murciano de Investigación Biosanitaria (IMIB) Pascual Parrilla, Murcia, Spain.; 9 Centro de Investigación Biomédica en Red de Enfermedades Raras (CIBERER), Instituto de Salud Carlos III, Madrid, Spain.; 10 Department of Pharmacology, Rogel Cancer Center, University of Michigan, Ann Arbor, Michigan, USA.; 11 Hematology Service, HUN, Pamplona, Spain.; 12 Cancer and Aging Group, Hospital Universitario Virgen de la Arrixaca, and IMIB, Murcia, Spain.; 13 Department of Cancer Biology, The University of Michigan, Ann Arbor, Michigan, USA.

**Keywords:** Cell biology, Hematology, Drug therapy, Phosphoprotein phosphatases, Tumor suppressors

## Abstract

Cell metabolic rewiring is associated with resistance to venetoclax-azacitidine (Ven-Aza) combination therapy and relapse in patients with acute myeloid leukemia (AML). Drug-resistant cells exhibit an enhanced reliance on oxidative phosphorylation (OXPHOS) for energy production. Therefore, impairing mitochondrial metabolism represents an exciting strategy to face this unmet clinical need. We recently demonstrated that the specific activation of the phosphatase PP2A-B56α enhances the proapoptotic efficacy of venetoclax in AML. Here, through leveraging unbiased multi-omics–based approaches and using both genetic and pharmacological tools, we define key roles for the tumor suppressor PP2A-B56α complex in OXPHOS regulation and treatment response in disease-relevant AML models. From a translational perspective, the specific stabilization of PP2A-B56α heterocomplex with the potentially novel PP2A molecular glue activator, RPT04402, reduces OXPHOS levels in treatment-resistant AML cells and improves treatment response in both Ven-Aza–sensitive and –resistant AML cell lines, primary cells, and in vivo models. Together, our work supports further research on targeted combination therapy approaches based on PP2A-B56α stabilization to counteract OXPHOS-related treatment resistance and improve AML responses in a patient population with historically poor outcomes.

## Abstract 2

A new PP2A-B56α-based strategy overcomes treatment resistance in preclinical AML models by targeting cell metabolism, offering a promising approach for future AML therapy

## Graphical abstract

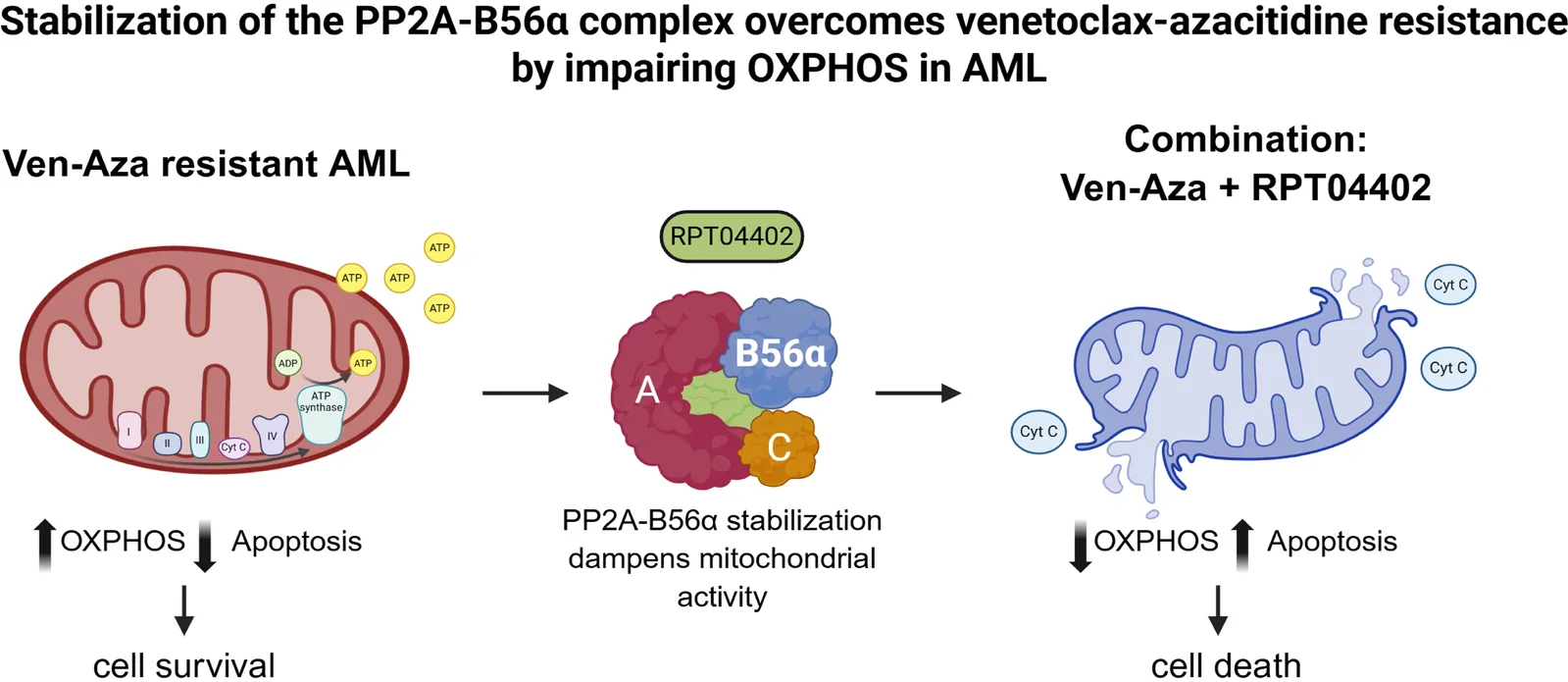

## Introduction

Despite advances in targeted treatments for patients with acute myeloid leukemia (AML), 5-year survival rates remain extremely low, being 11.2% for patients ≥ 65 years old (1). Moreover, patients unfit for intensive treatment regimens exhibit a median overall survival (OS) of 5–10 months, highlighting the need for novel AML treatments (2). A practice-changing milestone for the treatment of this cohort of patients occurred with the approval of the venetoclax (BCL2 inhibitor) and azacitidine (hypomethylating agent) combination therapy (Ven-Aza). However, the efficacy of this combination therapy is limited by highly aggressive refractory disease (OS, 2.4 months) and frequent relapse (OS, 18 months), limiting these patients’ OS (3). Several genetic and nongenetic mechanisms driving Ven-Aza resistance have already been described, including the acquisition of oncogenic signaling mutations (4, 5); inactivation of *TP53* or apoptosis effector genes (5); upregulation of anti-apoptotic proteins (4); cell energy metabolism alterations (6–11); and AML cellular differentiation (12). Nevertheless, a better characterization of these resistance mechanisms is crucial to develop novel therapeutic strategies to improve patients with AML outcomes (13).

Dysregulated kinase–phosphatase balance drives cancer progression and treatment resistance, making the interplay between these enzymes a critical target for overcoming therapeutic resistance (14). To this end, our group and others reported recurrent inactivation of the tumor suppressor protein phosphatase 2A (PP2A) in AML through distinct nongenetic mechanisms (15). Importantly, we recently demonstrated that the pharmacological activation of PP2A enhances venetoclax-based therapies in primary samples at diagnosis and preclinical models of AML (16). PP2A is a family of heterotrimeric serine/threonine phosphatases that regulates a wide range of cellular processes. It comprises a catalytic C-subunit, a scaffold A-subunit, and a substrate-directing regulatory B-subunit. Of the 4 distinct families of B subunit (B55, B56, PR72/130, and STRN), B55 and B56 families are more associated with tumor suppressor roles (17). Given that the PP2A-B56α heterotrimer regulates an extensive range of signal transduction pathways involved in the development and progression of AML, and its specific activation synergizes with venetoclax-based treatments (16), we hypothesized that the PP2A-B56α complex might play a role in the response to Ven-Aza and that its pharmacological stabilization could provide a novel therapeutic approach for Ven-Aza–resistant disease.

Here, we show that the B56α regulatory subunit participates in the response to the clinically approved Ven-Aza therapy. By leveraging unbiased multi-omics–based approaches, combining transcriptomic and proteomic analyses, and using both genetic and pharmacological tools to validate our observations, we demonstrate that the B56α subunit specifically regulates oxidative phosphorylation (OXPHOS), identifying, to our knowledge, a previously unrecognized role of the PP2A-B56α complex in regulating cell metabolism in AML, one of the main mechanisms driving treatment resistance in this disease (8). Furthermore, this key function of B56α can be pharmacologically targeted with RPT04402, a promising PP2A molecular glue activator (PMG), in de novo and treatment-resistant AML. Importantly, the addition of RPT04402 to Ven-Aza treatment significantly disrupts mitochondrial respiration and results in further enhancement of its antileukemic activity in both Ven-Aza–sensitive and –resistant AML cell lines, primary cells, and in vivo models. Altogether, we present a promising targeted therapy approach consisting of PP2A-B56α stabilization to overcome OXPHOS-dependent resistance in AML.

## Results

### Reduced B56α expression impairs Ven-Aza treatment response.

Our group previously reported that *PPP2R5A* mRNA levels (encoding B56α protein) might act as a potential predictive biomarker for treatment response to venetoclax-based therapies combined with PP2A pharmacological activation in AML (16). These results led us to wonder whether B56α subunit expression could affect treatment response to the clinically approved Ven-Aza combination therapy. To evaluate this hypothesis, we first explored the OS of a subset of patients from the OHSU BeatAML2 cohort treated with the Ven-Aza regimen, for whom *PPP2R5A* expression data were available (*n* = 15) (18). Based on *PPP2R5A* z-score median values, these patients were grouped into groups expressing low *PPP2R5A* (*n* = 8, normalized [norm.] expression = 5.99–6.90) and high *PPP2R5A* (*n* = 7, norm. expressio*n* = 6.91–7.25). Interestingly, patients with AML with reduced *PPP2R5A* expression demonstrated a significantly shorter median OS as compared with patients with high *PPP2R5A* expression levels (9.15 versus 17.13 months, respectively; *P* = 0.0129) (Figure 1A). Conversely, low mRNA expression of the other tumor suppressor PP2A regulatory subunits from the B56 family or B55α (*PPP2R2A*) did not show reduced OS (Supplemental Figure 1; supplemental material available online with this article; https://doi.org/10.1172/jci.insight.205218DS1). Next, *PPP2R5A* expression and synergy scores for Ven-Aza treatment were calculated in a series of 11 AML primary samples (Supplemental Table 1). A positive correlation was observed (*r* = 0.62, *P* = 0.044); samples with lower *PPP2R5A* expression showed lower synergistic scores upon Ven-Aza treatment (Figure 1B), suggesting that B56α might play a role in regulating combination therapy response in patients with AML.

To determine if reduced PP2A-B56α expression drives combination therapy resistance, B56α^–/–^sublines were generated by CRISPR-Cas9 in HL-60, OCI-AML3, and MV4-11 AML cell lines (Figure 1C and Supplemental Figure 2, A and B). Loss of B56α subunit expression did not generally affect baseline cell growth or GI50 values upon single venetoclax and azacitidine treatments (Supplemental Figure 2, C and D). Interestingly, the Ven-Aza combination did not show synergy in B56α^–/–^ cells as compared with B56α^WT^ cells in all 3 lines tested (Figure 1, D and E, and Supplemental Figure 2, E and F). Remarkably, the reconstitution of B56α protein expression in HL-60 B56α^–/–^ g1 clone recovered Ven-Aza treatment sensitivity (Figure 1, F–H, and Supplemental Figure 3, A and B). Indeed, Ven-Aza treatment was significantly less effective in inducing apoptosis in the HL-60 B56α^–/–^ clones (approximately 20% of cell death in B56α^–/–^ clones versus 38.9% in B56α^WT^ cells). This effect was reversed upon B56α reconstitution (Figure 1I). Furthermore, the loss of mitochondrial membrane potential (MMP) after Ven-Aza treatment was also significantly reversed in HL-60 B56α^–/–^ cells (approximately 30% of cells with loss of MMP in B56α^–/–^ versus 51.7% in B56α^WT^ cells) and recovered up to 49.6% of TMRM^–^ cells after B56α reconstitution (Figure 1J). Cell death and MMP were also measured in OCI-AML3 and MV4-11 B56α^–/–^ CRISPR sublines upon Ven-Aza regimen, showing similar results (Supplemental Figure 3, C and D). Importantly, B56α^–/–^ cells did not present an intrinsic resistance to apoptosis (Supplemental Figure 3E).

To further corroborate whether the observed effects were B56α specific and not a result of general tumor suppressive activity of PP2A, we generated HL-60 sublines with genetic inactivation of B55α regulatory subunit, where no differences in Ven-Aza treatment response, apoptosis induction, or loss of MMP were noted between B55α^WT^ and B55α^–/–^ cells (Supplemental Figure 4).

Collectively, these results highlight the relevance of the B56α regulatory subunit in modulating the response to Ven-Aza combination therapy in AML and suggest that a resistance mechanism to this drug combination could be regulated by PP2A-B56α signaling.

### Transcriptomic and proteomic analyses indicate that OXPHOS is upregulated in HL-60 B56α^–/–^ cells.

To better define the pathways regulated by PP2A-B56α in AML cells, we compared the transcriptomic profile of the HL-60 B56α^WT^ and B56α^–/–^ clones using RNA-seq (Figure 2A and Supplemental Table 2). Gene set enrichment analysis (GSEA) was performed using the hallmark gene set collection (19). HL-60 B56α^–/–^ cells presented “Oxidative_phosphorylation” and “MYC_targets_V1” hallmarks positively regulated, and 13 other gene sets downregulated, such as “Apoptosis” and several immune response-related pathways (*P* < 0.05) (Figure 2, B and C, and Supplemental Table 3). Notably, transcriptomic AML patient data from the low *PPP2R5A* expression group (OHSU BeatAML2 database; ref. 18) showed that “Oxidative_phosphorylation” and “MYC_targets_V1” hallmarks were also upregulated, with the “TNFA_signaling_via_NFKB” pathway being among the most downregulated in both analyses (Figure 2, B and C; Supplemental Figure 5, A–C; and Supplemental Tables 4–7). Eighty-four genes from the OXPHOS hallmark gene set (48.6% of genes) were commonly deregulated in HL-60 B56α^–/–^ clones and patients with low *PPP2R5A* expression (Supplemental Figure 5D and Supplemental Table 8). These data point out similar phenotypes between our HL-60 clones lacking the B56α subunit and patients with AML with low *PPP2R5A* expression levels. Some of these pathways, such as MYC and NF-κB, have already been described to be regulated by PP2A-B56α (20, 21), validating our experimental approach. To our knowledge, no previous studies have identified a role for B56α in OXPHOS in AML.

To better define the biologically significant pathways regulated by the PP2A-B56α complex in AML, proteomic analyses were also performed in our CRISPR-Cas9 HL-60 B56α sublines (Figure 2D and Supplemental Table 9). Expression of 16% (638 proteins) of the total proteins detected was significantly altered (*P* < 0.05) in HL-60 B56α^–/–^ sublines compared with HL-60 B56α^WT^ cells (Supplemental Table 9). Pathway analysis of the proteomic data was conducted using Hallmark Gene Sets enrichment analysis of the Metascape database (22). Twenty-seven pathways were significantly deregulated at proteomic level (*P* < 0.01), including those which PP2A has been shown to regulate, including E2F and MYC targets, apoptosis, p53, and PI3K/AKT/mTOR (17). Interestingly, these results also reveal important differences in cell metabolism, encompassing adipogenesis, glycolysis, fatty acid metabolism, and OXPHOS, which was the most significantly altered pathway in B56α-deficient cells (*P* < 0.0001) (Figure 2E and Supplemental Table 10). Within OXPHOS pathway, upregulated proteins were mostly mitochondrial proteins related to complex I of the electron-transporting chain (ETC) (NDUF family proteins) (Figure 2F and Supplemental Table 10). Moreover, among the differentially expressed proteins detected in HL-60 B56α^–/–^ cells, 38% were also deregulated transcriptionally, with OXPHOS pathway components significantly upregulated at both the transcriptional and proteomic levels (Supplemental Figure 5, E–G). Measurement of OCR confirmed the activation of the OXPHOS pathway in HL-60 B56α^–/–^ cells when compared with HL-60 B56α^WT^ and B56α-reconstituted cells in terms of basal and maximal mitochondrial respiration rates and spare respiratory capacity (SRC) (Figure 2G). Moreover, HL-60 B56α^–/–^ clones showed higher mitochondrial complex I activity as compared with the B56α^WT^ and B56α-reconstituted clones (Figure 2H). Genetic inactivation of B56α regulatory subunit consistently increased basal and maximal mitochondrial respiration across different B56^–/–^AML sublines, indicating a conserved enhancement of mitochondrial respiratory capacity (Supplemental Figure 6, A–D). However, the downstream bioenergetic adaptations differed between cell lines: HL-60 and MV4-11 B56α^–/–^ cells enhanced complex I–dependent respiration, whereas OCI-AML3 maintained increased mitochondrial respiration through Complex I–independent electron entry into the ETC, pointing to alternative metabolic pathway engagement (Supplemental Figure 6, A–D). Only MV4-11 translated the increased respiratory activity into higher ATP production and SRC, suggesting improved coupling efficiency. HL-60 instead showed an enhanced SRC (Figure 2G), consistent with expanded respiratory reserve rather than ATP output. No differences were found in the OCR between HL-60 B55α^–/–^ and B55α^WT^ cells (Supplemental Figure 6E).

Collectively, these observations reveal a key role for the PP2A-B56α holoenzyme in regulating OXPHOS in AML.

### Ven-Aza–resistant AML models demonstrate upregulated OXPHOS activity.

Ven-Aza treatment disrupts OXPHOS in de novo AML blasts (23, 24). However, drug-resistant leukemia cells rewire their metabolism to bypass this inhibition and still rely on mitochondrial respiration for survival, upregulating OXPHOS to acquire resistance to Ven-Aza treatment (7–9, 12, 25). To further explore the mechanisms of treatment resistance, we generated Ven-Aza–resistant AML cell models. HL-60, OCI-AML3, and MV4-11 cell lines were exposed to increasing doses of both drugs until they developed resistance to single and combination therapy (HL-60-R, OCI-AML3-R, and MV4-11-R) (Figure 3, A and B, and Supplemental Figure 7, A and B). Resistant cell lines showed no induction of apoptosis or loss of MMP after treatment with over 8 times higher doses than the parental cell lines (Supplemental Figure 7, C and D). In a s.c. mouse model, HL-60-R cells were completely resistant to Ven-Aza therapy as compared with the parental cells (*P* < 0.0001), reproducing in vitro results (Figure 3C). Proteomic analyses of the HL-60-R compared with HL-60 parental cells revealed altered metabolic processes in HL-60-R cells, with OXPHOS being the most deregulated pathway, followed by fatty acid metabolism and adipogenesis (*P* < 0.0001) (Figure 3D, Supplemental Figure 8, and Supplemental Tables 11–13). Seahorse assays confirmed the significantly augmented OXPHOS activity in Ven-Aza–resistant cells. Although the resistant metabolic phenotype across AML cell lines was not identical, all resistant models exhibited increased maximal respiratory capacity and complex I activity (Figure 3, E–G), indicating that enhanced mitochondrial respiratory potential is a common adaptation to Ven-Aza resistance in AML. Importantly, it strengthens our observations on the connection between OXPHOS upregulation and Ven-Aza resistance in patients with AML with low *PPP2R5A* expression and in cell lines harboring loss of B56α.

### PP2A-B56α heterocomplex stabilization decreases OXPHOS in Ven-Aza AML-resistant models.

Given the role of B56α in OXPHOS regulation and the importance of mitochondrial metabolism in driving AML treatment resistance, we tested whether RPT04402, a potentially novel PMG that specifically stabilizes active PP2A-B56α complexes (26), was able to reverse the OXPHOS-related Ven-Aza–resistant phenotype. RPT04402 treatment caused a broad-spectrum effect on mitochondrial respiration in our 3 AML parental cell lines, as evidenced by dose-dependent decreases in the levels of basal and maximal respiration and ATP production (Figure 4A and Supplemental Figure 9, A and B). These effects were significantly attenuated in B56α^–/–^ clones, especially the maximal respiration levels, highlighting the specificity of the compound to PP2A-B56α complexes and defining a key role for the PP2A-B56α heterotrimer in regulating mitochondrial maximal respiration (Figure 4B and Supplemental Figure 9, C and D). To further corroborate the specificity of RPT04402, we ectopically expressed a V5-tagged Aα protein in HL-60 cells (Supplemental Figure 10A). Then, Aα-V5 interactions with the catalytic subunit and the 5 members of the B56 regulatory family (α, β, γ, δ, and ε) were analyzed using a Proximity Ligation Assay (PLA). PMG treatment resulted in a significant increase in only PP2A-B56α complexes, enhancing B56α heterotrimerization over the rest of the B56 subunits and confirming the high specificity of RPT04402 to this heterotrimer (Figure 4, C and D, and Supplemental Figure 10, B and C). Western blots of all these subunits were performed to verify that the increase in Aα-V5/B56α binding was not a result of expression variations upon drug treatment (Supplemental Figure 10, D and E). Additionally, RPT04402 treatment of the OCI-AML3 and MV4-11 B56α^WT^ sublines drove a broad-spectrum decrease in OCR, effects that were generally reversed when B56α was knocked down (Supplemental Figure 11).

B56α subunit expression was verified at mRNA and protein levels in our resistant models before testing the effects of RPT04402 treatment (Supplemental Figure 12, A–C). The Ven-Aza–resistant phenotype did not affect RPT04402 sensitivity in these cells (Supplemental Figure 12D). Importantly, OCR was significantly reduced in resistant models upon RPT04402 treatment, similarly to the parental baseline levels (Figure 4, E–G). Collectively, these results demonstrate that the stabilization of PP2A-B56α complex specifically decreases OXPHOS-dependent energy production in both Ven-Aza–sensitive and –resistant AML models.

### Combined treatment with RPT04402, venetoclax, and azacitidine disrupts OXPHOS and overcomes Ven-Aza resistance in AML.

Previous results from our group demonstrated that PP2A activators enhance responses to venetoclax-based combinations (16). Therefore, we evaluated the efficacy of the triple combination Ven-Aza–RPT04402 in our AML models. RPT04402 synergistically enhanced the antileukemic effect of the standard-of-care Ven-Aza combination in both parental and treatment-resistant AML cells (Figure 5A and Supplemental Figure 13A). Indeed, only the triple combination therapy markedly reduced basal and maximal respiration and ATP production, disrupting mitochondrial respiration in Ven-Aza–resistant models (Figure 5, B–D). In the parental cell lines (Ven-Aza–sensitive cells), both Ven-Aza and triple treatments decreased OXPHOS (Supplemental Figure 13B). Since these viability assays were performed using an MTS activity assay, which measures changes in metabolic activity, these cell viability changes were also confirmed using an independent DNA-based viability assay (Supplemental Figure 13, C and D). The efficacy of Ven-Aza–RPT04402 therapy relied specifically on the B56α PP2A regulatory subunit, since synergy score values were not significantly increased by the triple therapy in HL-60 B56α^–/–^ clones as observed in the HL-60 B56α^WT^ and in HL-60 B55α CRISPR-Cas9 sublines (Supplemental Figure 14).

Although RPT04402 alone did not significantly induce apoptosis or mitochondrial depolarization at tested doses, its addition to the Ven-Aza therapy significantly enhanced both apoptotic cell death and mitochondrial membrane depolarization in parental and resistant AML cells (Supplemental Figure 15). This observation demonstrates that the observed metabolic alterations are accompanied by functional activation of mitochondrial apoptosis, thereby reinforcing the therapeutic potential of the triple combination and contributing to overcome resistance to the Ven-Aza regimen.

After checking that RPT04402 alone or combined with venetoclax and azacitidine did not present a significant reduction in the number or type of normal human CD34^+^ hematopoietic stem/progenitor colonies (Supplemental Figure 16, A–C), we next tested the triple combination in 3 AML patient–derived primary cells. Samples AML-1 and AML-2 were obtained at diagnosis, while sample AML-3-relapsed AML-3-R was obtained at relapse after 6 cycles of Ven-Aza treatment (Supplemental Figure 16D and Supplemental Table 1). Interestingly, the AML-3-R sample was enriched in CD34^+^CD38^–^CD45RA^+^ cells (Figure 5, E and F, and Supplemental Figure 16E), an immunophenotype related to leukemia stem cells (LSCs) (8, 27, 28). In both the newly diagnosed and relapsed samples (Ven-Aza–sensitive or –resistant) carrying either ELN-favorable or adverse-risk mutations (Supplemental Table 1), the addition of RPT04402 doubled the synergy score of Ven-Aza therapy (Figure 5G and Supplemental Figure 16F).

Collectively, the stabilization of the PP2A-B56α complex by RPT04402 combined with Ven-Aza treatment counteracts dysregulated OXPHOS signaling, one of the key mechanisms driving treatment resistance, and improves the efficacy of Ven-Aza treatment in de novo and relapsed AML samples.

### RPT04402 improves the response to Ven-Aza in treatment-resistant in vivo models.

To evaluate the triple treatment efficacy in vivo, we took advantage of zebrafish xenograft models, as they allow noninvasive imaging of tumor growth, high experimental throughput, and short-term results; they also conserve similar hematopoietic regulation to humans (29–31). HL-60-R and OCI-AML3-R cells were xenotransplanted into zebrafish larvae orthotopically, and RPT04402, Ven-Aza, and the triple combination were added to water for 72 hours (Figure 6A). Consistent with our results described above, Ven-Aza treatment was not effective in reducing HL-60-R or OCI-AML3-R cell infiltration (*P* = 0.06 and *P* = 0.35, respectively) (Figure 6, B–E). Remarkably, RPT04402 single-agent treatment and RPT04402 in combination with Ven-Aza significantly reduced cell infiltration in both Ven-Aza–resistant cells (RPT04402: 36.6% and 41.6%; triple combo: 66.8% and 53.2%; in HL-60-R and OCI-AML3-R, respectively) and Ven-Aza–sensitive cells (RPT04402: 27.3% and 44%; triple combo: 57.8% and 75.7%; in HL-60 and OCI-AML3, respectively) (Figure 6, B–E). S.c. mouse xenograft experiments in Ven-Aza–resistant cell line models (Figure 6F) corroborated the in vivo activity of RPT04402 (23% and 15% of tumor growth inhibition [TGI] in HL-60-R and OCI-AML3-R, respectively). Combination treatment of RPT04402 with Ven-Aza improved the efficacy of the therapy in these fast-growing Ven-Aza–resistant tumors (29% TGI in HL-60-R and 27.3% in OCI-AML3-R) without any sign of toxicity (Figure 6, G and H).

Finally, we generated a zebrafish patient-derived xenograft (PDX) using the AML-3-R sample at relapse after Ven-Aza treatment, following the same workflow as previously described (Figure 6A). RPT04402 treatment alone effectively reduced cell infiltration (30.8% of cell infiltration inhibition) and synergized with Ven-Aza therapy (62.9% of cell infiltration inhibition) in this AML relapsed model in vivo (Figure 6I).

Altogether, RPT04402 shows in vivo activity in both zebrafish and mice, and Ven-Aza–RPT04402 combination is effective on distinct Ven-Aza–resistant AML models, setting the basis for a promising therapeutic approach to overcome treatment resistance in AML.

## Discussion

Primary resistance to Ven-Aza treatment represents one-third of treated patients, and 52% of those who initially respond subsequently relapse (3), limiting the therapeutic options for patients with AML unfit for intensive chemotherapy. Research efforts to identify genetic and nongenetic factors of response/resistance to this combined therapy have resulted in the development of novel combination strategies (32–34). On this matter, we recently published a study highlighting a role for the PP2A-B56α complex in driving the synergistic proapoptotic activity observed upon PP2A activation and venetoclax treatment in AML preclinical models (16), which prompted us to test whether PP2A-B56α might also affect treatment response to the Ven-Aza combination.

Here, we found that patients treated with the Ven-Aza regimen and reduced *PPP2R5A* expression presented significantly shorter OS than patients with high *PPP2R5A* expression. Moreover, we confirmed in a panel of AML primary samples that cells with lower *PPP2R5A* expression levels showed a lower synergistic effect score upon Ven-Aza treatment. These results suggest that *PPP2R5A* expression levels could be a valuable clinical biomarker. Genetic inactivation of B56α regulatory subunit in AML cell lines and its following restoration confirmed the specific role of B56α in modulating the response to Ven-Aza treatment in AML, identifying a resistance mechanism resulting from PP2A-B56α downstream signaling deregulation.

Unbiased interrogation of the transcriptomic and proteomic profile of our CRISPR-Cas9 B56α AML cell lines allowed us to identify a central role of PP2A-B56α in regulating mitochondrial metabolism. Additionally, several multi-omics studies have linked PP2A with cell energy regulation and lipid metabolism, suggesting that other PP2A subunits may play a role in cell metabolism. For instance, metformin treatment inhibits CIP2A, a PP2A endogenous inhibitor, and triggers the upregulation of B56δ, impairing the metabolic plasticity and tumor growth in melanoma models (35). Moreover, consistent with our results, a study on brain cancer showed that PP2A reactivation overcomes mitochondrial apoptosis resistance through PP2A-elicited shutdown of compensatory OXPHOS (36).

In vitro changes in OCR and complex I activity in our AML models validated the omics analyses, where proteins from the NDUF and MT-ND families were significantly upregulated in B56α^–/–^ sublines, corroborating the function of this subunit in OXPHOS regulation. Several studies showed that complex I is overexpressed in various tumor types, including AML (37), potentially explaining the resistance to Ven-Aza treatment observed in B56α^–/–^ cells. Indeed, complex I inhibitors have been tested in AML clinical trials, but they demonstrated limited antitumor activity at tolerated doses (38, 39). Interestingly, within our transcriptomic/proteomic results, mitochondrial ribosomal proteins were also upregulated, highlighting a potential role for the PP2A-B56α complex in regulating OXPHOS function by modulating the translation of mitochondrial proteins. However, the specific role of the PP2A-B56α heterotrimer in directing mitochondrial structure, biogenesis, or regulating alternative metabolic pathways that stimulate the ETC is still unknown. Our findings indicate that B56α genetic inactivation promotes mitochondrial respiratory remodeling, while the mechanisms supporting the enhanced respiratory phenotype after its inactivation are influenced by the metabolic background of each AML cell line. Further studies are needed to elucidate the mechanism by which B56α specifically regulates these metabolic processes.

To better understand the mechanisms of resistance to Ven-Aza therapy, we generated Ven-Aza–resistant cell lines that displayed cell line–dependent increased mitochondrial bioenergetic capacity, consistent with the well-recognized metabolic flexibility of treatment-resistant AML cells. Similar to our resistant models, it was reported that drug-resistant LSCs rewire their metabolism to avoid therapy-driven OXPHOS disruption by using other metabolic substrates, such as fatty acids (7), mitochondrial calcium (40), or increasing nicotinamide catabolism (9). Thus, disturbing the metabolic vulnerabilities of drug-resistant leukemia cells that directly or indirectly target OXPHOS represents an appealing therapeutic strategy to counteract drug resistance and relapse in AML (8, 9, 40–42).

Therapeutically, we have studied the effect of RPT04402 in our AML-resistant models, a first-in-class PMG that promotes the formation of the Aα/B56α/C heterotrimer. The stabilization of PP2A-B56α complexes with RPT04402 combined with Ven-Aza markedly disrupts OXPHOS in our resistant cells and synergistically improves responses to standard treatment. We confirmed the synergistic effect between the 3 drugs in 2 complementary in vivo models used as a proof of concept in vivo platform to assess the antileukemic activity of the triple combination. Although s.c. mouse xenografts are commonly used in leukemia research (43–45), they lack the bone marrow microenvironment and do not reproduce the systemic dissemination that critically influences AML biology and therapy response, which may account for the modest TGI observed. Validation in i.v. and PDX models will be required to confirm these findings in a clinically relevant leukemia context. In contrast, the zebrafish model provides an orthotopic context in which AML cells colonize the caudal hematopoietic tissue, the equivalent of human bone marrow, enabling rapid, high-throughput, and statistically robust evaluation of drug treatment response. Importantly, results from the relapsed zebrafish PDX, enriched in CD34^+^CD38^–^CD45RA^+^ LSCs (8), confirm the antileukemic effects of the triple combination, supporting the benefit of this therapeutic approach in Ven-Aza–resistant AML based on LSC population eradication, a long-sought goal in AML research (46). However, secondary bone marrow transplantation studies would be required to directly determine whether the treatment eliminates LSC or reduces relapse potential. In addition, the triple combination of Ven-Aza–RPT04402 also synergized in 2 additional AML primary samples from patients obtained at diagnosis carrying ELN-favorable and adverse-risk mutations, indicating that the triple therapy might be advantageous among patients with AML with diverse mutational/cytogenetic risk features.

Clinically, Ven-Aza resistance and relapse remain a major barrier in AML, with few effective salvage strategies once resistance emerges. Conceptually, our data demonstrate that PP2A-B56α reactivation is not merely additive with Ven-Aza but restores sensitivity in genotypically and clinically distinct resistant contexts, supporting further investigation and potential clinical translation of this triple therapy approach. Despite the broad tumor suppressor activity that PP2A has demonstrated in multiple preclinical studies (47), to date, no PP2A glue has been approved for use in patients. Nevertheless, preclinical characterization of RPT04402 is underway to support an IND filing, which will form the potential basis for a Phase I/IB clinical trial in uterine cancer. Therefore, the design of therapy approaches — including selectively directing PP2A holoenzyme composition and, thus, substrate specificity — has now, more than ever, an enormous clinical potential (47, 48).

## Methods

### Sex as a biological variable.

Our study examined primary and bioinformatic samples from male and female patients with AML. Sex-stratification of these data for analysis was not feasible due to the relatively small sample size. S.c. xenograft studies exclusively examined female mice, but findings are expected to be relevant for both sexes.

### Human cell lines, primary AML cells, normal bone marrow CD34^+^ cells and compounds.

HL-60, OCI-AML3, and MV4-11 cell lines were obtained from DSMZ. Cell lines were maintained in RPMI-1640 (GIBCO; Thermo Fisher Scientific) culture medium supplemented with 10% FBS (GIBCO; Thermo Fisher Scientific), penicillin G (100 U/mL), and streptomycin (0.1 mg/mL) (GIBCO; Thermo Fisher Scientific), and grown at 37°C in a 5% CO_2_ atmosphere. Cell lines were regularly tested negative for Mycoplasma and authenticated by short-tandem repeat DNA typing. Primary AML cells were purified from 10 patients with AML at diagnosis and 1 at relapse (Supplemental Table 1). AML primary cells were processed, cultured, and characterized by flow cytometry using CD34 (345801), CD38 (555460), and CD45RA (335039) (BD Biosciences); CD123 (Miltenyi Biotec; 130-113-326); and IL-1RAP (R&D systems; FAB6761V) antibodies. Normal human bone marrow–derived CD34^+^ cells were obtained from STEMCELL Technologies. Cells were treated for 12, 24, or 48 hours with venetoclax (Selleckchem; S8048), azacitidine (Selleckchem; S1782), and/or RPT04402 (RAPPTA).

### Gene expression and survival analysis of public datasets.

AML patient data were obtained from the OHSU Beat AML.2 study (18). Patient data were filtered by *Current Regimen* (venetoclax [ABT-199], azacitidine). Low and high PP2A B subunit groups were generated from the median of gene mRNA normalized expression values, and OS was compared between groups.

### Generation of Ven-Aza–resistant cell lines.

Venetoclax- and azacitidine-resistant cells were generated by the addition of increasing concentrations of both drugs to AML cell lines. Treatment started with 50 nM venetoclax and 250 nM azacitidine in HL-60, 500 nM venetoclax, and 750 nM azacitidine in OCI-AML3 as well as 20 nM venetoclax and 1 μM azacitidine in MV4-11. Doses were doubled every 2 weeks for 2 months. Then, drugs were removed from culture medium and resistance was tested.

### Cell viability and drug combination assays.

Cell lines (25,000–35,000 cells/well) or AML primary cells (20,000 cells/well) were plated in 96-well plates, and compounds were added. Growth inhibition was determined at increasing concentrations of venetoclax, azacitidine, and/or RPT04402 using CellTiter96 Aqueous One-Solution Cell Proliferation assay (Promega; G3581) or CyQUANT Direct Cell Proliferation Assay (Thermo Fisher Scientific; C35011) after 48-hour (cell lines) or 60-hour (primary AML cells) treatment. Synergism was calculated using the SynergyFinder Plus webtool and represented with highest single agent (HSA) score (49). A combined treatment was considered synergistic when HSA >10.

### PLA.

After 24 hours of treatment, 400,000 cells per well were plated in 8-well chamber slides (CellTreat, 229168), previously treated with Poly-L-Lysine (Sigma-Aldrich, P4707). Cells were incubated for 30 minutes at room temperature (RT), before being fixed with 4% paraformaldehyde for 10 minutes, washed 3 times with PBS, permeabilized with 0.05% Triton X for 10 minutes, and washed 3 times with PBS. Single-stranded DNA (D9156) was diluted 1:10 in dH_2_O, vortexed, boiled for 10 minutes, and chilled for 10 minutes on ice. The above solution was diluted in Duolink Blocking Solution (DUO82007) at 1:60, vortexed, and added to the fixed and permeabilized cells. Slides were incubated in a humidity chamber for 60 minutes at 37°C. Primary antibodies V5 (Cell Signaling, 13202) and B56α (Dr. Ogris’ Lab) were prepared in Duolink Antibody Diluent (DUO82008) at 1:100 and added. Slides were incubated in a humidity chamber at RT for 2 hours. Overnight incubation was performed when primary antibodies were B56β (Santa Cruz Biotechnology, sc-515676), B56γ (Santa Cruz Biotechnology, sc-374380), B56δ (Abcam, ab188323), B56ε (Abcam, ab198500), Cα/β (Abcam, ab106262), and V5 (Biorad, MCA1360G) prepared at 1:100 in Duolink Antibody Diluent. Afterward, the samples were washed twice in 1 times Wash Buffer A (DUO82046) at RT. Duolink PLA probes (DUO82002 and DUO82004) were diluted 1:5 in Duolink Antibody Diluent and added to samples. Slides were incubated in a humidity chamber for 60 minutes at 37°C. Next, samples were washed twice in 1 times Wash Buffer A at RT. Ligation buffer (DUO82009) was prepared 1:5 in dH_2_O, ligase enzyme (DUO82027) was added at 1:40 dilution, and slides were incubated in a humidity chamber for 30 minutes at 37°C. Subsequently, samples were washed twice in 1 times Wash Buffer A at RT. Amplification buffer (DUO82011) was prepared 1:5 in dH_2_O, polymerase enzyme (DUO82028) was added at 1:80 dilution, and slides were incubated in a humidity chamber for 3 hours at 37°C. Samples were washed twice in 1X Wash Buffer B (DUO82048) at RT and in 0.01X Wash Buffer B for 1 minute. Finally, slides were mounted using Duolink Situ Mounting Medium with DAPI (DUO82040).

Images were taken using the EVOS M5000 microscope (Thermo Fisher Scientific) and analyzed using ImageJ software (NIH). Corrected total cell fluorescence (CTCF) was calculated as: CTCF = integrated density – (area of analysis × mean background fluorescence).

### Oxygen consumption rate.

Oxygen consumption rate (OCR) was determined using the SeahorseXF Cell Mito Stress Test Kit (Agilent Technologies; 103010-100). After 12-hour treatment, culture media were replaced with XF base medium DMEM (Agilent Technologies; 103575-100) supplemented with 10 mM glucose (Thermo Fisher Scientific; A2494001), 1 mM Na-Pyruvate (Lonza; BEBP13-115E), and 2 mM L-glutamine (Thermo Fisher Scientific; 5030081). Then, 1 × 10^5^ cells were seeded in 25 μg/mL Cell-Tak (Corning; 10317081) precoated wells, centrifuged for 5 minutes at 500 × *g* at RT, and incubated for 30 minutes at 37°C without CO_2_. OCR was detected under basal conditions, followed by the sequential injection of 1 μM oligomycin (MedChemExpress; HY-16589), 2.5 μM BAM15 (HY-110284), and 1 μM antimycin A/Rotenone (HY-B1756) in a SeahorseXF Pro Analyzer (Agilent Technologies).

### Complex I activity assay.

Complex I activity was measured using the complex I Enzyme Activity Assay Kit (Abcam; ab109721). Between 1 × 10^7^ to 2 × 10^7^ cells were harvested for protein extraction and quantification. Sample concentration was adjusted to 1,000 μg/mL with incubation solution buffer, and 200 μL of sample/well was loaded and incubated in agitation for 3 hours at RT. Finally, 200 μL/well of Assay Solution (1X Dilution Buffer, 20X NADH, 10X Dye) was added, and 450 nm absorbance was measured every 40 seconds for 30 minutes.

### Zebrafish xenograft experiments.

Casper zebrafish (*Danio rerio H.*), from the Zebrafish International Resource Center, were maintained in recirculating tanks according to standard procedures. AML cells were stained with CM-Dil (Molecular Probes, Life Technologies; V-22888) and microinjected into the circulation through the Cuvier duct of 2 days after fecundation tricaine-anesthetized larvae (200 cells/larva). This xenograft does not require preconditioning since zebrafish larvae develop adaptive immunity at 4–6 weeks after fecundation (50). Larvae were incubated for 2 hours at 28°C in water. Next, fluorescence-positive larvae were selected, and drugs were added to water. Three days after injection, images were taken using the M2015-FA fluorescence microscope equipped with a DFC365FX camera (Leica), and cell infiltration index was calculated by counting the number of cells present in the caudal hematopoietic tissue.

### AML murine s.c. models.

HL-60-R and OCI-AML3-R cell lines (5 × 10^5^) were resuspended in PBS and Matrigel (Corning; 356255) (1:1) and injected s.c. into the flanks of 8- to 10-week-old female NOD.Cg-PrkdcscidIl2rgtm1Wjl/SzJ (NSG) mice (The Jackson Laboratory, USA) forming a localized mass that can be monitored using a caliper according to the formula (length × width²)/2. The term “tumor volume” refers to the size of the s.c. leukemic cell mass, which allows us to follow and measure the leukemic cell proliferation in vivo. Once tumor volumes reached 100–200 mm^3^, mice were randomized and given vehicle (10% N,N-dimethylacetamide [DMA]), 10% Kolliphor HS 15 (Sigma; 42966), 80% H_2_O), venetoclax plus azacitidine (100 and 3 mg/kg respectively, in vehicle) and/or RPT04402 (10 mg/kg in vehicle) by oral gavage. Treatment was administered until tumor volumes reached 3,000 mm^3^. Tumor volumes were assessed by caliper measurement (L × W^2^/2).

### Statistics.

Data were analyzed using GraphPad Prism v8 with parametric (Unpaired 2-tailed *t* test, 1-way or 2-way ANOVA) or nonparametric statistical tests (Mantel-Cox, Kruscal-Wallis) as detailed in each figure legend. Data from in vitro experiments represents the outcomes of at least 3 independent biological and technical replicates. In vivo experiments were performed as specified in the corresponding figures/figure legends. A *P* value less than 0.05 was considered significant.

### Study approval.

AML primary samples were collected in accordance with the Declaration of Helsinki, with approval of the Ethics Committee Investigación Clínica de Navarra (PI2018/32). Patients gave written informed consent. Zebrafish larvae younger than 5 days after fertilization are not considered animal experimentation under European and Spanish regulations. In vivo mouse experiments were approved by the University of Michigan IACUC. Guidelines for the proper use of animals in research and animal welfare were followed.

### Data availability.

RNA-seq data have been deposited in NCBI’s Gene Expression Omnibus (GEO) (51) and are accessible through GEO Series accession no. GSE291351. Mass spectrometry data and search results files were deposited in the ProteomeXchange Consortium via the JPOST partner repository (52) with the identifier PXD062308 for ProteomeXchange and JPST003729 for jPOST. Survival and RNA-seq data from patients with AML who were analyzed in this study were obtained from the OHSU Beat AML project (18). Derived data supporting the findings of this study are available within the article and the Supplemental Data Values file. Values for all original data points in graphs are reported in the Supporting Data Values file.

## Author contributions

SRM and IP contributed equally and are both co–first authors. The order of the co–first authors was determined by mutual agreement among the authors. Conceptualization was contributed by SRM, IP, CV, CMO, MDO, and GN. Data curation was contributed by SRM, JFI, and ES. Formal analysis was contributed by SRM, IP, AML, JFI, and ES. Methodology was contributed by SRM, IP, AML, NM, CV, BT, KB, CMO, MLC, VM, MDO, and GN. Investigation was contributed by SRM, IP, AML, NM, KB, JFI, and ES. Visualization was contributed by SRM, IP, and MDO. Software was contributed by JFI and ES. Validation was contributed by SRM, IP, and AML. Resources were contributed by AML, ATL, MCM, MLC, VM, JFI, and ES. Funding acquisition was contributed by MDO and GN. Project administration was contributed by SRM, IP, MDO, and GN. Supervision was contributed by VM, MDO, and GN. Writing of the original draft was contributed by SRM, IP, MDO, and GN. Review and editing were contributed by SRM, IP, MDO, and GN.

## Conflict of interest

KB received personal fees from Rappta Therapeutics during the conduct of this work. CMO reports receiving consulting fees from Rappta Therapeutics. GN had an equity stake in RAPPTA Therapeutics and serves as a consultant for RAPPTA. GN has served on the SAB for Hera BioLabs. No disclosures were reported by other authors.

## Funding support

This work was supported by the following:

- Instituto de Salud Carlos III (FIS) – Acción Estratégica en Salud (PI20/01558) (MDO).
- Rappta Therapeutics funding.
- Fundación Séneca, Agencia de Ciencia y Tecnología de la Región de Murcia (21887/PI/22) (VM).
- MCIN/AEI/10.13039/501100011033 (2020-113660RB-I00) (VM).
- Fundación para la Investigación Médica Aplicada (Programa de ayudas predoctorales de investigación biomédica AC FIMA) and Gobierno de Navarra (Ayudas predoctorales para la realización de Programas de Doctorado de Interés para Navarra) (SRM).

## Supplementary Material

Supplemental data

Unedited blot and gel images

Supplemental tables 1-13

Supporting data values

## Figures and Tables

**Figure 1:**
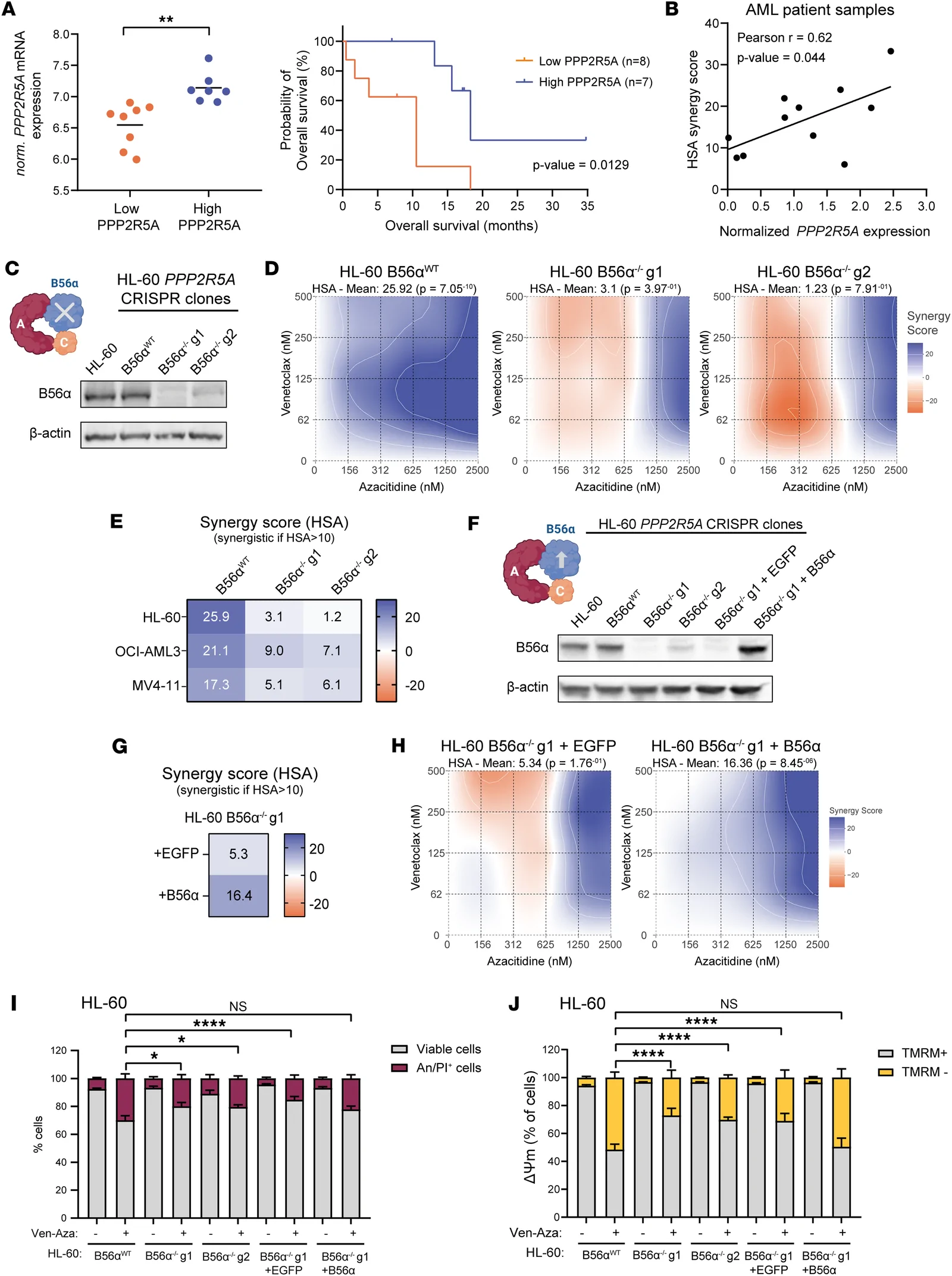
Low B56α expression levels impair the response to venetoclax-azacitidine treatment. (**A**) mRNA *PPP2R5A* expression levels in Ven-Aza–treated patients from the OHSU Beat AML cohort grouped into low (*n* = 8) or high *PPP2R5A* expressing groups (*n* = 7). Statistical significance was determined by unpaired *t* test. On the right, survival curves comparison of low versus high *PPP2R5A* expressing groups analyzed by the log-rank (Mantel-Cox) test. (**B**) Correlation scatter plot between *PPP2R5A* mRNA expression and highest single agent (HSA) synergy scores of 11 AML primary cells. (**C**) Graphical representation and Western blot analysis of B56α protein expression in HL-60 parental cell line, *PPP2R5A* CRISPRCas9 WT clone (B56α^WT^), and sublines with B56α genetic inactivation (B56α^–/–^ g1 and B56α^–/–^ g2). (**D**) HSA synergy scores of each drug combination in HL-60 B56α^WT^ and B56α^–/–^ g1 and B56α^–/–^ g2 clones. (**E**) Heatmap representation of HSA synergy score means of Ven-Aza combination in HL-60, OCI-AML3, and MV4-11 B56α^WT^ and B56α^–/–^ g1 and B56α^–/–^ g2 clones (48 hours after treatment). (**F**) Graphical representation and Western blot analysis of B56α protein expression in HL-60 parental cell line, B56α^–/–^ clones, and B56α^–/–^ g1 after viral transduction with EGFP (B56α^–/–^ g1 + EGFP) or *PPP2R5A* (B56α^–/–^ g1 + B56α) vectors. (**G** and **H**) Heatmap representation of HSA synergy score means of Ven-Aza combination and HSA synergy scores of each drug combination in HL-60 B56α^–/–^ g1 + EGFP and B56α^–/–^ g1 + B56α cells. (**I** and **J**) Average percentages ± SEM of viable and annexin V/propidium iodide positive (AnV/PI^+^) cells and with mitochondrial membrane potential loss in HL-60 B56α^WT^, B56α^–/–^, B56α^–/–^ g1 +EGFP, and B56α^–/–^ g1 + B56α cells after 48 and 24 hours upon Ven-Aza treatment (125 nM and 625 nM, respectively). Data were analyzed by 2-way ANOVA and Tukey post hoc test. In vitro results were obtained from 3 biological replicates. *P < 0.05, **P < 0.01, ***P < 0.001, ****P < 0.0001**.**

**Figure 2:**
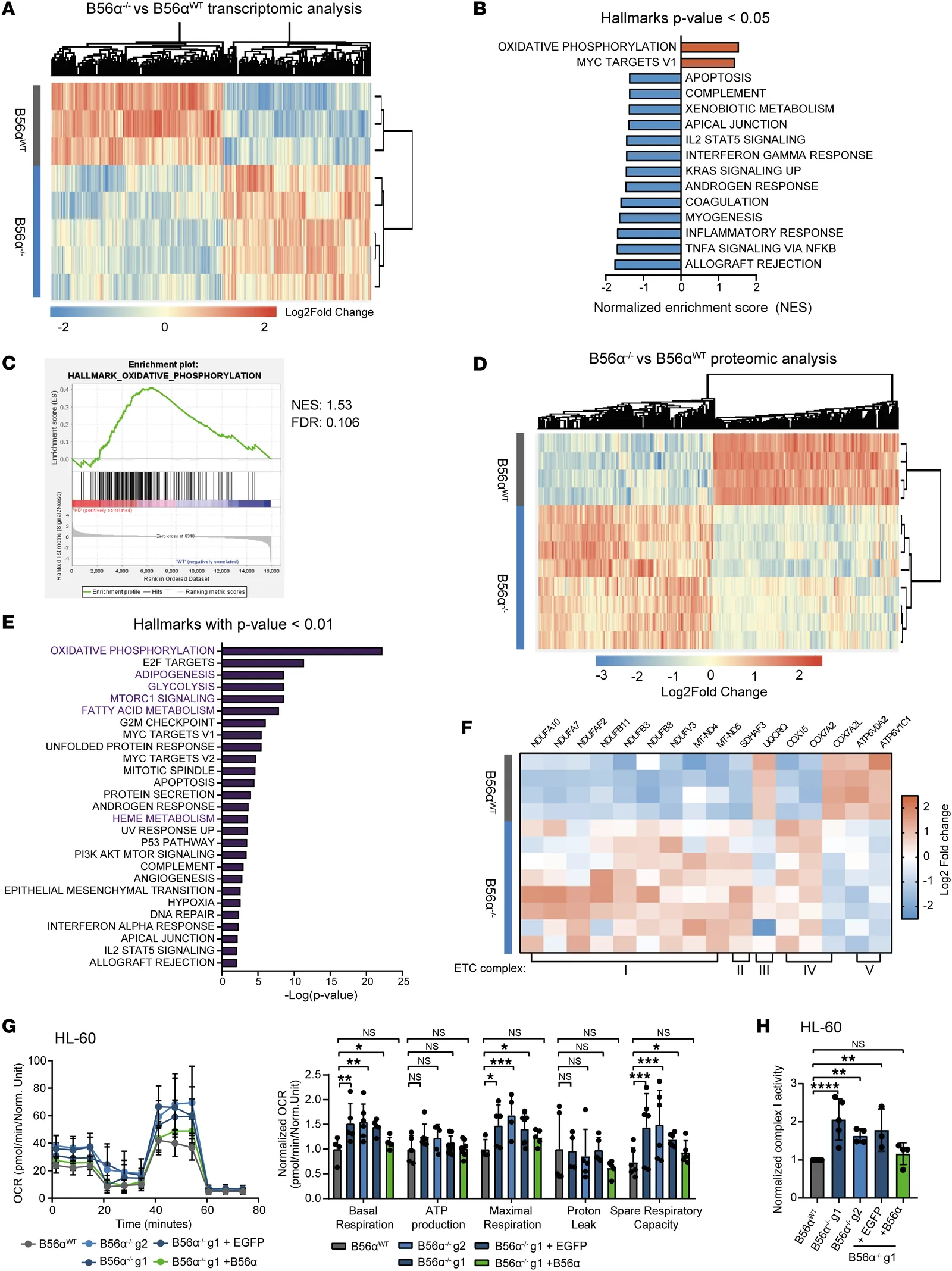
Transcriptomics and proteomics analyses indicate that OXPHOS is upregulated in HL-60 B56α^–/–^ models. (**A**) Heatmap representation of genes differentially upregulated (red) and downregulated (blue) at transcriptional level in HL-60 B56α^–/–^ clones compared with B56α^WT^ cells (*n* = ~3 per subline). (**B**) Hallmark gene-set enrichment analysis of differential gene expression in B56α^–/–^ clones. Positive normalized enrichment score (NES) represents upregulated hallmark gene sets, while negative NES indicates the gene sets downregulated with *P* < 0.05. (**C**) Enrichment plot of upregulated OXPHOS hallmark gene set with its NES and false discovery rate (FDR) in B56α^–/–^ clones compared with B56α^WT^ cells. (**D**) Heatmap representation of proteomics analyses in HL-60 B56α^–/–^ clones compared with B56α^WT^ cells (*n* = 4 per subline). (**E**) Hallmark gene set pathways altered in B56α^–/–^ clones compared with B56α^WT^ cells. Hallmarks in purple represent metabolism-related pathways. Statistical significance is expressed as –Log_10_(*P* value). (**F**) Heatmap representation of the differential expression of the mitochondrial proteins from the ETC complex in HL-60 B56α^WT^ and B56α^–/–^ clones (*n* = 4 per subline). (**G**) Dynamic representation of oxygen consumption rate (OCR) in HL-60 B56α^WT^, B56α^–/–^, and reconstituted B56α clones at basal conditions obtained from 3 biological replicates. (Right) Comparison of the basal respiration, ATP production, maximal respiratory capacity, proton leak, and spare respiratory capacity of the clones expressed as fold change of the normalized HL-60 B56α^WT^ clone values. Data represent mean ± SD and were analyzed by 2-way ANOVA and Tukey post hoc test. (**H**) Bar graph show average ± SD mitochondrial complex I activity of HL-60 clones measured as the change in absorbance per minute per amount of protein loaded and expressed as the fold change of HL-60 B56α^WT^ clone values. These results were obtained from 3 biological replicates. Statistical significance was determined by 1-way ANOVA and Tukey post hoc test analyses.

**Figure 3:**
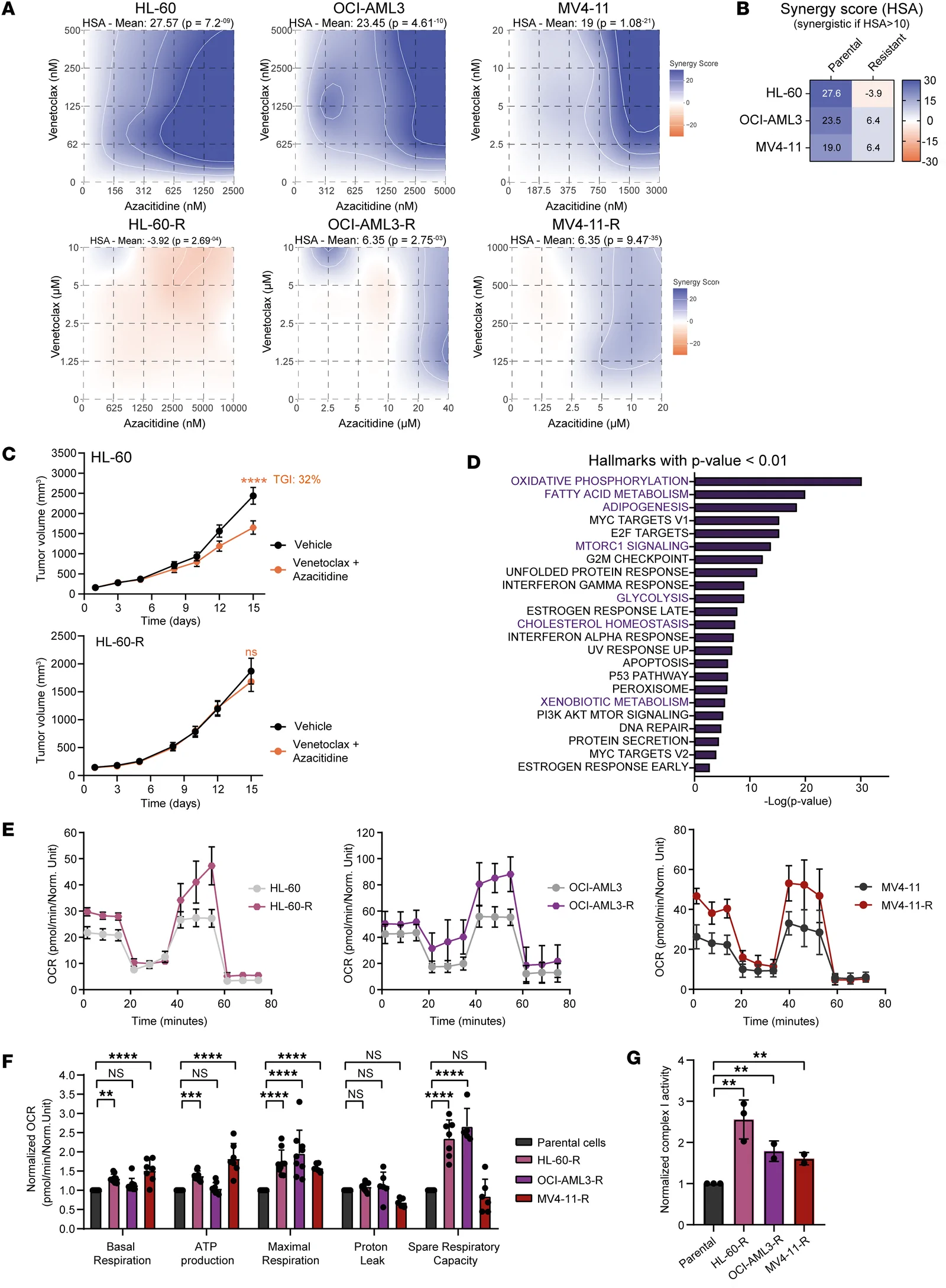
AML Venetoclax-azacitidine resistant models upregulate OXPHOS. (**A** and **B**) Two-dimensional contour plots represent highest single agent (HSA) synergy scores of each drug combination (*y* axis: venetoclax; *x* axis: azacitidine) and heatmap representation of HSA synergy score means of Ven-Aza combination in HL-60, OCI-AML3, and MV4-11 parental and Ven-Aza–resistant cell lines (48-hour treatment). (**C**) Time-dependent tumor growth upon vehicle or Ven-Aza treatment of injected NSG mice with HL-60 and HL-60-R cell lines. Doses: 100 and 3 mg/kg of venetoclax and azacitidine, respectively (*n* = ~15 mice per group). Statistical significance was determined by 2-way ANOVA and Tukey post hoc test. Data represent mean ± SEM. (**D**) Hallmark gene set pathways altered in HL-60 Ven-Aza resistant cell line compared with HL-60 parental. Hallmarks in purple represent metabolism-related pathways. Statistical significance is expressed as –Log_10_(*P* value). (**E**) Dynamic representation of oxygen consumption rate (OCR) in HL-60, OCI-AML3, and MV4-11 parental and Ven-Aza–resistant cell lines under basal conditions. (**F**) Bar graphs show comparison of the basal respiration, ATP production, maximal respiratory capacity, proton leak, and spare respiratory capacity of parental and Ven-Aza–resistant cell lines expressed as fold change of the normalized parental cell values for each cell line, respectively. Data were analyzed by 2-way ANOVA and Tukey post hoc test. (**G**) Bar graph represents mitochondrial complex I activity in parental and Ven-Aza–resistant cell lines expressed as the fold change of their respective parental cell line. Statistical significance was determined by 1-way ANOVA and Tukey post hoc test analyses. In vitro results were obtained from at least 3 biological replicates and represent mean ± SD.

**Figure 4:**
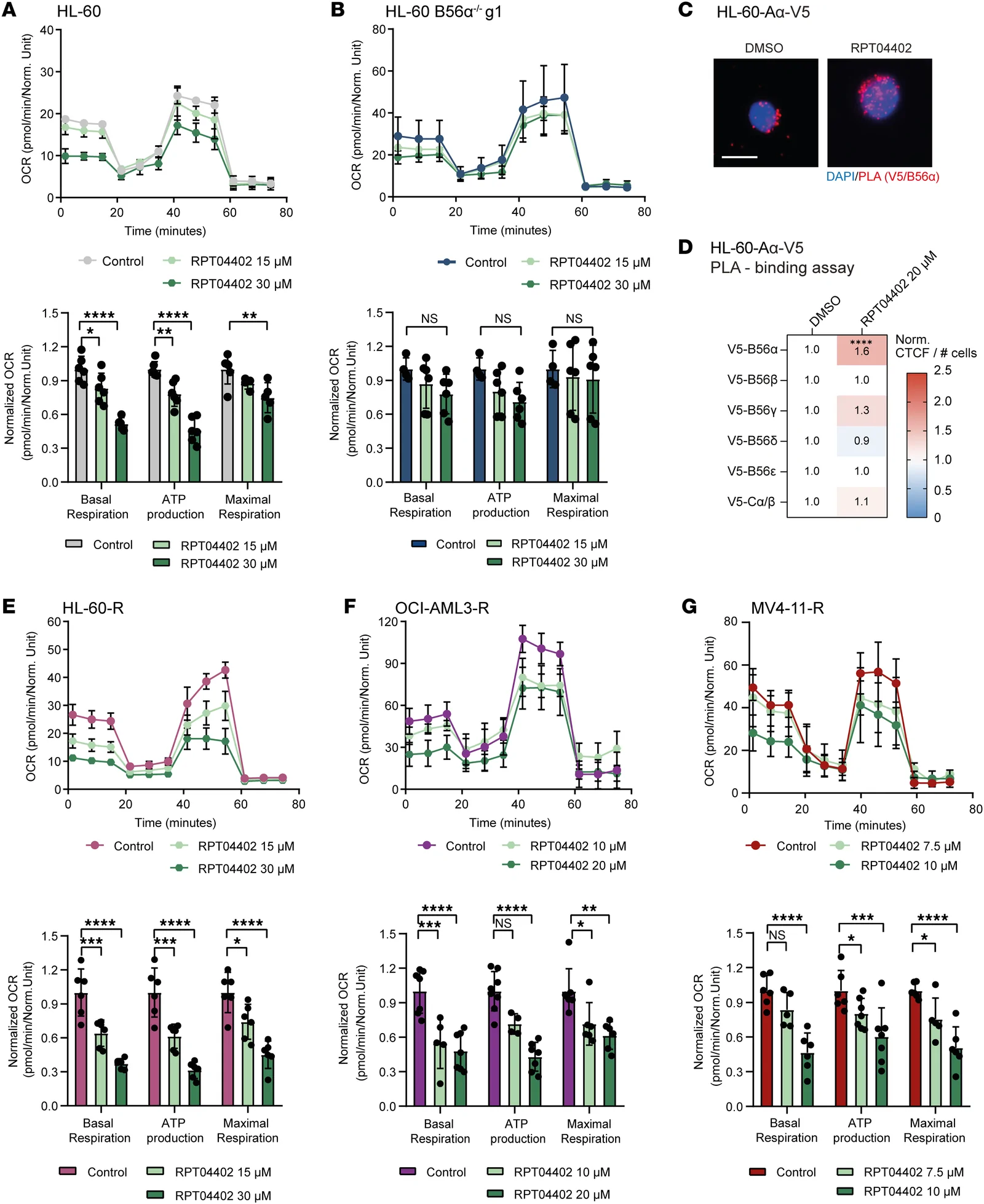
PP2A-B56α heterocomplex stabilization decreases OXPHOS in venetoclax-azacitidine AML-resistant models. (**A**, **B**, **E**–**G**)Seahorse extracellular flux analysis shows dynamic representation of oxygen consumption rate (OCR) in HL-60 (**A** HL-60 B56α^–/–^ g1 (**B**), HL-60-R (**E**), OCI-AML3-R (**F**), MV4-11-R cell lines (**G**) under basal conditions and treated with increasing concentrations of RPT04402 (12-hour treatment). Bar graphs show that RPT04402 treatment decreases basal respiration, ATP production, and maximal respiratory capacity of parental and Ven-Aza–resistant AML cell lines in a dose-dependent manner, expressed as a fold change of the control. (**C**) Representative images of the proximity ligation analysis (PLA) targeting the interaction between Aα-V5 tagged and B56α subunit (Texas Red) in the absence or presence of RPT04402 (20 μM). Experiment performed on HL-60 cells transfected with Aα-V5. Nuclei stained with DAPI. All images 40x magnification. Scale bar: 12.5 μm. (**D**) Relative quantification of PLA fluorescence signal of either B56α, B56β, B56γ, B56δ, B56ε, or Cα/β measured by calculating the corrected total cell fluorescence (CTCF) of multiple images (7–10 images) per biological replicate (*n* = 3). Data were analyzed by 2- (**A**, **B**, and **E**–**G**) or 1-way (**D**) ANOVA and Tukey post hoc test. All experiments were performed in 3 independent biological replicates. Data represent mean ± SD.

**Figure 5:**
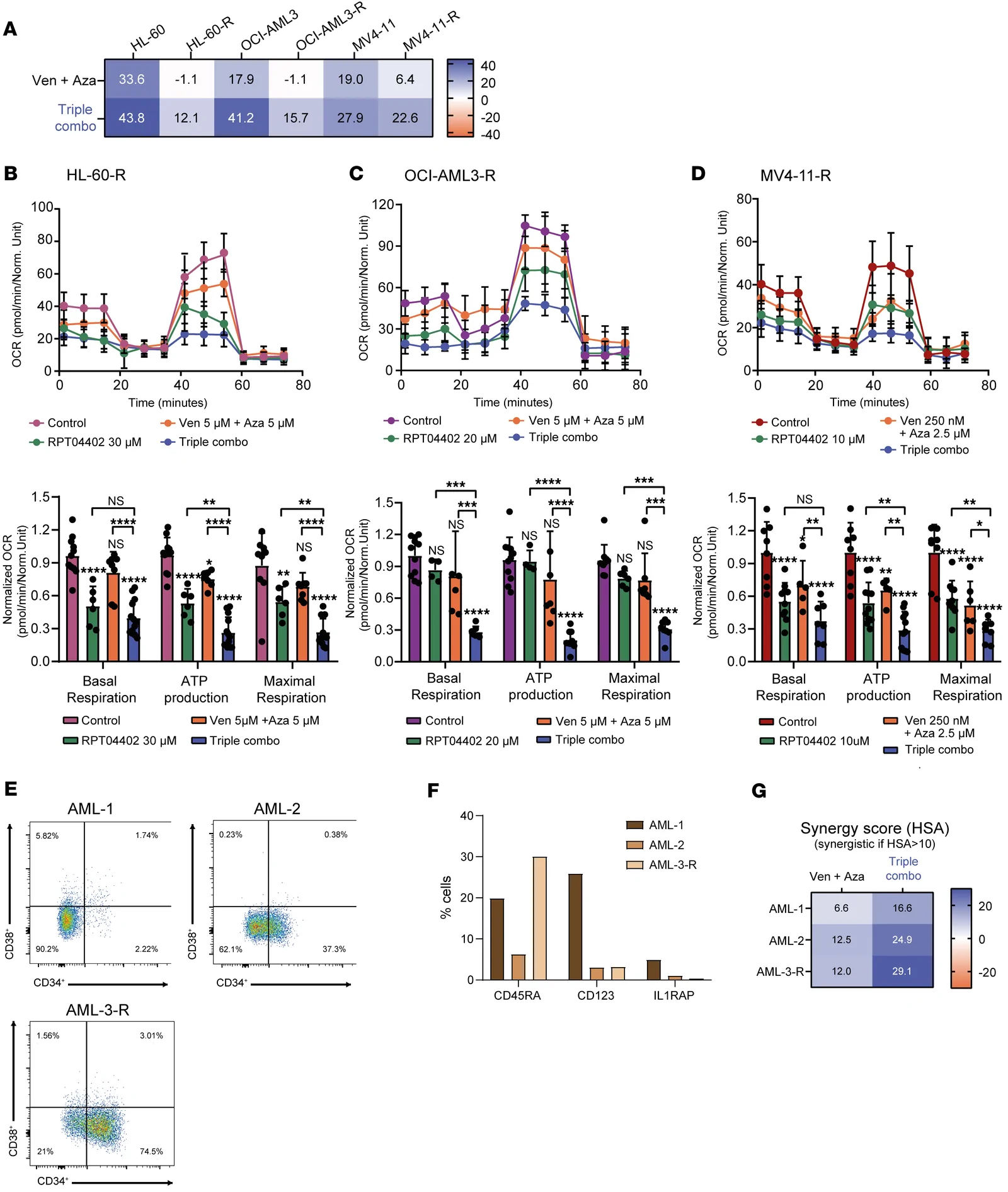
RPT04402 combined with venetoclax and azacitidine disrupts OXPHOS and overcomes venetoclax-azacitidine resistance in AML. (**A**) Heatmap representation of HSA synergy score means for Ven-Aza treatment and the triple therapy (Ven-Aza–RPT04402) in parental and Ven-Aza–resistant HL60, OCI-AML3, and MV4-11 cell lines (48-hour treatment) (synergistic when HSA > 10). (**B**–**D**) Dynamic representation of oxygen consumption rate (OCR) in HL-60-R (**B**), OCI-AML3-R (**C**), and MV4-11-R cell lines (**D**) at baseline (control) and after 12 hours of RPT04402, Ven-Aza, and Ven-Aza–RPT04402 (Triple combo) treatment. Bar graph represents basal respiration, ATP production, and maximal respiratory capacity levels expressed as fold change of control. RPT04402 concentrations for Triple combo treatment in HL-60-R, OCI-AML3-R, and MV4-11-R cell lines were 30, 15, and 10 μM, respectively. Asterisks on the top of each bar indicate the statistical significance of each condition compared with the control. Results were obtained from 3 biological replicates. Statistical significance was determined by 2-way ANOVA and Tukey post hoc test. Data represent mean ± SD. (**E** and **F**) Flow cytometry characterization of AML-1, AML-2, and AML-3-R primary samples of CD34/CD38 markers and quantification of the percentage of cells expressing CD45RA, CD123, and IL1RAP markers. (**G**) Heatmap representation of HSA synergy score means of Ven-Aza and Ven-Aza–RPT04402 treatment in 3 AML primary samples (60-hour treatment) obtained from ~ 2 biological replicates. *P < 0.05, **P < 0.01, ***P < 0.001, ****P < 0.0001.

**Figure 6:**
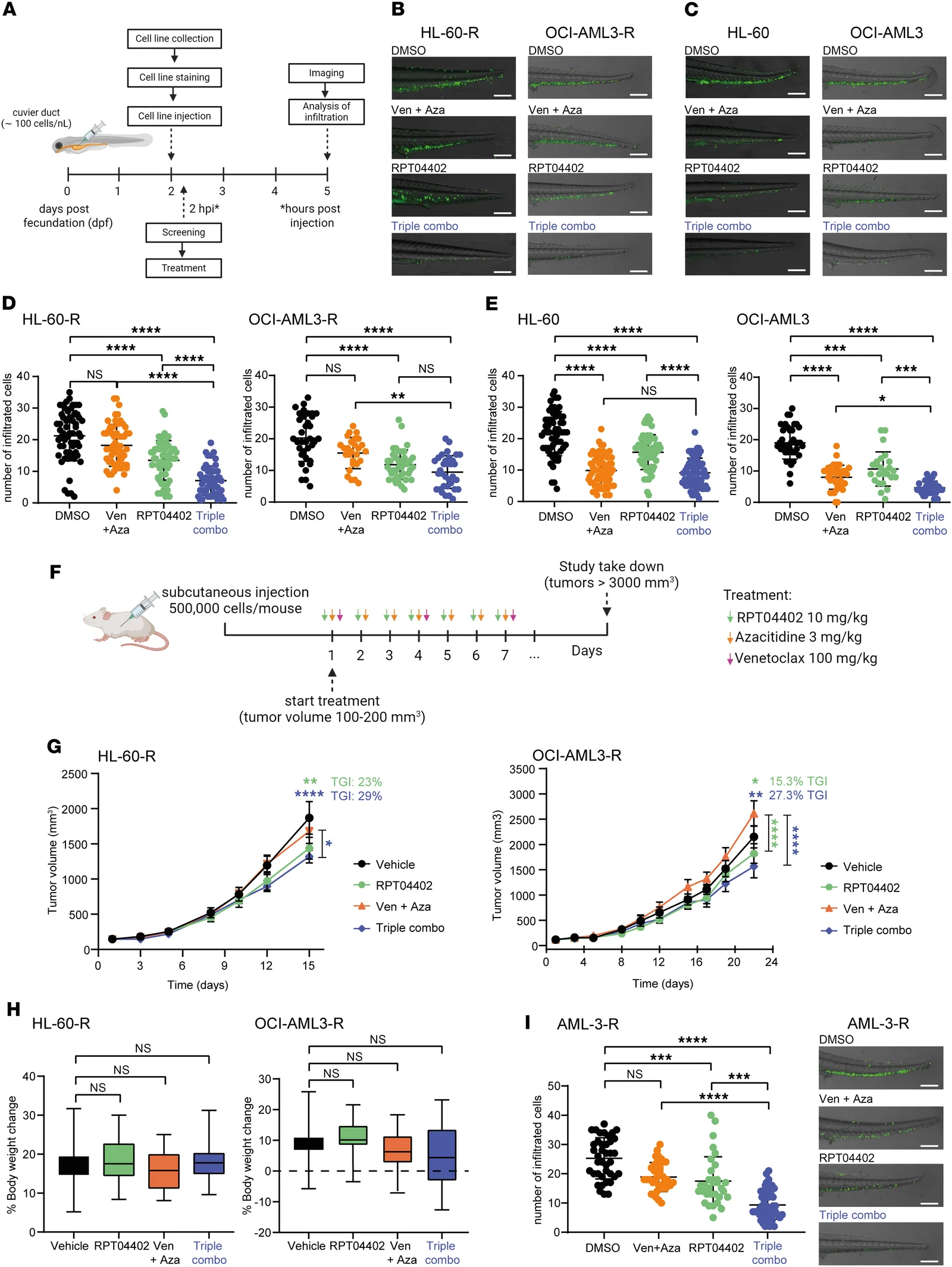
RPT04402 improves the response to venetoclax-azacitidine therapy in resistant models in vivo. (**A**) Schematic representation of zebrafish xenotransplantation and treatment workflow. Drug doses: 500 nM Ven, 1 μM Aza, and/or 20 μM RPT04402. (**B** and **C**) Representative images of HL-60-R and OCI-AML3-R (**B**) as well as HL-60 and OCI-AML3 cell lines (**C**) in xenograft larvae tail at 72 hours postinjection (hpi). AML cells were tracked as green-emitting fluorescent cells. (**D** and **E**) Cell invasion capacity of parental and Ven-Aza resistant cell lines were measured as the number of cells present in the caudal hematopoietic tissue after 70 hours of treatment. Each point represents one zebrafish larvae. Statistical significance was determined by 1-way ANOVA and Tukey post hoc test in HL-60 and HL-60-R cells, and by Kruskal-Wallis and Dunn post hoc test analyses for OCI-AML3 and OCI-AML3-R cell lines. (**F**) Schematic representation of in vivo subcutaneous efficacy study (detailed explanation in methods section). (**G**) Time-dependent tumor growth upon treatment of injected NSG mice with HL-60-R and OCI-AML3-R cell lines (*n* = ~15 mice per group). TGI, tumor growth inhibition. Statistical significance was determined by 2-way ANOVA and Tukey post hoc test. Data represent mean ± SEM. (**H**) Percentages of body weight change of NSG mice injected with HL-60-R and OCI-AML3-R cell lines after 15 and 22 days of treatment, respectively. Statistical significance was determined by 1-way ANOVA and Tukey post hoc test. Treatment doses: 10, 100, and 3 mg/kg for RPT04402, venetoclax, and azacitidine, respectively. (**I**) Cell invasion capacity of AML-3-R cells (72-hour treatment). Statistical significance was determined by Kruskal-Wallis and Dunn post hoc test analyses. On the right, representative images of AML-3-R cells of zebrafish PDX tail at 72 hours post-injection. Scale bar: 0.25 mm.

## Version 1 09/15/2026

In-Press Preview

## Version 2 10/22/2026

Electronic publication
