# Supplementary material for: Stabilization of the PP2A-B56α complex overcomes venetoclax-azacitidine resistance by impairing OXPHOS in AML
Source: JCI Insight. 2026 Sep 15;11(20):e205218. doi: 10.1172/jci.insight.205218 (PMC13726563; doi:10.1172/jci.insight.205218)
Supplement: Supplementary file 1 [file jciinsight-11-205218-s154.pdf]

Supplemental Data File

Stabilization of the PP2A-B56α complex overcomes Venetoclax-Azacitidine resistance by impairing OXPHOS in AML

Authors: Silvia Romero-Murillo*,[1-4] Irene Peris*,[5,6] Anna Maria Lucianò,[7-9] Nerea Marcotegui,[3] Carmen Vicente,[3] Brian Tran,[10] Kelsey Barrie,[5] Caitlin M. O'Connor,[5-6] Andrea Torres-López,[12] Maria C Mateos,[2,12] Maria L Cayuela,[11] Victoriano Mulero,[7-9] Joaquín Fernández-Irigoyen,[1,2] Enrique Santamaría,[1,2] Maria D Odero,[2-4] Goutham Narla.[5,6,13]

<u>Supplemental data file includes:</u>

Materials and Methods

Supplemental figures 1 to 16

References

<u>Other Supplemental material for this manuscript includes the following:</u>

Data files S1 to S13 (Excel files)

Supplemental methods

Colony-forming-unit assays

Normal human bone marrow derived CD34⁺ cells (2,500 cells/well, in 12 well-plates) were plated in MethoCult H4434 Classic medium (STEMCELL Technologies) supplemented with penicillin G (100 U/mL) and streptomycin (0.1 mg/mL). RPT04402 and/or venetoclax+azacitidine were added at different concentrations. After 12-14 days, colony-forming units (CFU) were counted and classified as CFU-M/GM, CFU-G or BFU-E. Representative pictures of colonies were taken using a Leica DM IL LED microscope.

CRISPR-Cas9 cell lines and ectopic expression

We used B56α and B55α HL-60 CRISPR-Cas9 clones and generated additional B56α CRISPR-Cas9 clones in OCI-AML3 and MV4-11 cell lines as previously described (1). Guide-RNAs (gRNAs) against *PPP2R5A* (B56α$^{-/-}$ gRNA 1: GAGTATGTTTCAACTAATCGTGG; B56α$^{-/-}$ gRNA-2: AAGAGCAACACTGAATGAACTGG) and *PPP2R2A* (B55α$^{-/-}$ gRNA-1: GTAGATGATGATGTAGCAGAAGG; B55α$^{-/-}$ gRNA 2: GAGGAGGGAATGATATTCAGTGG). The lentiviral vector used to overexpress B56α (pLV[Exp]-Puro-EF1A>hPPP2R5A[NM_006243.4], Vector ID: VB900132-5580hep) and the lentiviral control plasmid (pLV[Exp]-Puro-EF1A>EGFP, Vector ID: VB010000-9483amc) were constructed by VectorBuilder online platform and purchased from VectorBuilder Biotechnology Inc. (Chicago, IL, USA). To overexpress Aα-V5 tagged, the lentiviral vector pLX304 *PPP2R1A* (HsCD00444402) was purchased from DNASU (Arizona State University's Plasmid Repository). 10x lentiviral particles were produced in the Vector Core from University of Michigan (Ann Arbor, MI, USA). HL-60-B56α$^{-/-}$ g1 was transduced by spinoculation (90 minutes at 2,500 rpm and 30 ºC) with 1x viral supernatants and 8 µg/mL polybrene (Santa Cruz; sc-134220). Cells were washed with PBS 24 hours post-transduction. B56α overexpression was validated by western blot.

Mitochondrial membrane potential and AnnexinV/Propidium Iodide assays

Mitochondrial membrane potential changes (∆ψm) for apoptosis induction were measured by flow cytometry using the MitoProbe™ Tetramethylrhodamine, Methyl Ester, Perchlorate (TMRM) kit (Invitrogen; M20036) after 24 hours of treatment. Percentage of apoptotic cells was assessed using Dead Cell Apoptosis Kit with AnnexinV-FITC and Propidium Iodide (Invitrogen; V13242) after 48 hours of treatment. Fluorescence emissions were measured on a FACSCanto II flow cytometer (Becton Dickinson).

Western blot

Cultured or treated cells were lysated, prepared and detected for western blot analysis as previously described (1). The antibodies used were B55α (Cell Signaling Technology; 5689), B56β (Santa Cruz Biotechnology, sc-515676), B56γ (Santa Cruz Biotechnology, sc-374380), B56δ (Abcam, ab188323), B56ε (Abcam, ab198500), Cα/β (Abcam, ab106262), V5 (Cell Signaling, 13202), β-actin (Sigma, A1978), and B56α antibody provided by Dr.Ogris (Center for Medical Biochemistry, Max Perutz Labs, Medical University of Vienna, Vienna, 1030, Austria).

Real-Time qPCR

Total RNA was isolated using the High Pure RNA Isolation Kit (Roche; 11828665001) and reverse transcribed with the High-Capacity cDNA Reverse Transcription Kit (Applied Biosystems; 4368814). Real-time qPCR (RT-qPCR) reactions were run with a ViiA 7 Real-Time PCR System instrument (Applied Biosystems) according to the manufacturer's instructions. *PPP2R5A* expression was normalized to *HPRT* mRNA expression levels. Relative expression levels were calculated by the 2-ΔΔCt method. Primer sequences: *PPP2R5A* Fwd 5´-TTGGCCTCACATACAGTTGG-3´, *PPP2R5A* Rv 5´-TTCCCATAAATTCGGTGCAG-3´, *HPRT* Fwd 5´-TGACACTGGCAAAACAATG-3´, and *HPRT* Rv 5´-GGTCCTTTTCACCAGCAAG-3´.

RNA sequencing

RNA sequencing was conducted by the Genomics Unit of CIMA (Pamplona, Spain). Total RNA from HL-60-B56α clones was isolated in 3 biological replicates using the High Pure RNA Isolation Kit (Roche, 11828665001), and transcriptomic analyses were performed. Roughly 200 ng of high-quality total RNA was used for the transcriptomic interrogation of HL-60-B56α$^{-/-}$ g1 and g2 versus B56α^{WT} clones using Illumina's Stranded mRNA Prep ligation according to the manufacturer's instructions. Briefly, oligo(dT) magnetic beads were used to capture and purify poly-adenylated mRNA molecules from total RNA. The purified mRNA was then fragmented and reverse-transcribed to cDNA

using random primers. A second strand cDNA synthesis step removed the RNA template while incorporating dUTP in place of dTTP in order to preserve strand specificity. Next, double-stranded cDNA was A-tailed, then ligated to Illumina anchors bearing T-overhangs. PCR-amplification of the library allowed the barcoding of the samples with 10 bp dual indexes and the completion of Illumina sequences for cluster generation. Libraries were quantified with Qubit dsDNA HS Assay Kit (Invitrogen, Q32851), and their profile was examined using Agilent's HS D1000 ScreenTape Assay. Sequencing was carried out in an Illumina NextSeq2000 using paired-end, dual-index sequencing (Rd1: 59 cycles; i7: 10 cycles; i5: 10 cycles; Rd2: 59 cycles) at a depth of 30 million reads per sample.

Statistical and functional analysis

Statistical and functional analyses were performed with Gene Set Enrichment Analysis (GSEA) software (version 4.3.2) using the hallmark gene set collection (2–4). Normalized enrichment score (NES) and p-value < 0.05 were applied to evaluate enrichment magnitude and statistical significance, respectively. For statistical and functional analyses of the selected AML patients from the OHSU Beat AML cohort, mRNA sequencing data of the study were used (5).

Proteomics

Proteomic analyses of HL-60-B56α$^{-/-}$ g1 and g2 versus B56α^{WT} clones; and venetoclax-azacitidine resistant HL-60 versus parental cells (HL-60-R vs. HL-60) were conducted by the Proteomics Platform of Navarrabiomed (Pamplona, Spain) following previously described protocol (6).

Between 10-15×10^6 cells were harvested and washed with PBS 3 times in 4 biological replicates. Before processing, all samples were randomized, and experimental groups were balanced to avoid bias arising from the manipulation during the process. Cell pellets were homogenized in a lysis buffer (7 M urea, 2 M thiourea, 50 mM dithiothreitol (DTT)),

supplemented with protease (Roche; 11836153001) and phosphatase inhibitors (Roche; 4906845001). Proteins extracts were quantified and reduced with 20 nM DTT (room temperature, 30 minutes), alkylated with 30 mM iodoacetamide (room temperature, 30 minutes in the dark), diluted to 0.9 M with ABC, and digested with 1:20 w/w trypsin (Promega; V5117; 18 hours, 37 °C). Protein digestion was interrupted by acidification (pH < 6, acetic acid), and the resulting peptides were cleaned-up using Pierce™ Peptide Desalting Spin Columns (Thermo Fisher Scientific; 89852).

Dried-down peptide samples (10 µg) were reconstituted with 2% ACN-0.1% FA (Acetonitrile-Formic acid), spiked with internal retention time peptide standards (iRT, Biognosys), and quantified by NanoDropTM spectrophometer (Thermo Fisher Scientific) prior to LC-MS/MS analysis using an EASY-1000 nanoLC system coupled to an EZ-Exploris 480 mass spectrometer (Thermo Fisher Scientific.). Peptides were resolved using C18 Aurora column (75µm x 25cm, 1.6 µm particles; IonOpticks) at a flow rate of 300 nL/min using a 60-minutes gradient (50 oC): 2% to 5% B in 1 min, 5% to 20% B in 48 min, 20% to 32% B in 12 min, and 32% to 95% B in 1 min (A = FA, 0.1%; B = 100% ACN:0.1% FA). Peptides were ionized using 1.6 kV spray voltage at a capillary temperature of 275 °C. Sample data were acquired in Data independent acquisition (DIA) mode with full MS scans (scan range: 400 to 900 m/z; resolution: 60,000; maximum injection time: 22 ms; normalized AGC target: 300%) and 24 periodical MS/MS segments applying 20 Th isolation windows (0.5 Th overlap: Resolution: 15,000; maximum injection time: 22 ms; normalized AGC target: 100%). Peptides were fragmented using a normalized HCD collision energy of 30%.

Mass spectrometry data files were analyzed using Spectronaut (Biognosys) by direct DIA analysis (dDIA). MS/MS spectra were searched against the Uniprot proteome reference from Homo Sapiens database UP000005640 using standard settings. Carbamidomethyl was set as a fixed modification, and oxidation, acetyl (protein N-term),

deamidation, and Gln-> pyro-Glu as variable modifications for total protein analysis. Identifications were filtered by a 1% q-value.

Statistical and functional analyses

The obtained quantitative data for total protein were exported to Perseus software (version 1.6.15.0) for statistical analysis and data visualization (7). Unpaired Student's t-test was used for direct comparisons. Statistical significance was set at p-value <0.05 in all cases, and 1% peptide false discovery rate (FDR) threshold was considered. Differentially expressed proteins were considered significant when their absolute fold change was below 0.77 (downregulated proteins) and above 1.3 (up-regulated proteins) on a linear scale. Functional analyses were conducted using Metascape database and QIAGEN's Ingenuity Pathway Analysis (IPA) (version 107193442) (QIAGEN Inc) (8,9). Metascape extracts biological information associated with proteome functionality. Default settings (minimum overlap: 3; minimum enrichment: 1.5; $p<0.01$) and Hallmark Gene Sets were used for enrichment analysis. IPA software calculates significant values (p-values) between each biological event and the imported molecules based on Fisher's exact test ($p \leq 0.05$).

Supplemental Figure and legends

Figure S1

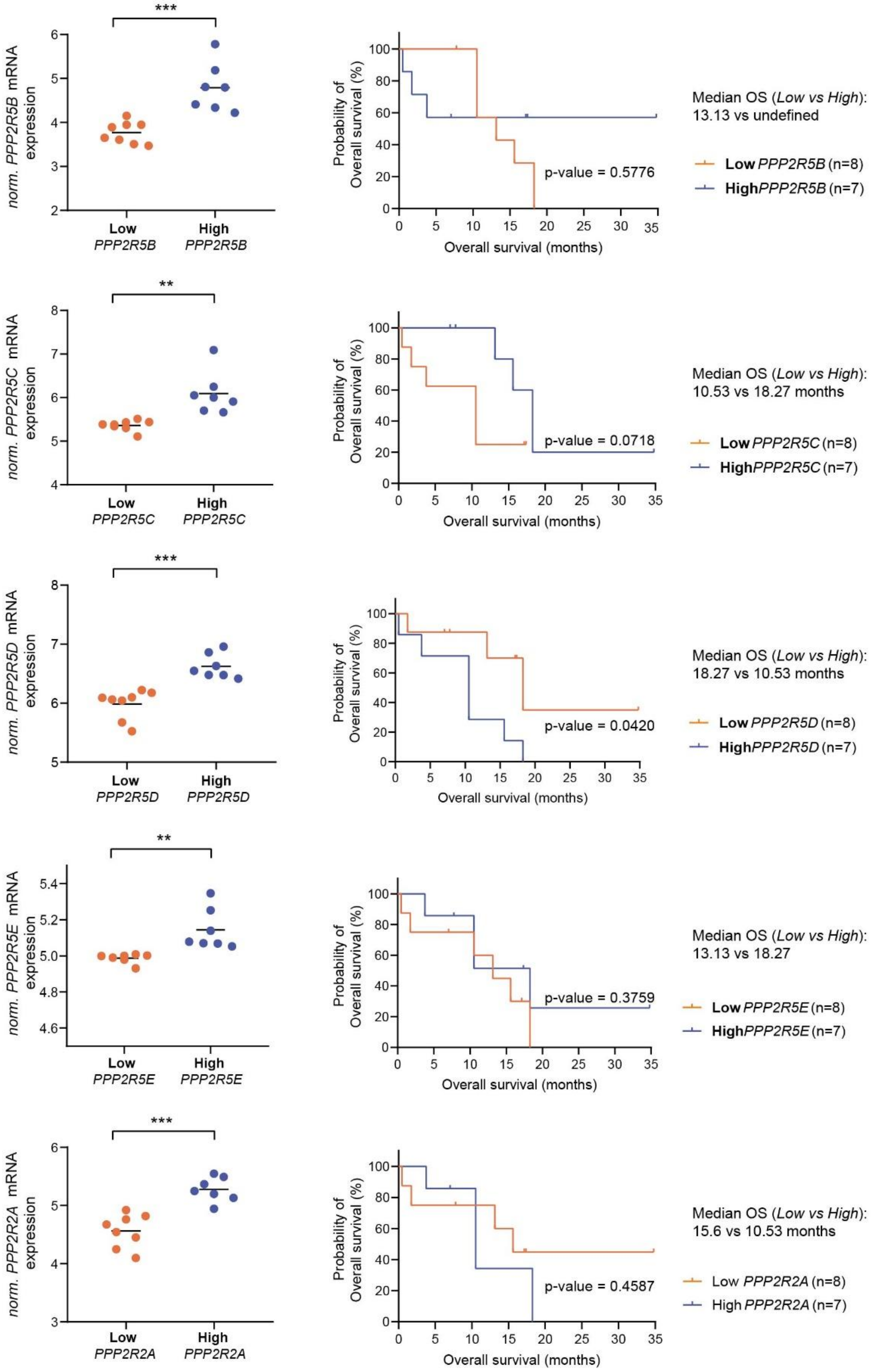

Supplemental Figure 1 (related to figure 1). mRNA expression levels of PP2A-B56 family B (*PPP2R5B, PPP2R5C, PPP2R5D, PPP2R5E)* and B55α (*PPP2R2A*) subunits

in patients treated with the Ven-Aza regimen from the OHSU Beat AML.2 cohort grouped into low or high expression of each PP2A B regulatory subunit (n=15). Statistical significance was determined by unpaired t-test. On the right, mRNA expression level-related survival curves of PP2A B subunits of AML Ven-Aza treated patients. Data were analyzed by the log-rank (Mantel-Cox) test. **p<0.05, **p<0.01, ***p<0.001, ****p<0.0001*.

Figure S2

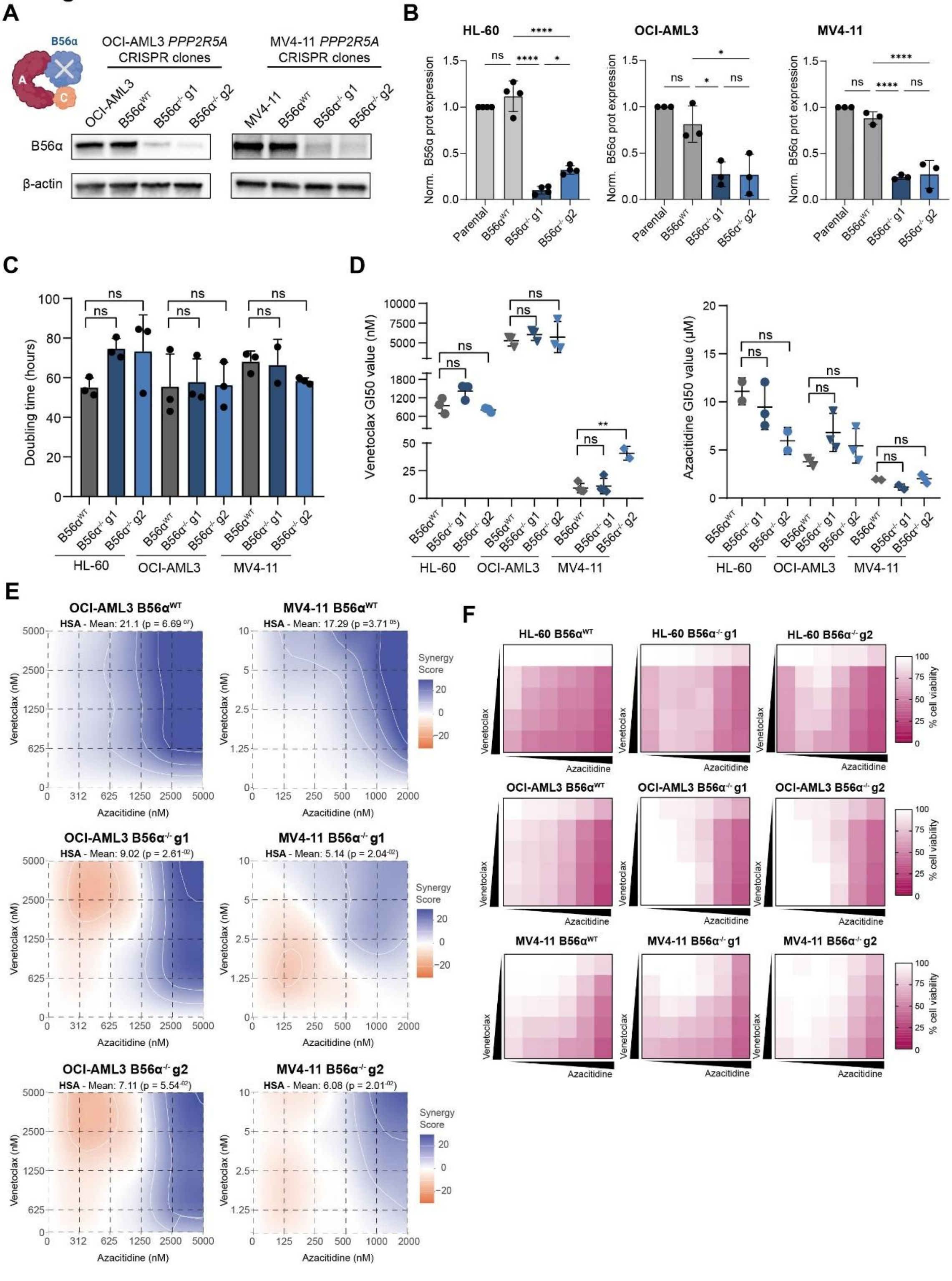

Supplemental Figure 2 (related to figure 1). (**A**) Graphical representation and (**B**) protein quantification from western blot analysis of B56α protein levels in HL-60, OCI-AML3, and MV4-11 parental cell lines, *PPP2R5A* CRISPRCas9 wild-type clones (B56α^{WT}), and sublines with B56α genetic inactivation (B56α$^{-/-}$ g1 and B56α$^{-/-}$ g2) expressed as the fold change of the parental values. (**C**) Bar graph represents the doubling time of HL-60, OCI-AML3, and MV4-11 B56α^{WT} and B56α$^{-/-}$ clones at baseline.

(**D**) GI50 values for venetoclax and azacitidine in HL-60, OCI-AML3, and MV4-11 B56α^{WT} and B56α$^{-/-}$ clones. Statistical significance was determined by one-way analysis of variance (ANOVA) and Tukey post hoc test analyses. Data represent mean ± SD. (**E**) Highest Single Agent (HSA) synergy scores of each drug combination (y-axis: venetoclax; x-axis: azacitidine) in OCI-AML3 and MV4-11 B56α^{WT} and B56α$^{-/-}$ clones. (**F**) Heatmap representations of viability percentages of Ven-Aza combination at increasing concentrations (48 hours of treatment) in HL-60, OCI-AML3, and MV4-11 B56α^{WT} and B56α$^{-/-}$ clones. **p<0.05, **p<0.01, ***p<0.001, ****p<0.0001.*

Figure S3

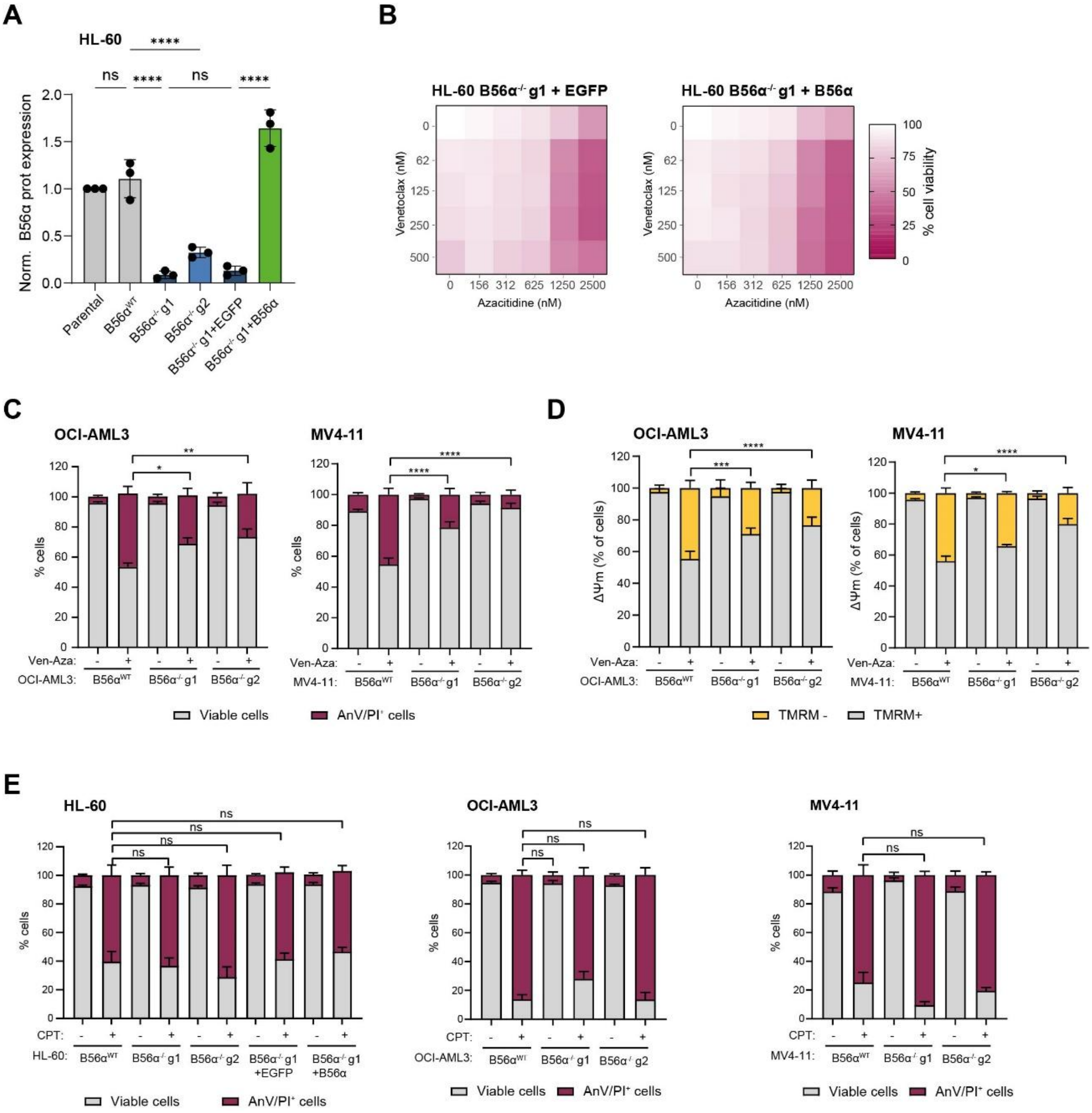

Supplemental Figure 3 (related to figure 1). (**A**) Normalized protein quantification from western blot analysis of B56α protein in HL-60 parental cell line, B56α$^{-/-}$ clones, and B56α$^{-/-}$ g1 after viral transduction with EGFP (B56α$^{-/-}$ g1 + EGFP) or *PPP2R5A* (B56α$^{-/-}$ g1 + B56α) vectors expressed as the fold change of HL-60 parental cell line. Data

represent mean ± SEM. (**B**) Heatmap representations of viability percentages of Ven-Aza combination at increasing concentrations (48 hours of treatment) in HL-60 B56α$^{-/-}$ g1 + EGFP and + B56α cells. (**C**) Average percentages of viable and annexin V/propidium iodide positive (AnV/PI^{+}) cells and (**D**) with mitochondrial membrane potential loss (Δψm) in OCI-AML3 and MV4-11 B56α^{WT} and B56α$^{-/-}$ cells after (C) 48 and (D) 24 hours upon Ven-Aza treatment (Ven 2.5 µM-Aza 625 nM in OCI-AML3 cells, and Ven 5 nM-Aza 375 nM in MV4-11). (**E**) Percentages of viable and annexin V/propidium iodide positive (AnV/PI^{+}) HL-60, OCI-AML3, and MV4-11 B56α^{WT} and B56α$^{-/-}$ cells after 48 hours upon camptothecin (CPT) treatment (10 µM) used as a positive control of apoptotic cell induction. Data were analyzed by two-way ANOVA and Tukey post hoc test. All graphs represent average ± SD results obtained from 3 independent biological replicates. Data were analyzed by two-way ANOVA and Tukey post hoc test. **p<0.05, **p<0.01, ***p<0.001, ****p<0.0001*, ns, not statistically different.

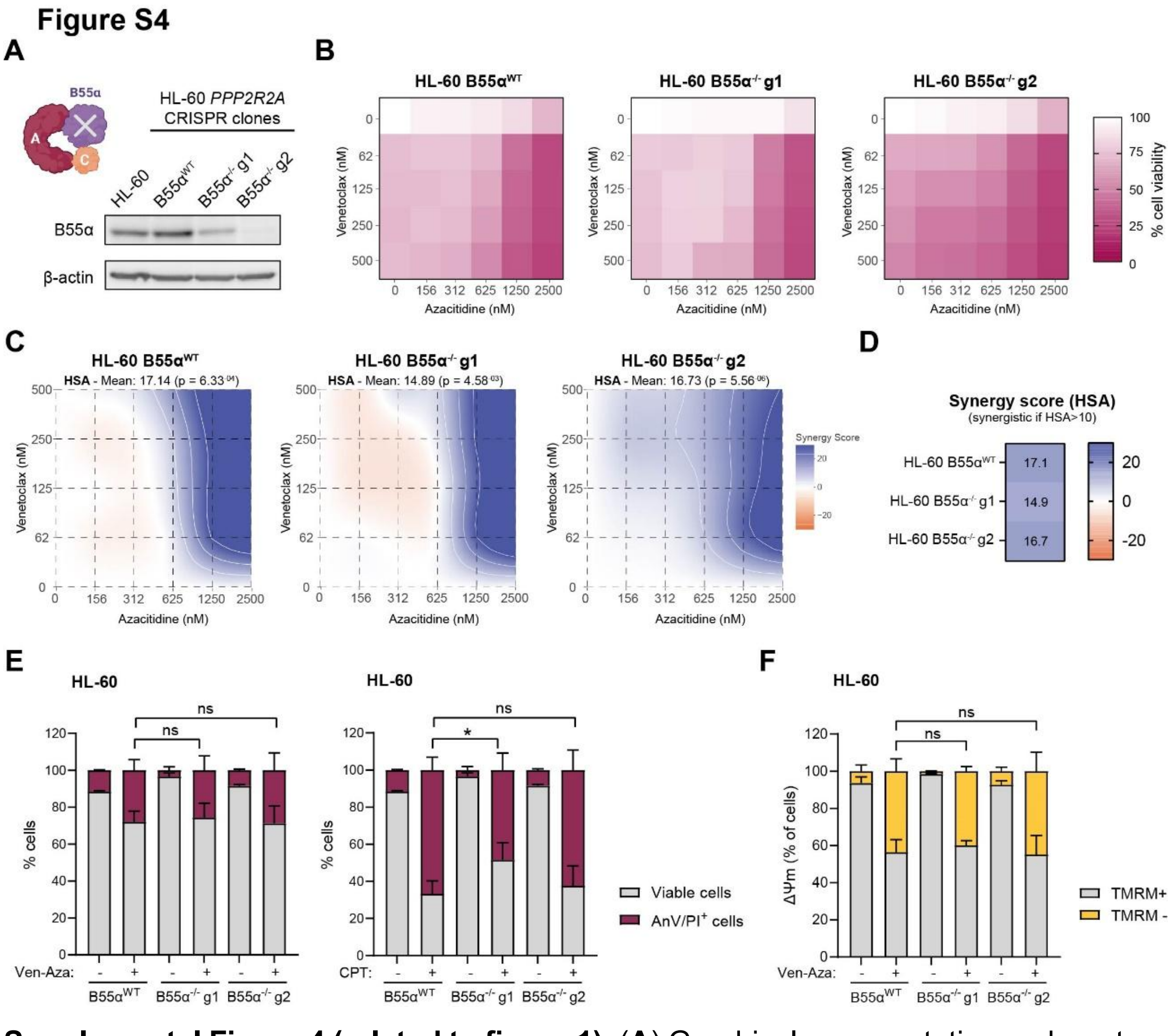

Supplemental Figure 4 (related to figure 1). (**A**) Graphical representation and western blot analysis of B55α protein expression in HL-60 parental cell line, *PPP2R2A* CRISPR/Cas9 wild-type clone (B55α^{WT}), and knockdown clones (B55α$^{-/-}$ g1 and B55α$^{-/-}$

g2). (**B**) Heatmap representations of viability percentages of venetoclax and azacitidine combination at increasing concentrations (48 hours of treatment) in HL-60 B55α^{WT} and B55α$^{-/-}$ clones. (**C**) Two-dimensional contour plots represent highest single agent (HSA) synergy scores of each drug combination (y-axis: venetoclax; x-axis: azacitidine) and (**D**) heatmap representation of HSA synergy score means in HL-60 B55α^{WT} and B55α$^{-/-}$ g1 and B55α$^{-/-}$ g2 clones (48 hours after treatment) (synergistic if HSA>10). (**E**) Percentages of viable and annexin V/propidium iodide positive (AnV/PI^{+}) HL-60 B55α^{WT} and B55α$^{-/-}$ cells after 48 hours upon Ven-Aza treatment (125 nM and 625 nM, respectively) (left graph) and camptothecin (CPT) treatment (10 μM) (right graph). (**F**) Percentage of HL-60 B55α^{WT} and B55α$^{-/-}$ cells with mitochondrial membrane potential loss (∆ψm) after 24 hours upon Ven-Aza treatment. All figures represent average results obtained from 3 independent biological replicates. Data were analyzed by two-way analysis of variance (ANOVA) and Tukey post hoc test. Data represent mean ± SEM. *p<0.05, **p<0.01, ***p<0.001, ****p<0.0001, ns, not statistically different.

Figure S5

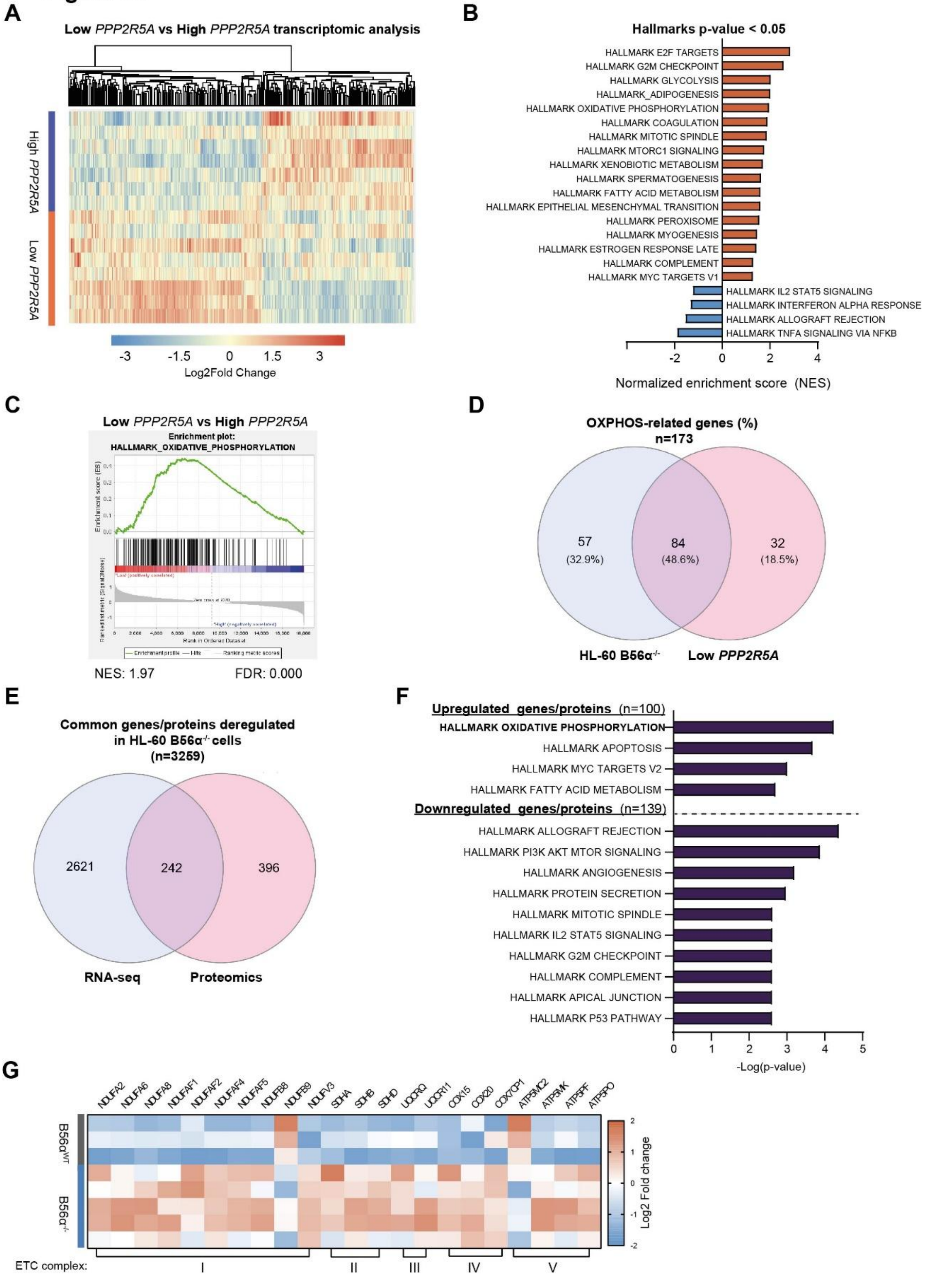

Supplemental Figure 5 (related to figure 2). (**A**) Heatmap representation of genes differentially up-regulated (red) and down-regulated (blue) at transcriptional level in patients with low *PPP2R5A* expression (n = 4) compared with high *PPP2R5A* expression

(n = 15) from the OHSU Beat AML.2 database. (**B**) Hallmark gene-set enrichment analysis of differential gene expression in low *PPP2R5A* group. Positive normalized enrichment score (NES) represents the upregulated hallmark gene sets, while negative NES indicates downregulated gene sets statistically significant (p-value<0.05). (**C**) Enrichment plot of up-regulated OXPHOS hallmark gene set with its NES and false discovery rate (FDR) in patients with low *PPP2R5A* compared with high *PPP2R5A* expression. (**D**) Venn diagram shows common altered genes within the OXPHOS pathway between HL-60 sublines lacking B56α subunit and patients with low *PPP2R5A* expression. (**E**) Common and differential altered genes/proteins in HL-60 sublines lacking B56α subunit. Figures created in *InteractiVenn.net* (10). (**F**) Hallmark gene set pathways deregulated resulted from the upregulated or downregulated common differentially expressed genes/proteins in HL-60 B56α^{WT} cells. Statistical significance is expressed as -Log$_{10}$(P) value (**G**) Heatmap representation of the differential expression of the mitochondrial genes from the Electron-Transporting Chain (ETC) complex in HL-60 B56α^{WT} and B56α$^{-/-}$ clones (n=3 per subline).

Figure S6

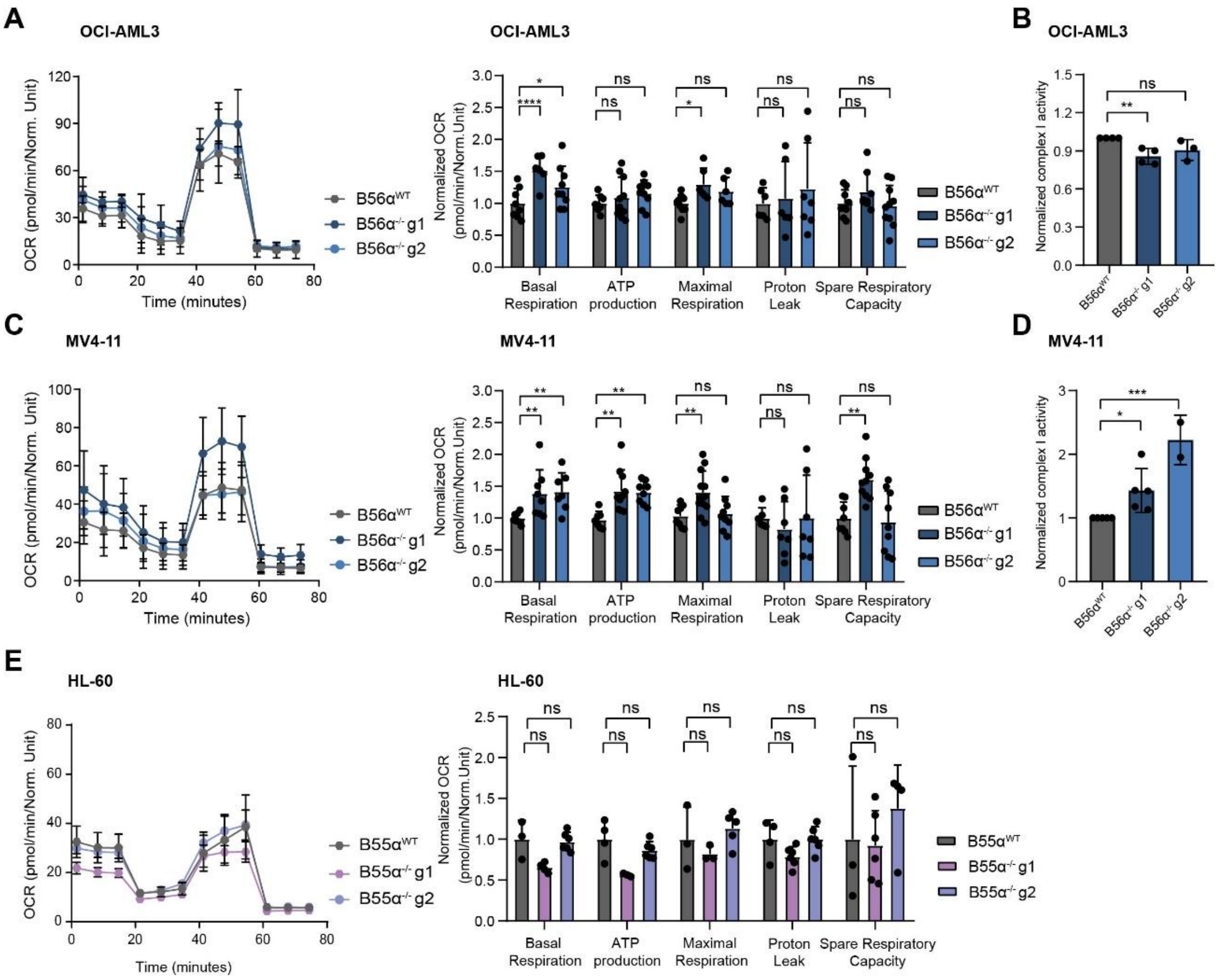

Supplemental Figure 6 (related to figure 2). Dynamic representation of oxygen consumption rate (OCR) in (**A**) OCI-AML3 and (**C**) MV4-11 B56α^{WT} and B56α$^{-/-}$ clones at

basal conditions. On the right, comparison of the basal respiration, ATP production, maximal respiratory capacity, proton leak, and spare respiratory capacity of the cells expressed as fold change of the normalized B56α^{WT} clone values. Data were analyzed by two-way analysis of variance (ANOVA) and Tukey post hoc test. Bar graph shows mitochondrial complex I activity of (**B**) OCI-AML3 and (**D**) MV4-11 B56α^{WT} and B56α$^{-/-}$ clones measured as the change in absorbance per minute per amount of protein loaded and expressed as fold change of the normalized B56α^{WT} clones' values (**E**) Dynamic representation of OCR in HL-60 B55α^{WT} and B55α$^{-/-}$ clones at basal conditions. On the right, comparison of the basal respiration, ATP production, maximal respiratory capacity, proton leak, and spare respiratory capacity of the clones expressed as fold change of the normalized HL-60 B55α^{WT} clone values. Results represent mean ± SD and were obtained from 3 biological replicates. Data were analyzed by two-way ANOVA and Tukey post hoc test. *p<0.05, **p<0.01, ***p<0.001, ****p<0.0001., ns, not statistically different.

Figure S7

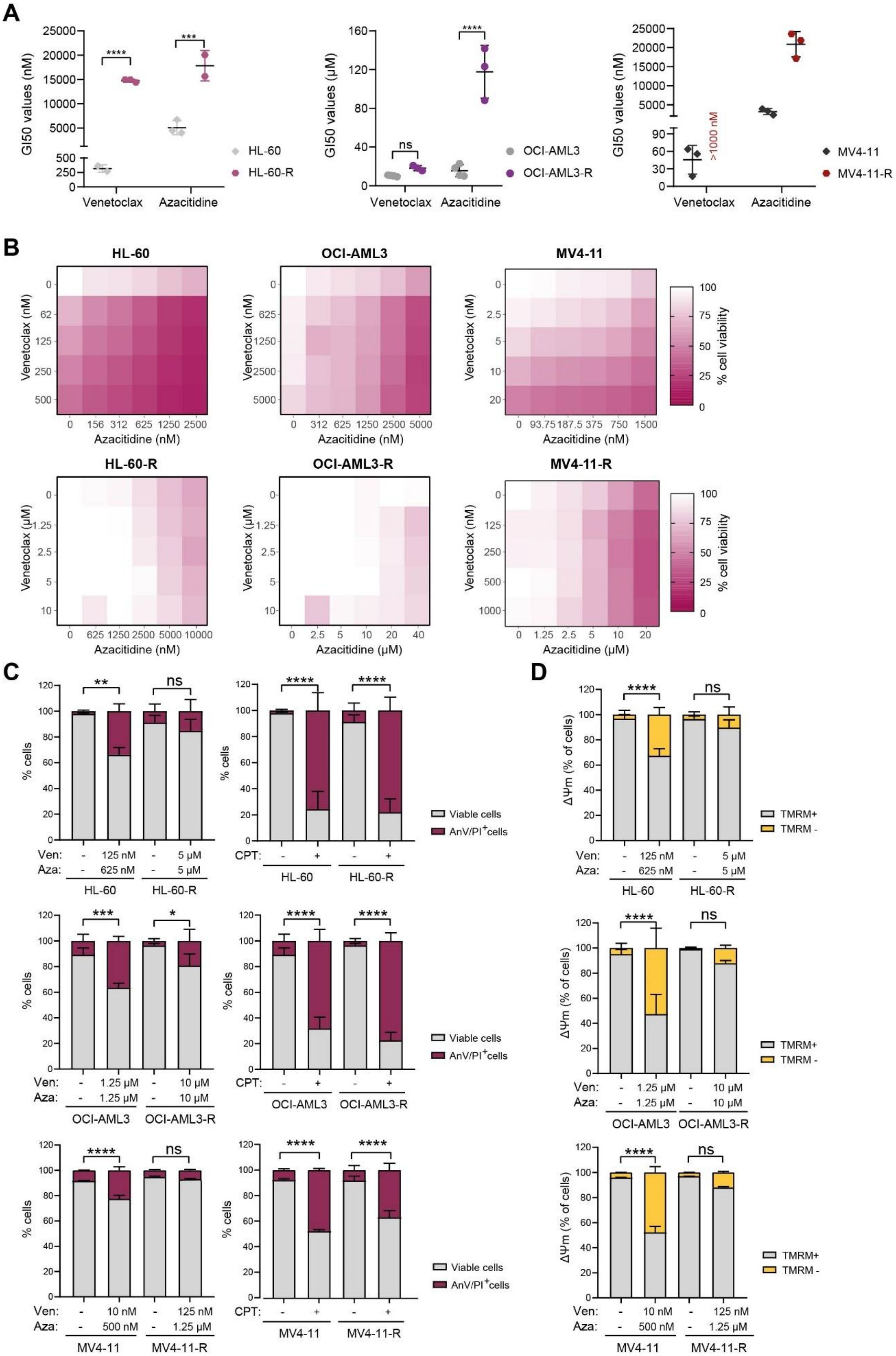

Supplemental Figure 7 (related to figure 3). (**A**) GI50 values for venetoclax and

azacitidine in HL-60, OCI-AML3, and MV4-11 parental and Ven-Aza-resistant cells. Data represent mean ± SD. Statistical significance was determined by unpaired t-student test analyses. (**B**) Heatmap representations of viability percentages of venetoclax and azacitidine combination at increasing concentrations (48 hours of treatment) in HL-60, OCI-AML3, and MV4-11 parental and Ven-Aza-resistant cell lines. (**C**) Percentages of viable and annexin V/propidium iodide positive (AnV/PI$^+$) HL-60, OCI-AML3, and MV4-11 parental and Ven-Aza-resistant cells after 48 hours upon Ven-Aza treatment and camptothecin (CPT) treatment (10 μM) (right graph). (**D**) Percentage of HL-60, OCI-AML3, and MV4-11 parental and Ven-Aza-resistant cell lines with mitochondrial membrane potential loss (Δψm) after 24 hours upon Ven-Aza treatment. Results obtained from 3 biological replicates. Data represent mean ± SEM and were analyzed by two-way analysis of variance (ANOVA) and Tukey post hoc test. **p<0.05, **p<0.01, ***p<0.001, ****p<0.0001,* ns, not statistically different.

Figure S8

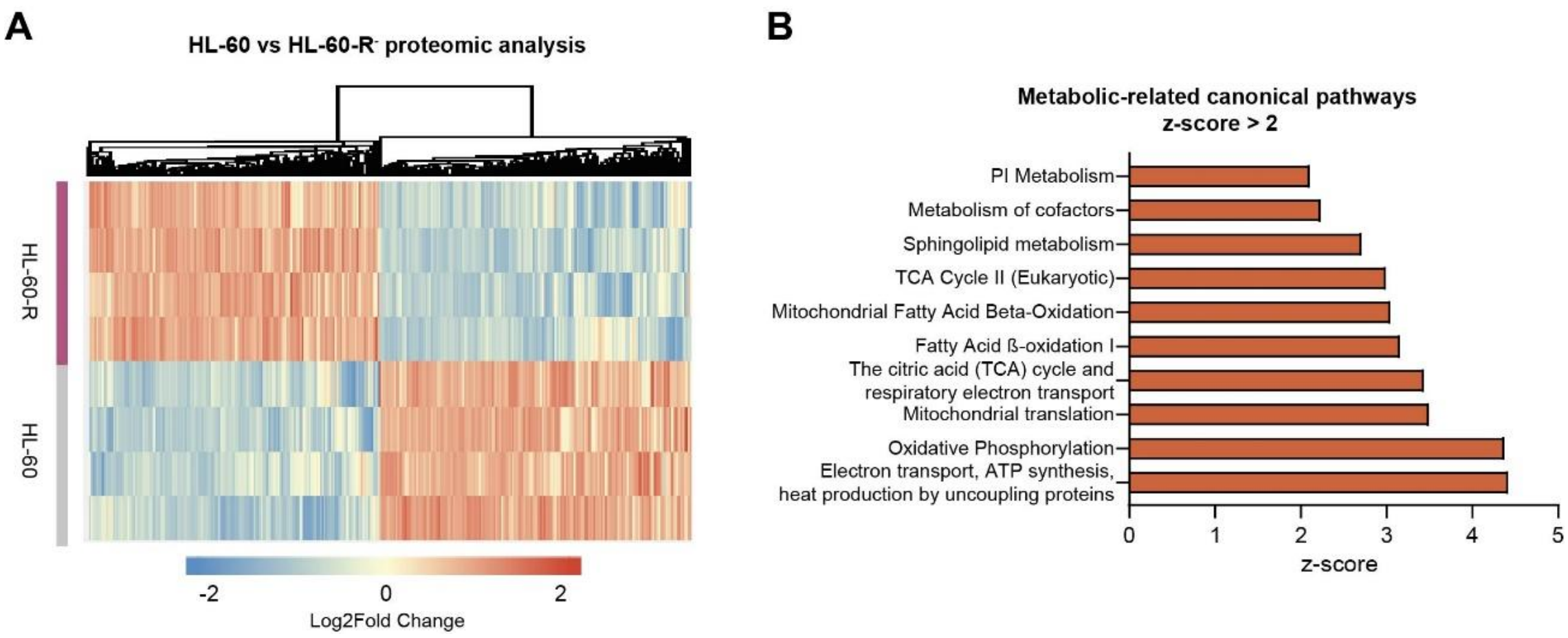

Supplemental Figure 8 (related to figure 3). (**A**) Heatmap representation of proteomics analyses: proteins differentially up-regulated (red) and down-regulated (blue) in HL-60-R compared with HL-60 cell line (n = 4 per cell line). (**B**) Bar graph represents z-score values of metabolism-related canonical pathways predicted to be up-regulated in HL-60-R compared with HL-60 parental. **p<0.05, **p<0.01, ***p<0.001, ****p<0.0001,* ns, not statistically different.

Figure S9

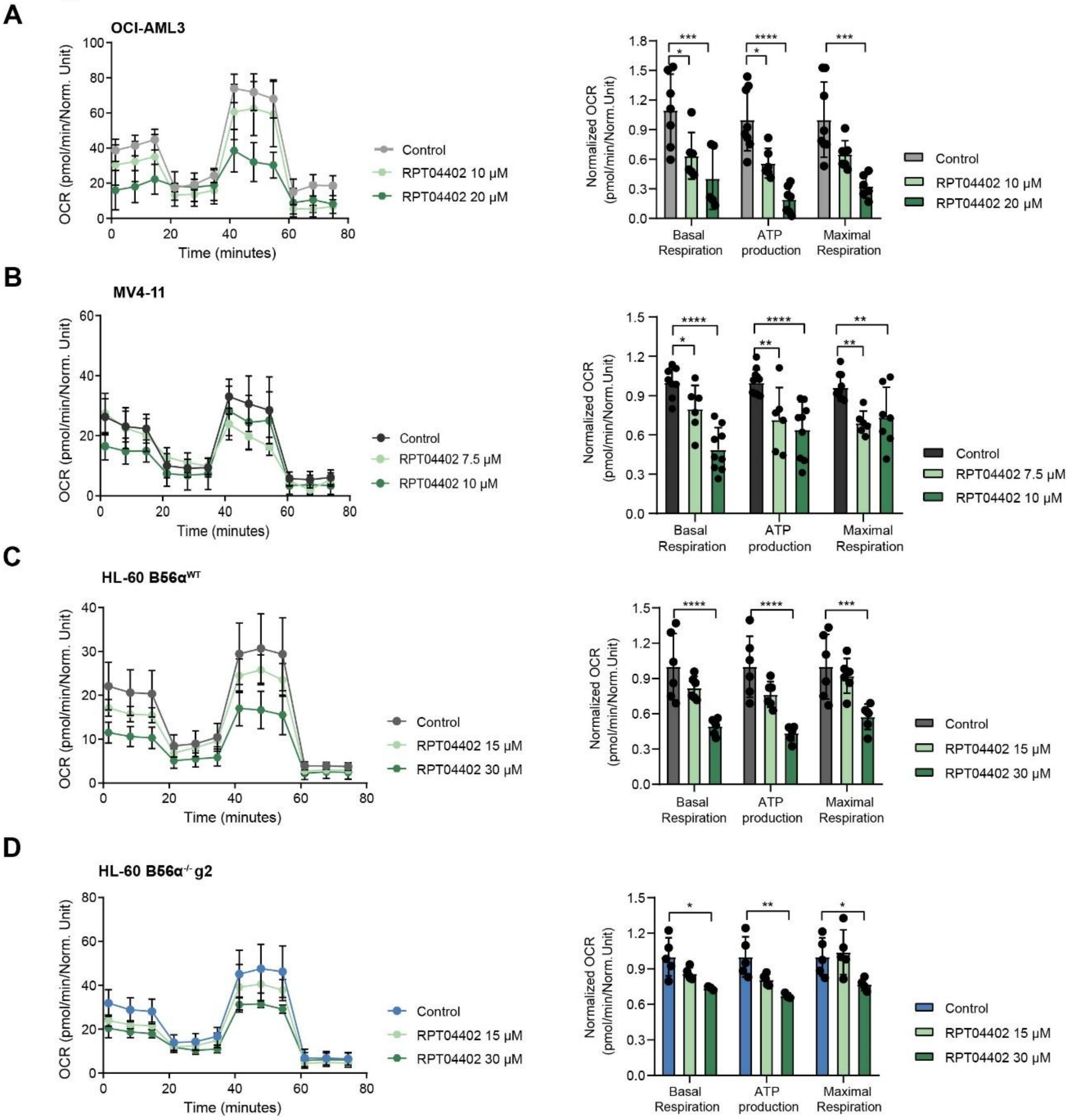

Supplemental Figure 9 (related to figure 4). Seahorse extracellular flux analysis shows dynamic representation of oxygen consumption rate (OCR) in parental (**A**) OCI-AML3, (**B**) MV4-11, (**C**) HL-60 B56α^{WT}, and (**D**) HL-60 B56α$^{-/-}$ g2 clone under basal conditions and treated with increasing concentrations of RPT04402 (12-hour treatment). Bar graphs show that RPT04402 treatment decreases basal respiration, ATP production, and maximal respiratory capacity of AML cell lines in a dose-dependent manner, expressed as a fold change of the control. Data represent mean ± SD. Statistical significance was determined by two-way analysis of variance (ANOVA) and Tukey post hoc test analyses *P < 0.05, **P < 0.01, ***P < 0.001, ****P < 0.0001, ns, not statistically different.

Figure S10

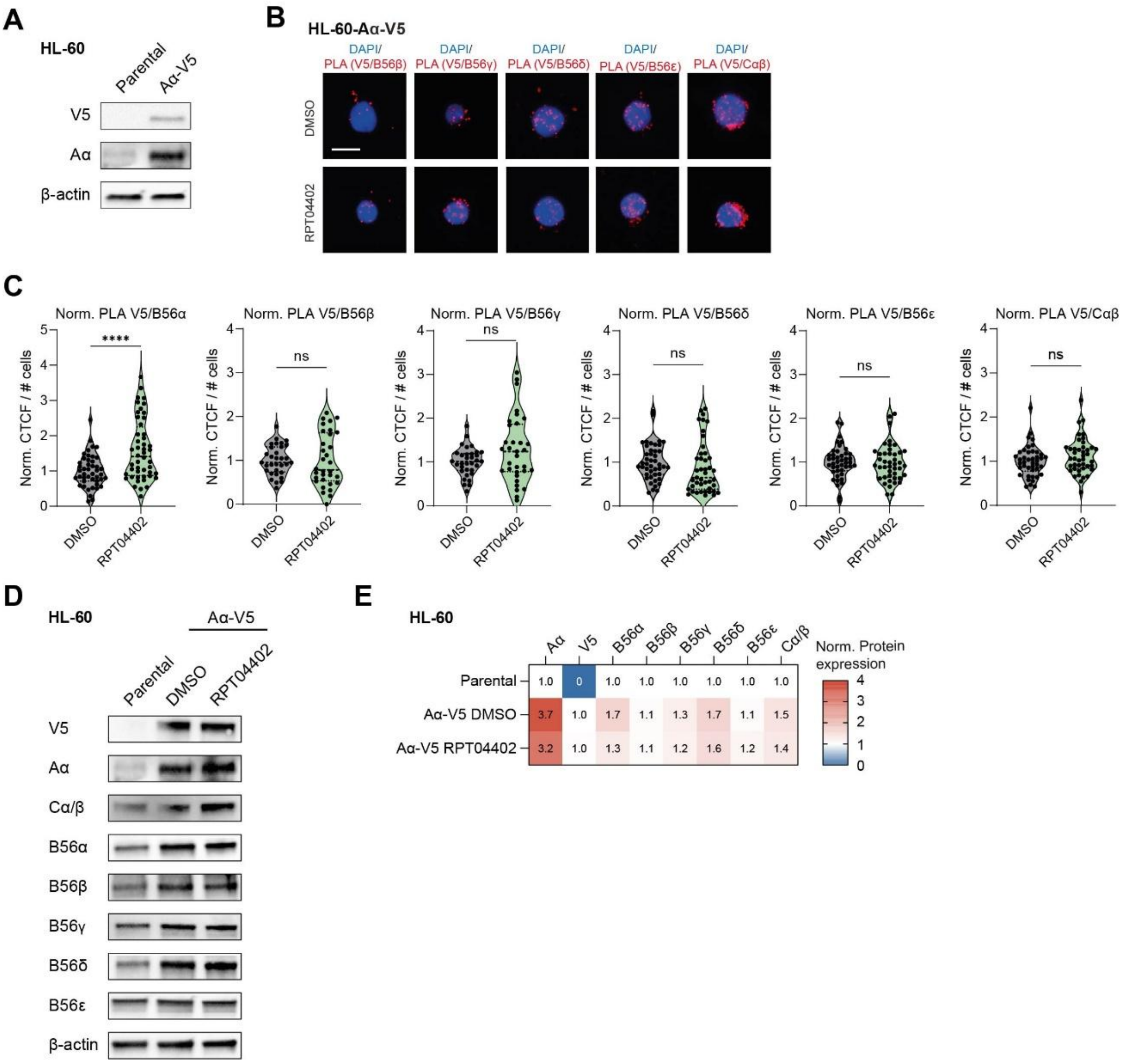

Supplemental Figure 10 (related to figure 4). (**A**) Graphical representation and western blot analysis of V5 tag protein and PP2A-Aα subunit after viral transduction of HL-60 cells. (**B**) Representative images of the proximity ligation analysis (PLA) targeting the interaction between Aα-V5 tagged and either B56β, B56γ, B56δ, B56ε, or Cα/β (Texas Red) in the absence or presence of RPT04402 (20 μM, 24 hours). Experiment performed on HL-60 cells transfected with Aα-V5. Nuclei stained with DAPI. All images 40x magnification, scale bar: 12.5 μm. N=3 biological replicates. (**C**) Relative quantification of PLA fluorescence signal from (B), measured by calculating the corrected total cell fluorescence (CTCF) of multiple images (7-10) per biological replicate (n=3). Statistical significance determined by one-way ANOVA and Tukey's post-test analysis. (**D**) Graphical representation and (**E**) protein quantification from western blot analyses in Aα-V5-transfected HL-60 cells of V5, PP2A-Aα, PP2A-C, and B56 family subunits after RPT04402 treatment. Protein expression levels are expressed as fold change of HL-60

parental cell line. β-actin was used as loading control for normalization of protein levels. **p<0.05, **p<0.01, ***p<0.001, ****p<0.0001*, ns, not statistically different.

Figure S11

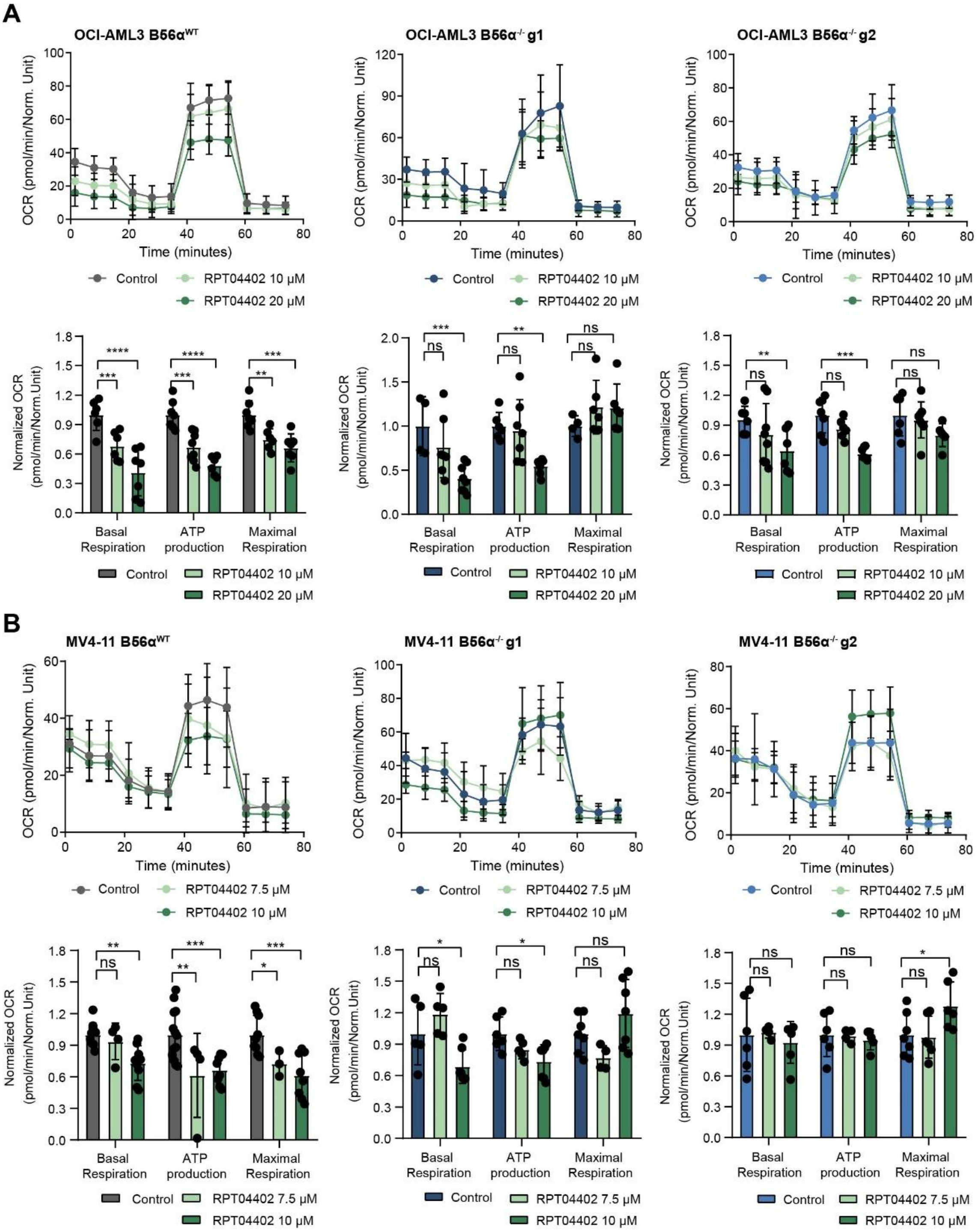

Supplemental Figure 11 (related to figure 4). Seahorse extracellular flux analysis shows dynamic representation of oxygen consumption rate (OCR) in (**A**) OCI-AML3 and (**B**) MV4-11 B56α^{WT} and B56α$^{-/-}$ clones under basal conditions and treated with increasing concentrations of RPT04402 (12-hour treatment). Bar graphs show that

RPT04402 treatment decreases basal respiration, ATP production, and maximal respiratory capacity of AML cell lines in a dose-dependent manner, expressed as a fold change of the control. Data represent mean ± SD. Statistical significance was determined by two-way analysis of variance (ANOVA) and Tukey post hoc test analyses *$p<0.05$, **$p<0.01$, ***$p<0.001$, ****$p<0.0001$*, ns, not statistically different.

Figure S12

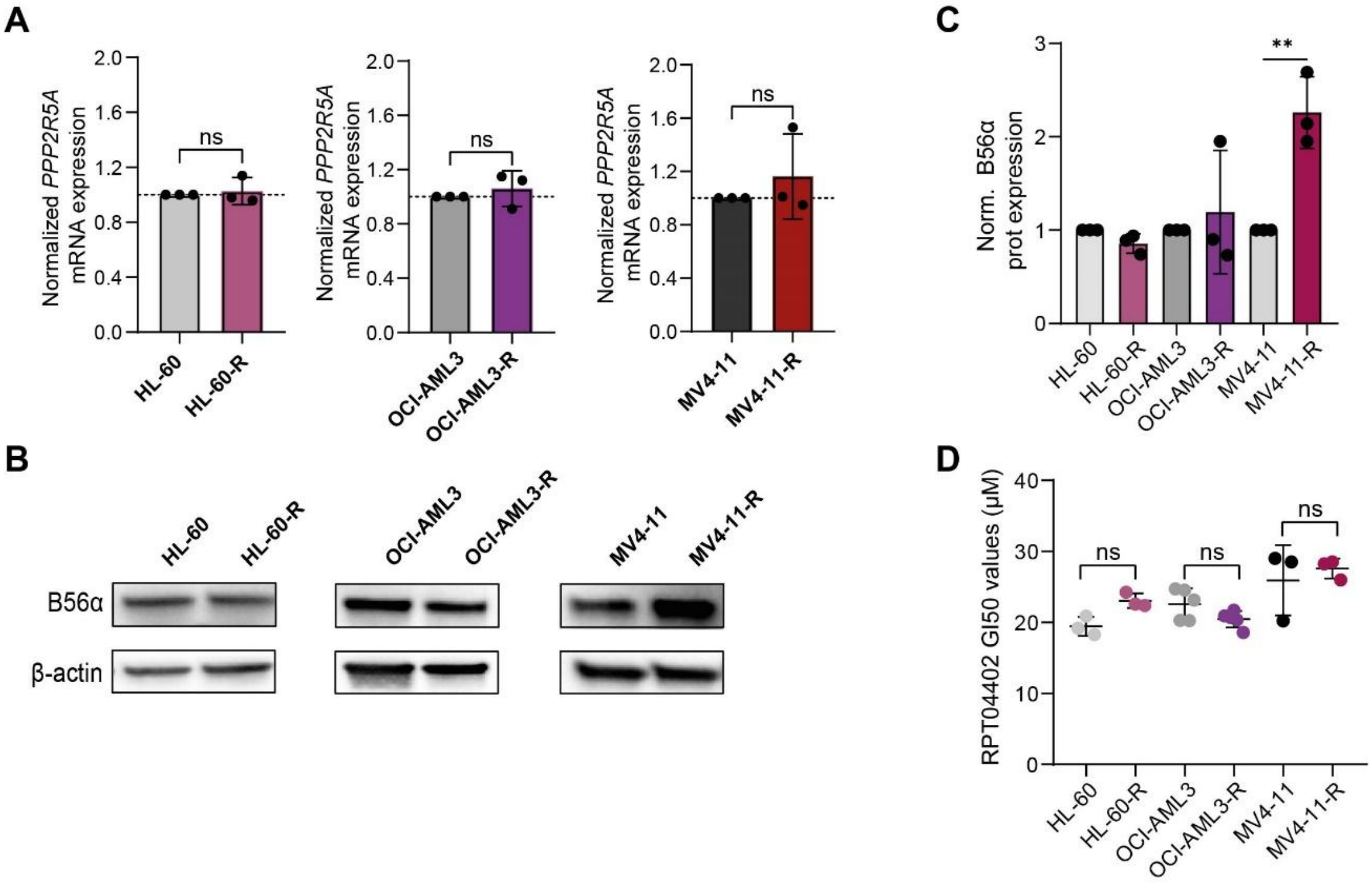

Supplemental Figure 12 (related to figure 4). (**A**) *PPP2R5A* expression levels measured by RT-qPCR in HL-60, OCI-AML3, and MV4-11 parental and Ven-Aza-resistant cells, expressed as a fold change of the parental cell lines. (**B**) Graphical representation and (**C**) normalized protein quantification from western blot analysis of B56α protein levels in HL-60, HL-60-R, OCI-AML3, OCI-AML3-R, MV4-11, and MV4-11-R cells expressed as the fold change of the parental cell lines. (**D**) GI50 values for RPT04402 in HL-60, OCI-AML3, and MV4-11 parental and Ven-Aza resistant cell lines. Results represent the average ± SD from 3 independent biological replicates. Statistical significance was determined by one-way analysis of variance (ANOVA) and Tukey post hoc test analyses *$p<0.05$, **$p<0.01$, ***$p<0.001$, ****$p<0.0001$*, ns, not statistically different.

Figure S13

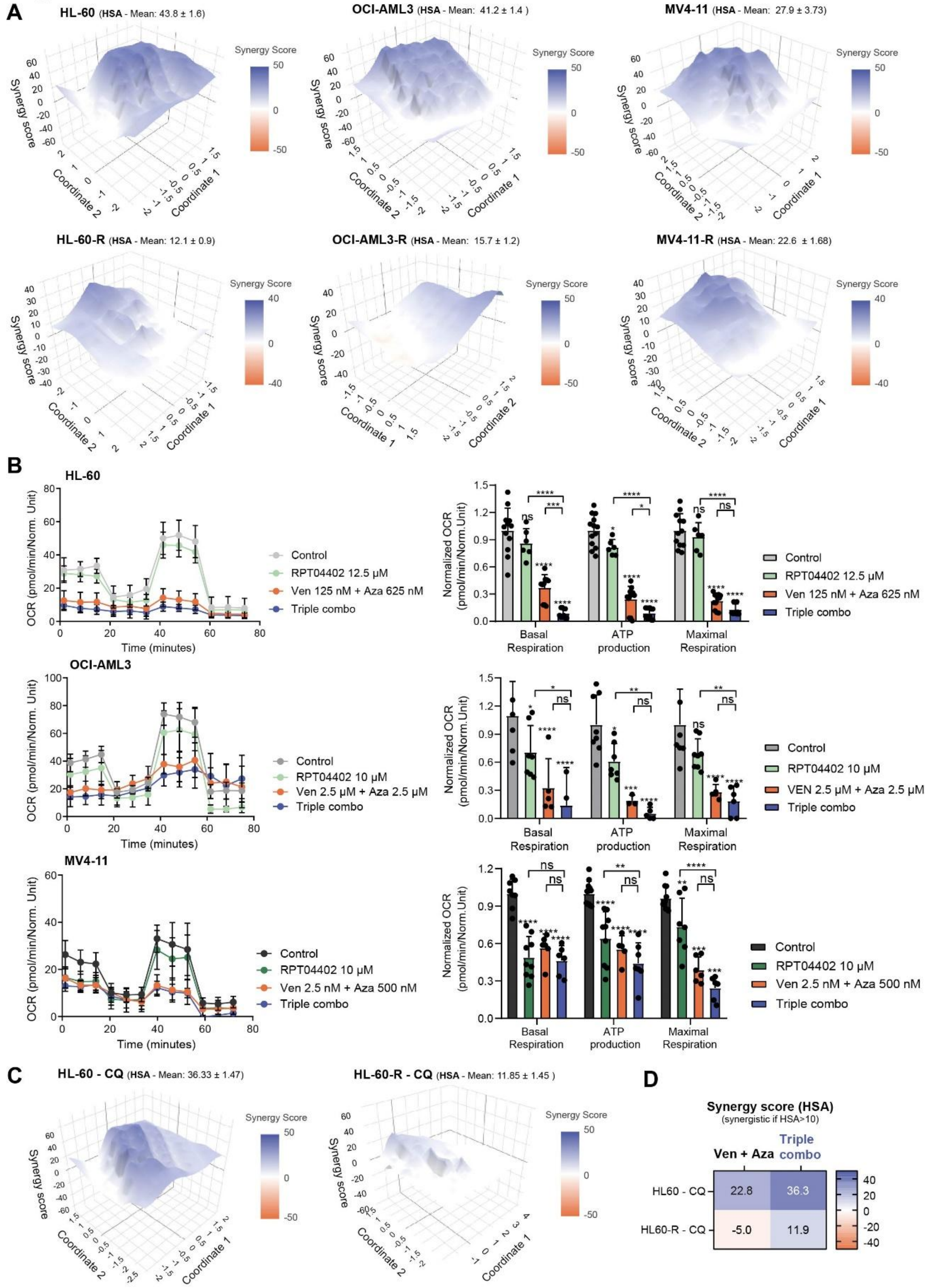

Supplemental Figure 13 (related to figure 5). (**A**) Highest single agent (HSA) synergy score (based on synergistic effect, blue color) maps for the triple therapy Ven-Aza-RPT04402 are shown for HL60, OCI-AML3, and MV4-11 parental and treatment-resistant cell lines. The two-dimensional coordinates of each three-drug dose

combination are determined by multidimensional scaling based on the similarity of their dose ranges, after which those maps are calculated (48-hour treatment). (**B**) Seahorse extracellular flux analysis shows dynamic representation of oxygen consumption rate (OCR) in HL-60, OCI-AML3, and MV4-11 cell lines at baseline (control) and after 12 hours of RPT04402, Ven-Aza, and Ven-Aza-RPT04402 (Triple combo) treatment. Bar graph represents basal respiration, ATP production, and maximal respiratory capacity levels expressed as fold change of the control. Both Ven-Aza and the triple combination therapy decrease OXPHOS-dependent energy production in Ven-Aza sensitive cell lines. RPT04402 concentrations for Triple combo treatment in HL-60, OCI-AML3, and MV4-11 cell lines were 12.5 μM for HL-60, 10 μM for OCI-AML3, and MV4-11 cells. Data represent mean ± SD. Statistical significance was determined by two-way analysis of variance (ANOVA) and Tukey post hoc test. (**C**) HSA synergy score (based on synergistic effect, blue color) maps for the triple therapy Ven-Aza-RPT04402 are shown for HL-60 and HL-60-R, calculated by CyQuant cell proliferation assay. (**D**) Heatmap representation of HSA synergy score means for Ven-Aza treatment and the therapy (Ven-Aza-RPT04402) in parental and Ven-Aza resistant HL60 cell lines (synergistic when HSA>10), calculated by CyQuant cell proliferation assay. Figures represent average results from 3 biological replicates. CQ, CyQuant. $*p<0.05$, $**p<0.01$, $***p<0.001$, $****p<0.0001$, ns, not statistically different.

Figure S14

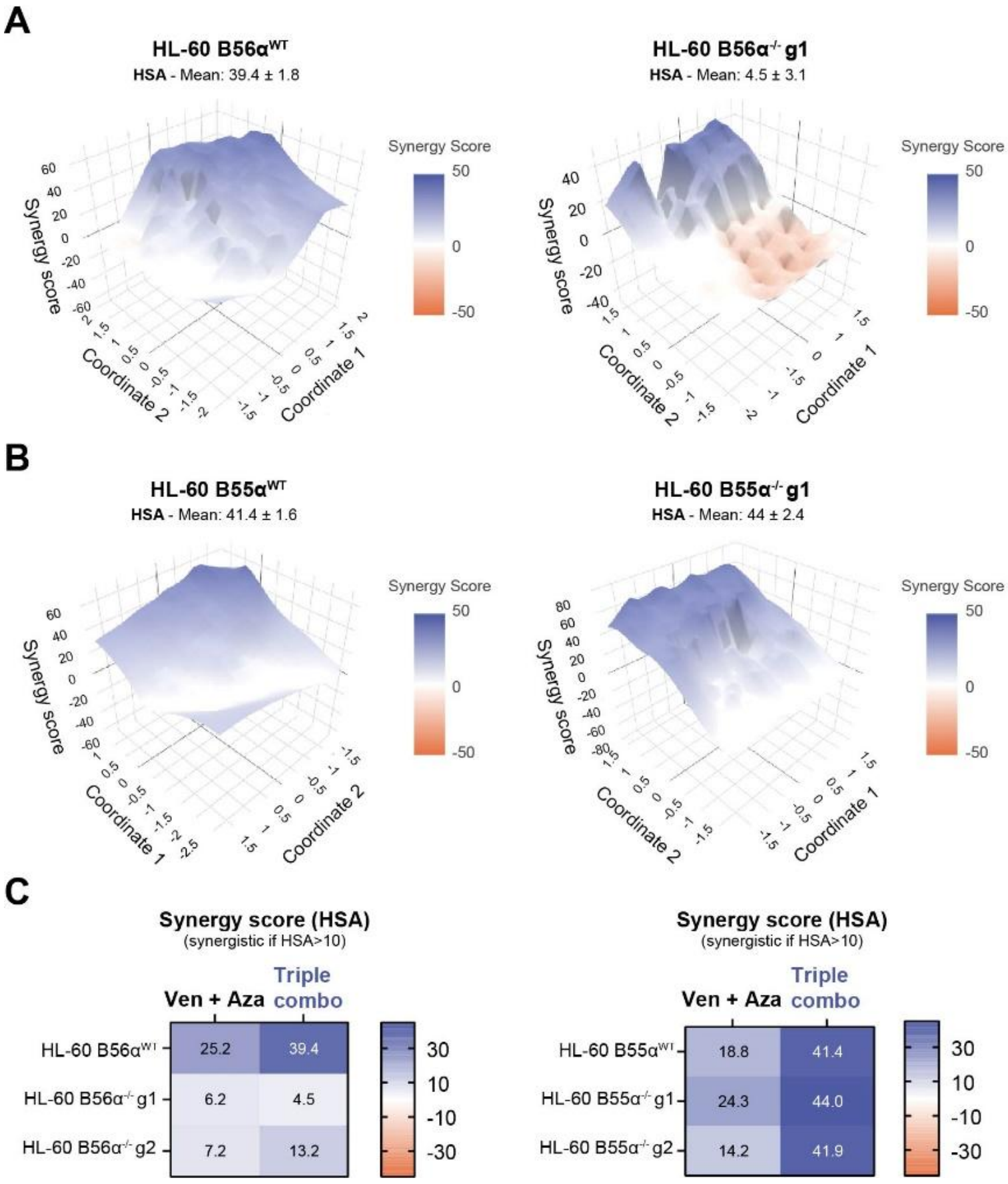

Supplemental Figure 14 (related to figure 5). (**A**) Highest single agent (HSA) synergy score (based on synergistic effect, blue color) maps for the triple therapy Ven-Aza-RPT04402 are shown for HL-60 B56α and (**B**) B55α CRISPR-Cas9 clones. The two-dimensional coordinates of each three-drug dose combination are determined by multidimensional scaling based on the similarity of their dose ranges, after which those maps are calculated (48-hour treatment). (**C**) Heatmap representation of HSA synergy score means of Ven-Aza and Ven-Aza-RPT04402 treatment in HL-60 B56α and B55α CRISPR clones. All heatmaps represent average results from 3 independent biological replicates. **p<0.05, **p<0.01, ***p<0.001, ****p<0.0001*.

Figure S15

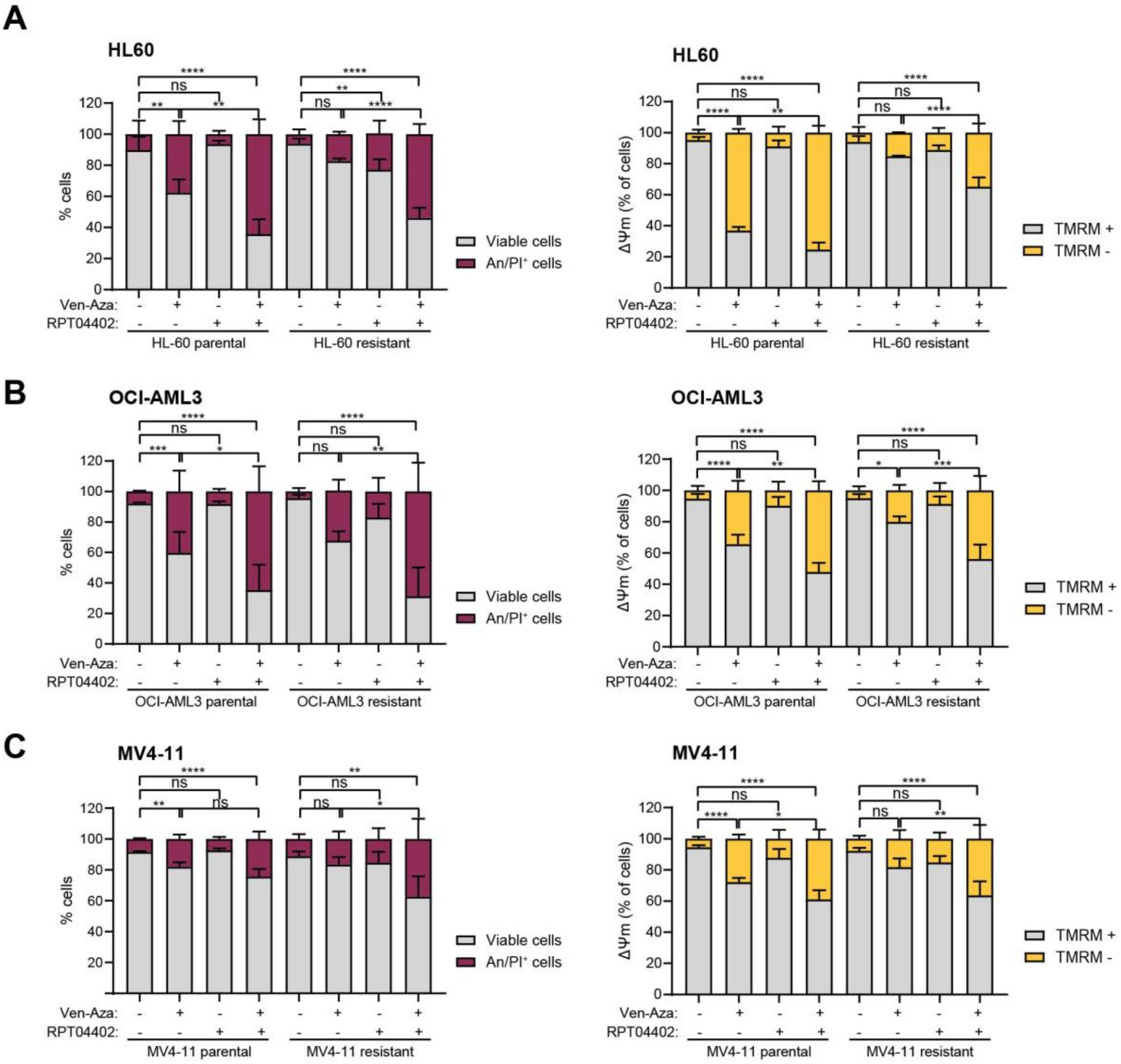

Supplemental Figure 15 (related to figure 5). On the left, percentages of viable and annexin V/propidium iodide positive (AnV/PI$^+$) (**A**) HL-60, (**B**) OCI-AML3, and (**C**) MV4-11 parental and Ven-Aza-resistant cells after 48 hours upon RPT04402, and/or Ven-Aza treatment. On the right, percentage of HL-60, OCI-AML3, and MV4-11 parental and Ven-Aza-resistant cell lines with mitochondrial membrane potential loss (Δψm) after 24 hours upon RPT04402, and/or Ven-Aza treatment. Results obtained from 3 biological replicates. Data represent mean ± SEM and were analyzed by two-way analysis of variance (ANOVA) and Tukey post hoc test. *p<0.05, **p<0.01, ***p<0.001, ****p<0.0001, ns, not statistically different.

Figure S16

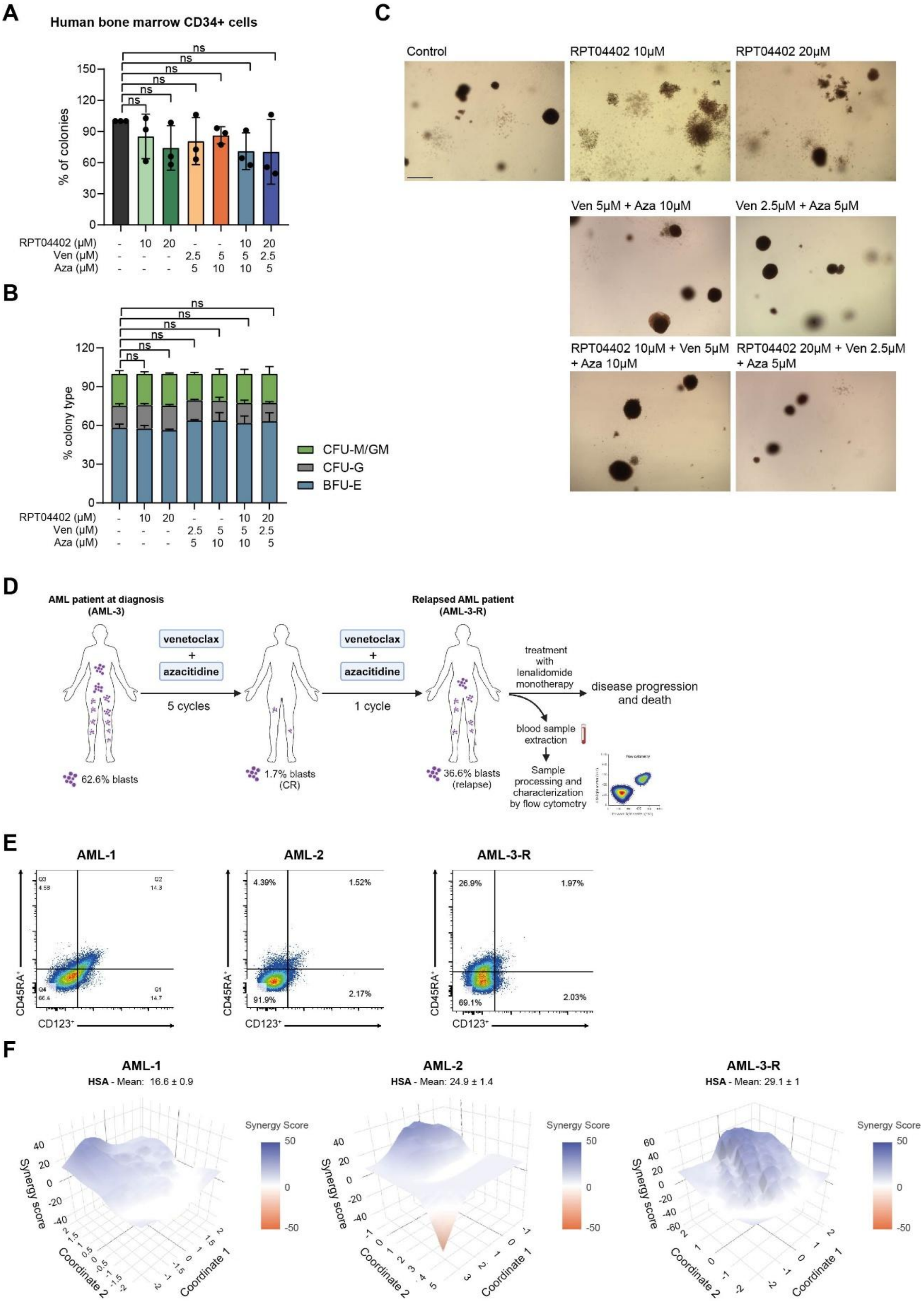

Supplemental Figure 16 (related to figure 6). Bar graphs show the percentage (%) of (**A**) colonies and (**B**) type of colonies (CFU-M/GM, CFU-G, and BFU-E) grown from normal human bone marrow-derived CD34⁺ cells upon treatment with RPT04402 (10

and 20 μM), double (venetoclax 2.5-5 μM and azacitidine 5-10 μM) or triple combination therapies after 12-14 days. Data represent mean ± SD. Statistical significance was determined by one-way analysis of variance (ANOVA) and Dunnett's post hoc test. (% of colonies) and two-way ANOVA test followed by Dunnett's test (colony type). (**C**) Representative images of normal human bone marrow-derived $CD34^+$ colonies grown upon treatment exposure. Scale bars: 500 μm. (**D**) Graphical summary of the treatment scheme followed for AML-3-R patient. At diagnosis, AML patient presented 62.6% of BM blasts and started treatment with venetoclax-azacitidine regimen. After 5 cycles of treatment, the patient achieved CR (BM blast <5%), but relapsed one cycle afterwards (presence of BM blasts 36.6%). Patient received lenalidomide monotherapy, but rapidly progressed and died. Blood sample was obtained at relapse, processed, and characterized by flow cytometry for future experiments. Figure created in Biorender.com. (**E**) Flow cytometry characterization of AML-1, AML-2, and AML-3-R primary samples of CD45RA/CD123 phenotype. (**F**) HSA synergy score maps for the triple therapy Ven-Aza-RPT04402 are shown for 3 AML primary samples (AML-1, AML-2, AML-3-R) (60-hour treatment) obtained from ~ 2 biological replicates. The two-dimensional coordinates of each three-drug dose combination are determined by multidimensional scaling based on the similarity of their dose ranges, after which those maps are calculated.

References

1. Peris I, et al. Activation of the PP2A-B56α heterocomplex synergizes with venetoclax therapies in AML through BCL2 and MCL1 modulation. *Blood*. 2023;141(9):1047-1059.

2. Subramanian A, et al. Gene set enrichment analysis: A knowledge-based approach for interpreting genome-wide expression profiles. Published online 2005.

3. Liberzon A, et al. The Molecular Signatures Database Hallmark Gene Set Collection. *Cell Syst*. 2015;1(6):417-425. doi:10.1016/j.cels.2015.12.004

4. Mootha VK, et al. PGC-1α-responsive genes involved in oxidative phosphorylation are coordinately downregulated in human diabetes. *Nat Genet*. 2003;34(3):267-273.

5. Bottomly D, et al. Integrative analysis of drug response and clinical outcome in acute myeloid leukemia. *Cancer Cell*. 2022;40(8):850-864. doi:10.1016/j.ccell.2022.07.002

6. Lachén-Montes M, et al. Involvement of Glucosamine 6 Phosphate Isomerase 2 (GNPDA2) Overproduction in β-Amyloid- and Tau P301L-Driven Pathomechanisms. *Biomolecules*. 2024;14(4):394. doi:10.3390/biom14040394

7. Tyanova S, et al. The Perseus computational platform for comprehensive analysis of (prote)omics data. *Nat Methods*. 2016;13(9):731-740. doi:10.1038/nmeth.3901

8. Zhou Y, et al. Metascape provides a biologist-oriented resource for the analysis of systems-level datasets. *Nat Commun*. 2019;10(1):1-10. doi:10.1038/s41467-019-09234-6

9. Krämer A, et al. Causal analysis approaches in ingenuity pathway analysis. *Bioinformatics*. 2014;30(4):523-530. doi:10.1093/bioinformatics/btt703

10. Heberle H, et al. InteractiVenn: A web-based tool for the analysis of sets through Venn diagrams. *BMC Bioinformatics*. 2015;16(1):1-7. doi:10.1186/s12859-015-0611-3
