# Supplementary material for: Stabilization of the PP2A-B56α complex overcomes venetoclax-azacitidine resistance by impairing OXPHOS in AML
Source: JCI Insight. 2026 Sep 15;11(20):e205218. doi: 10.1172/jci.insight.205218 (PMC13726563; doi:10.1172/jci.insight.205218)

Full unedited gel for Figure 1C

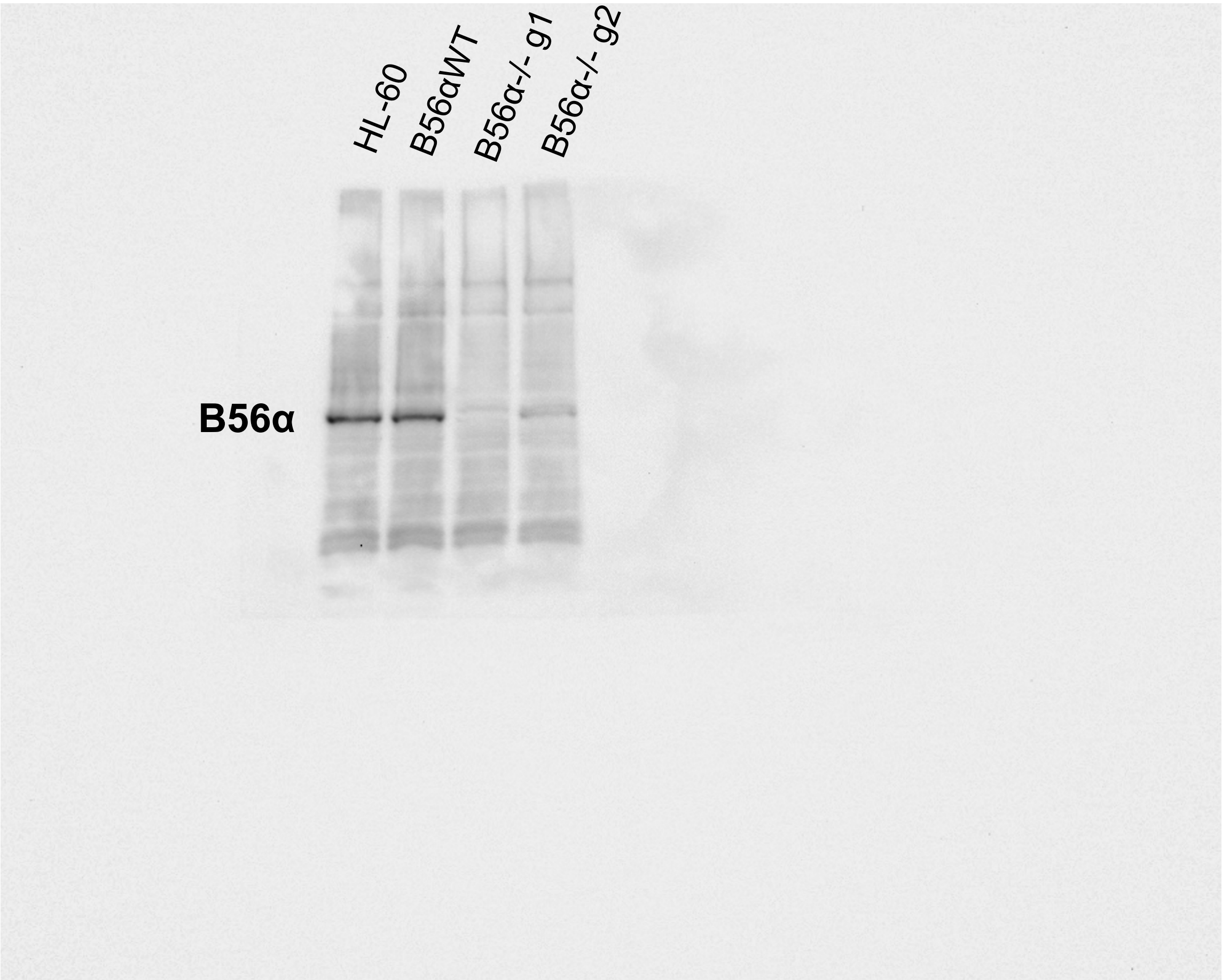

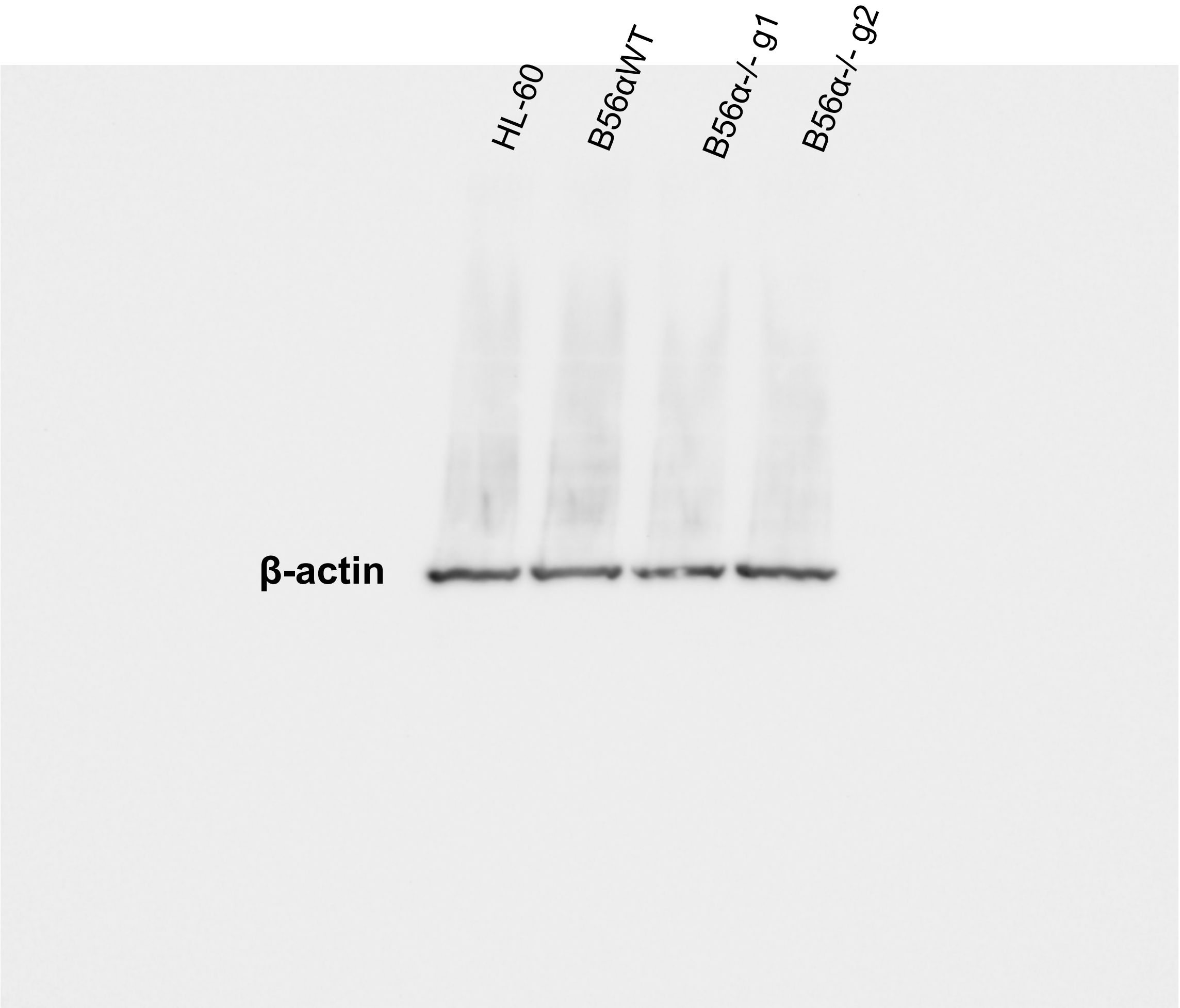

Full unedited gel for Figure 1F

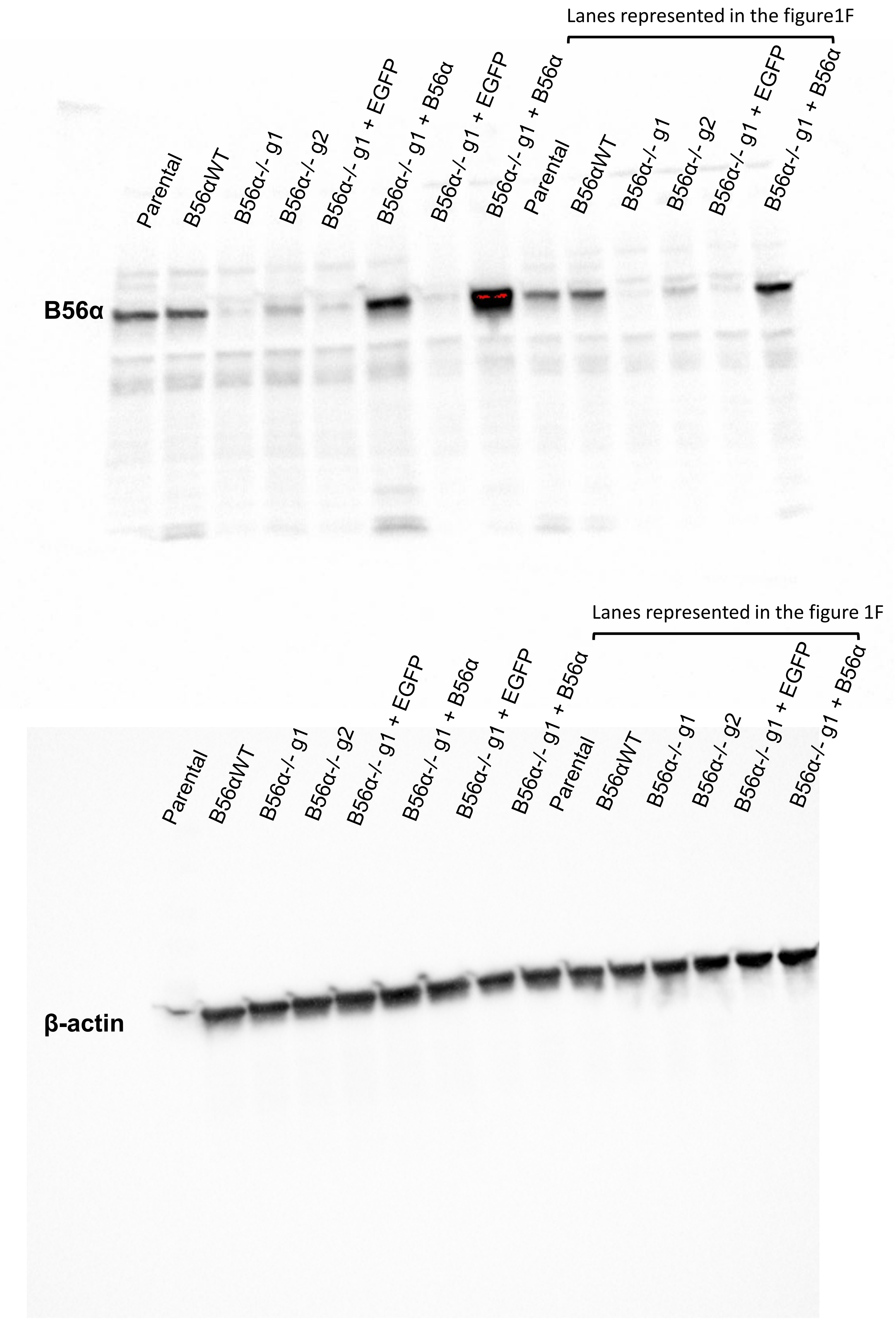

Full unedited gel for Figure S2A

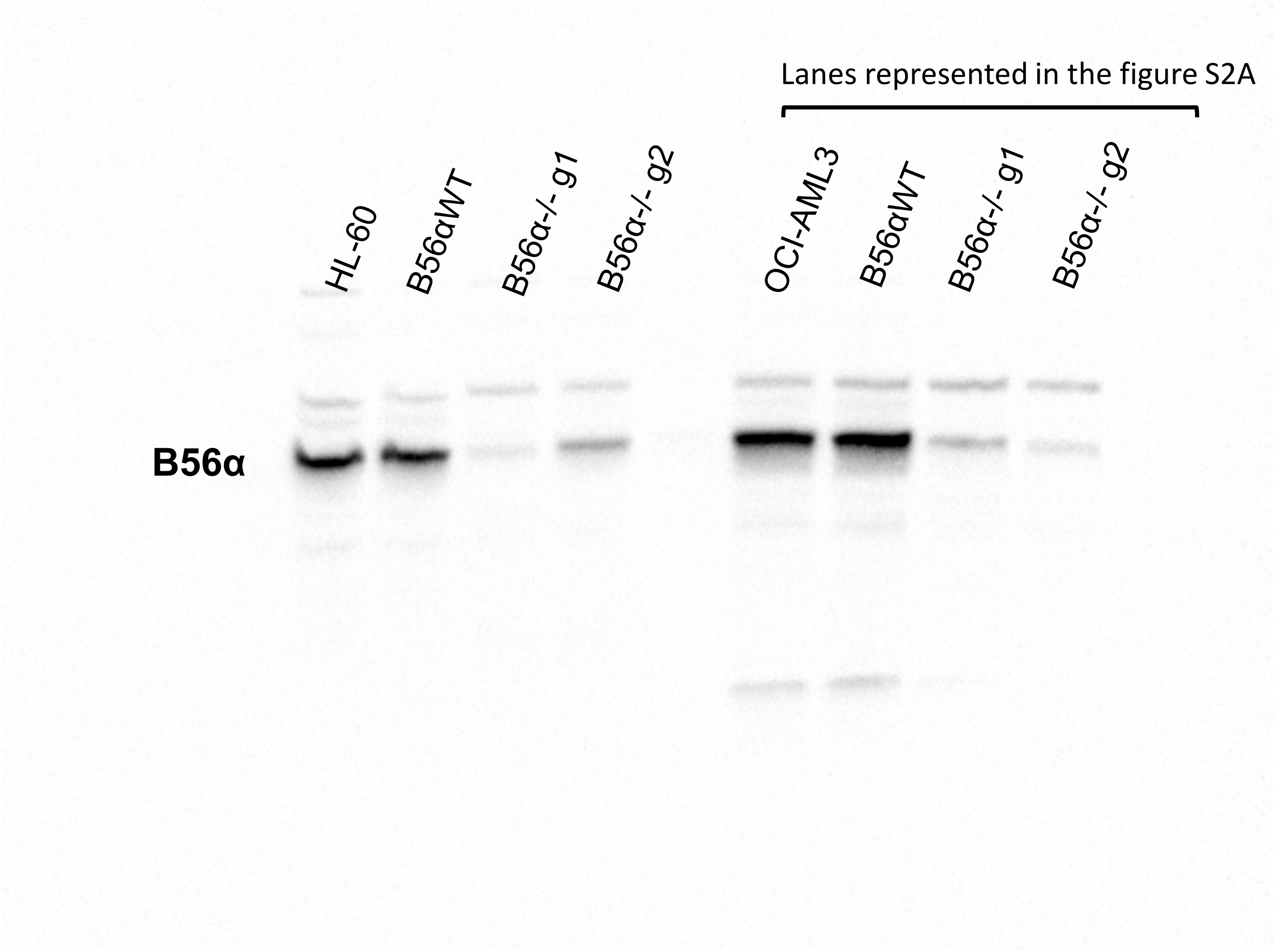

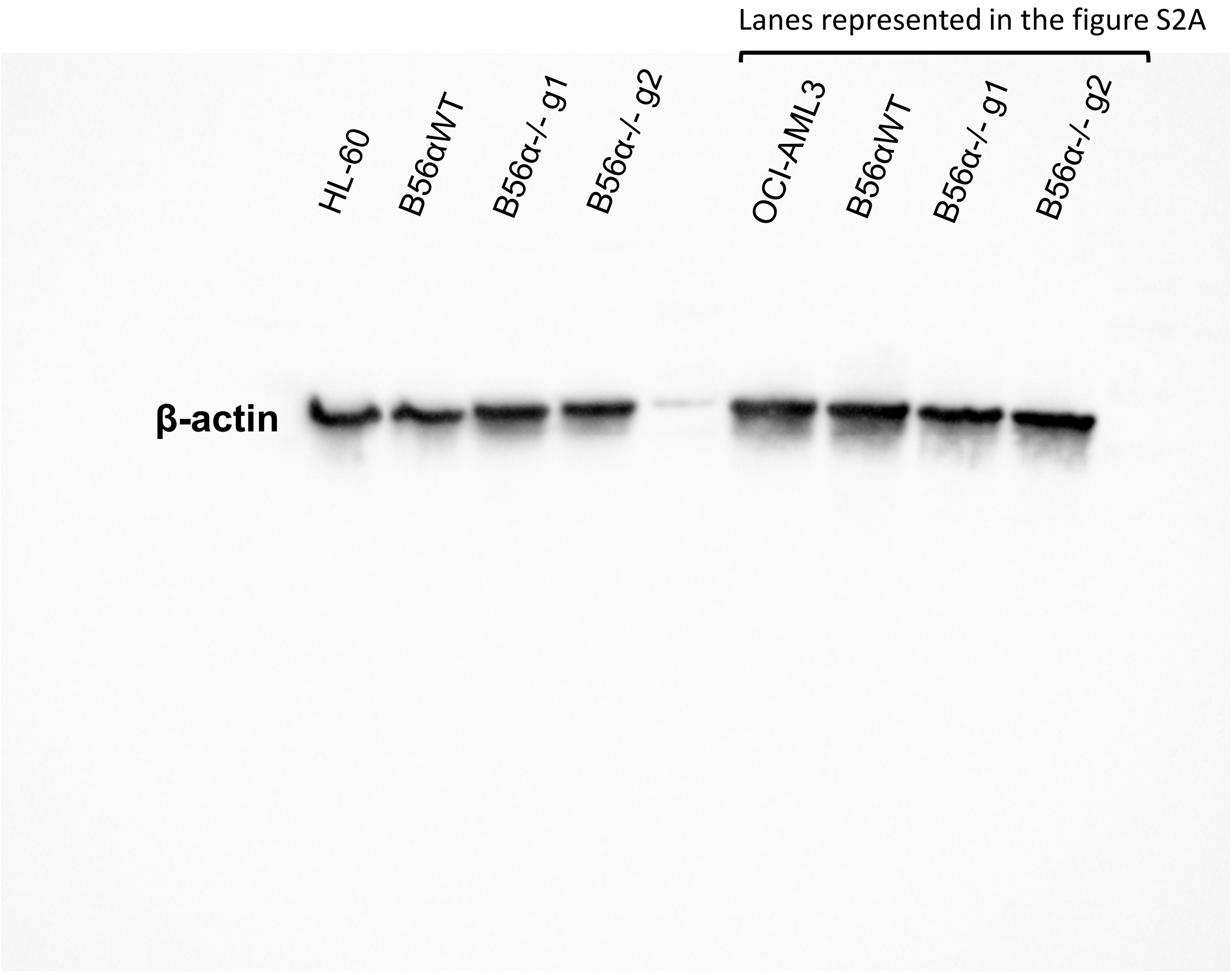

Full unedited gel for Figure S2A

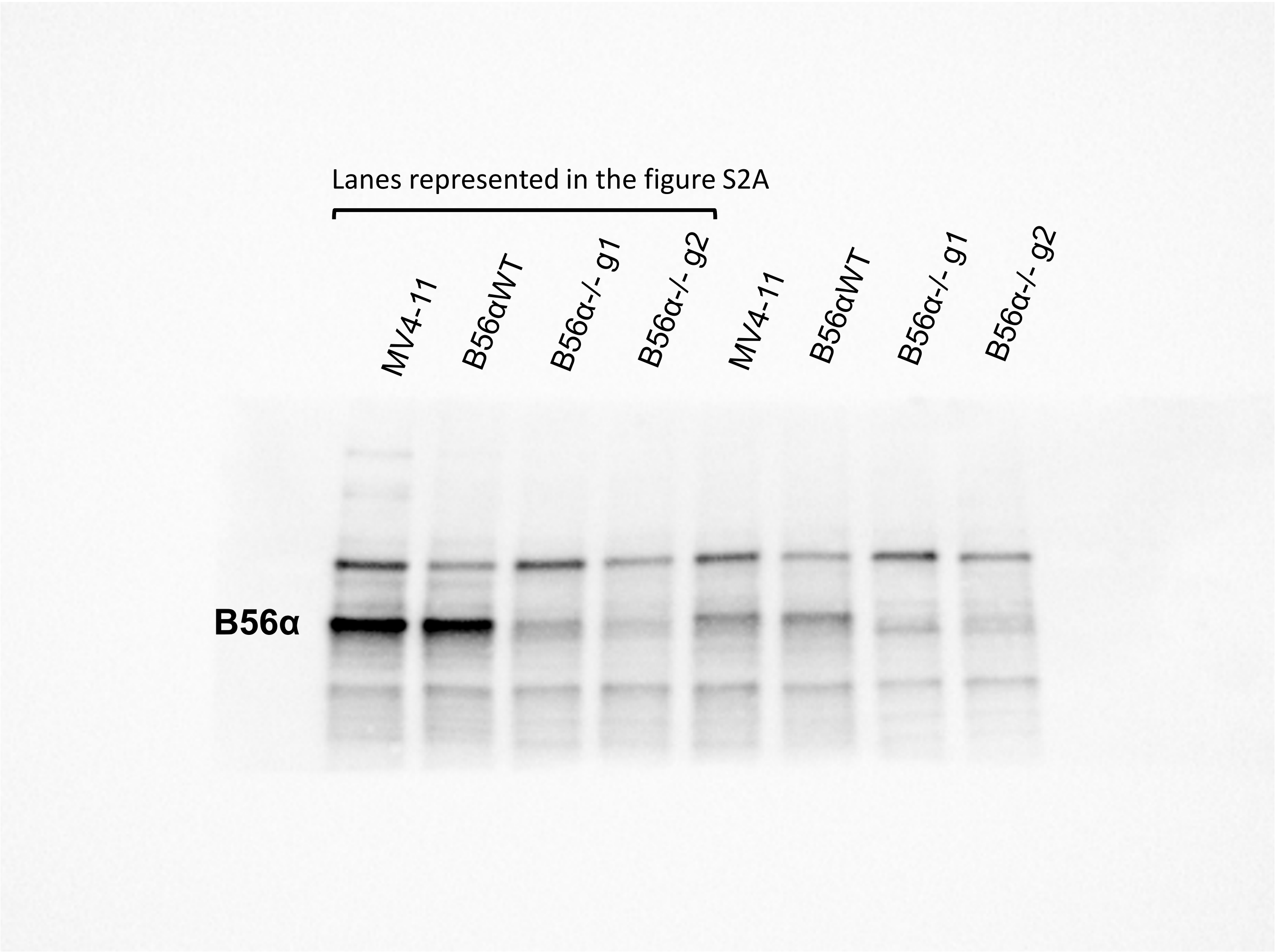

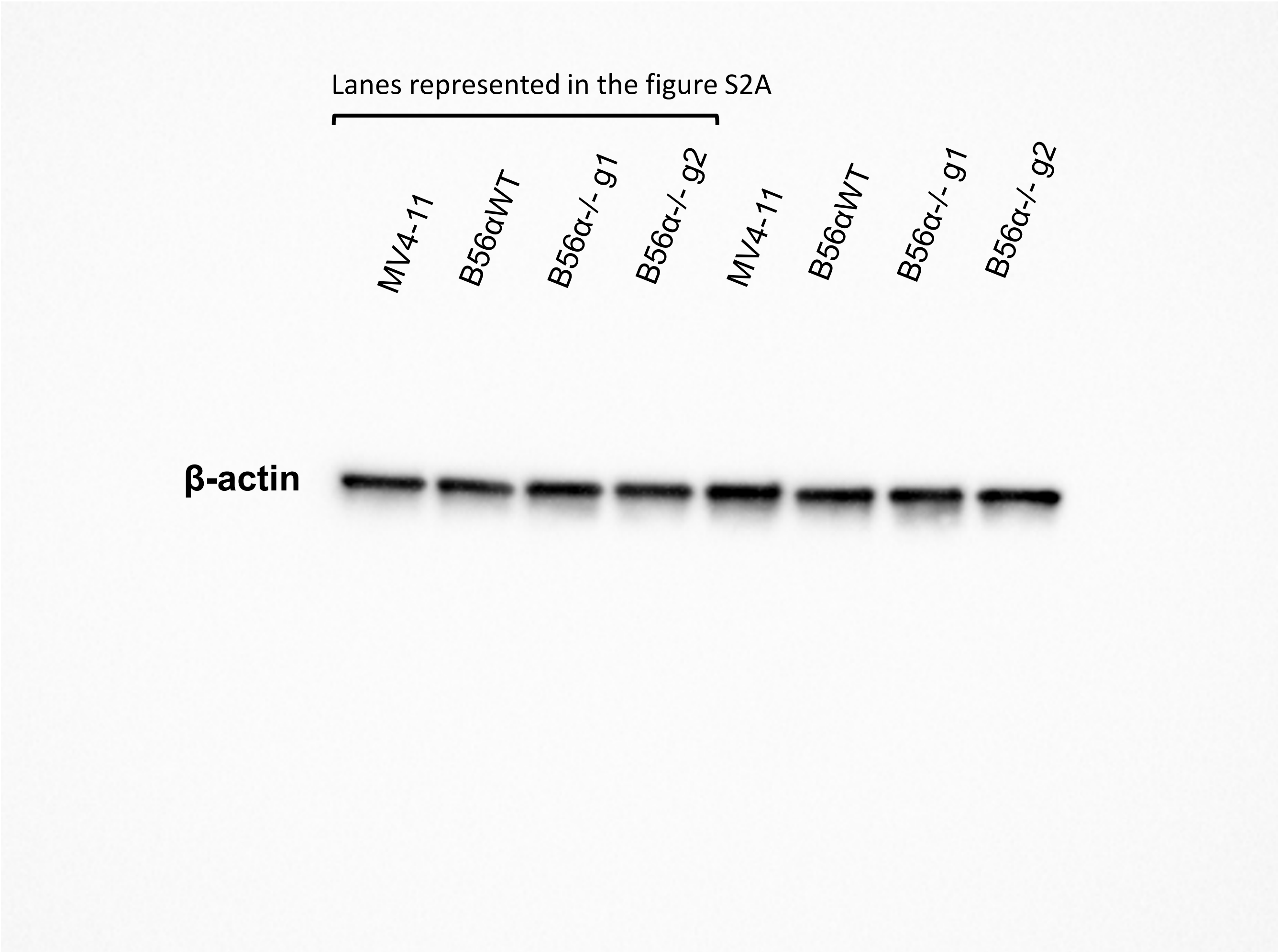

Full unedited gel for Figure S4A

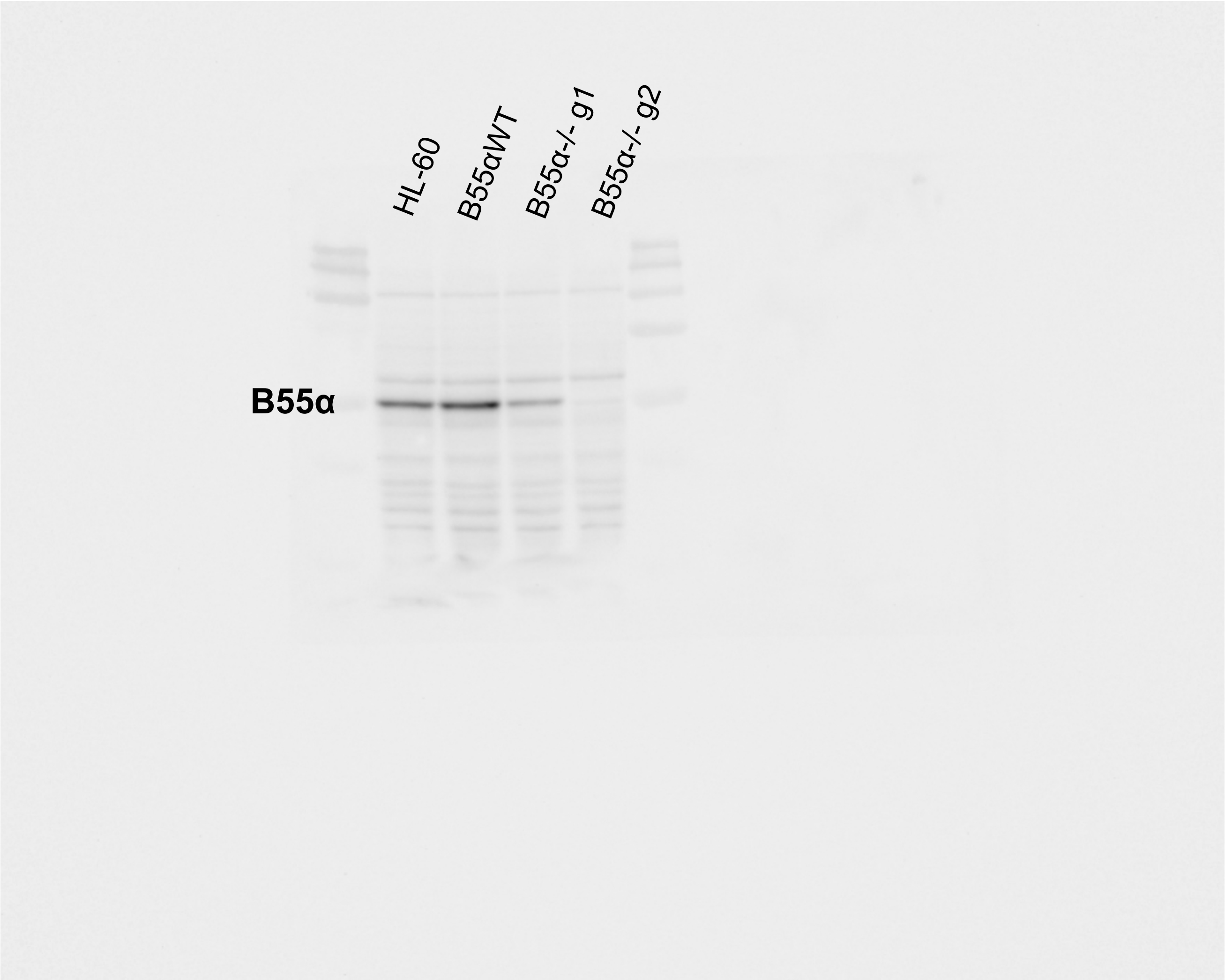

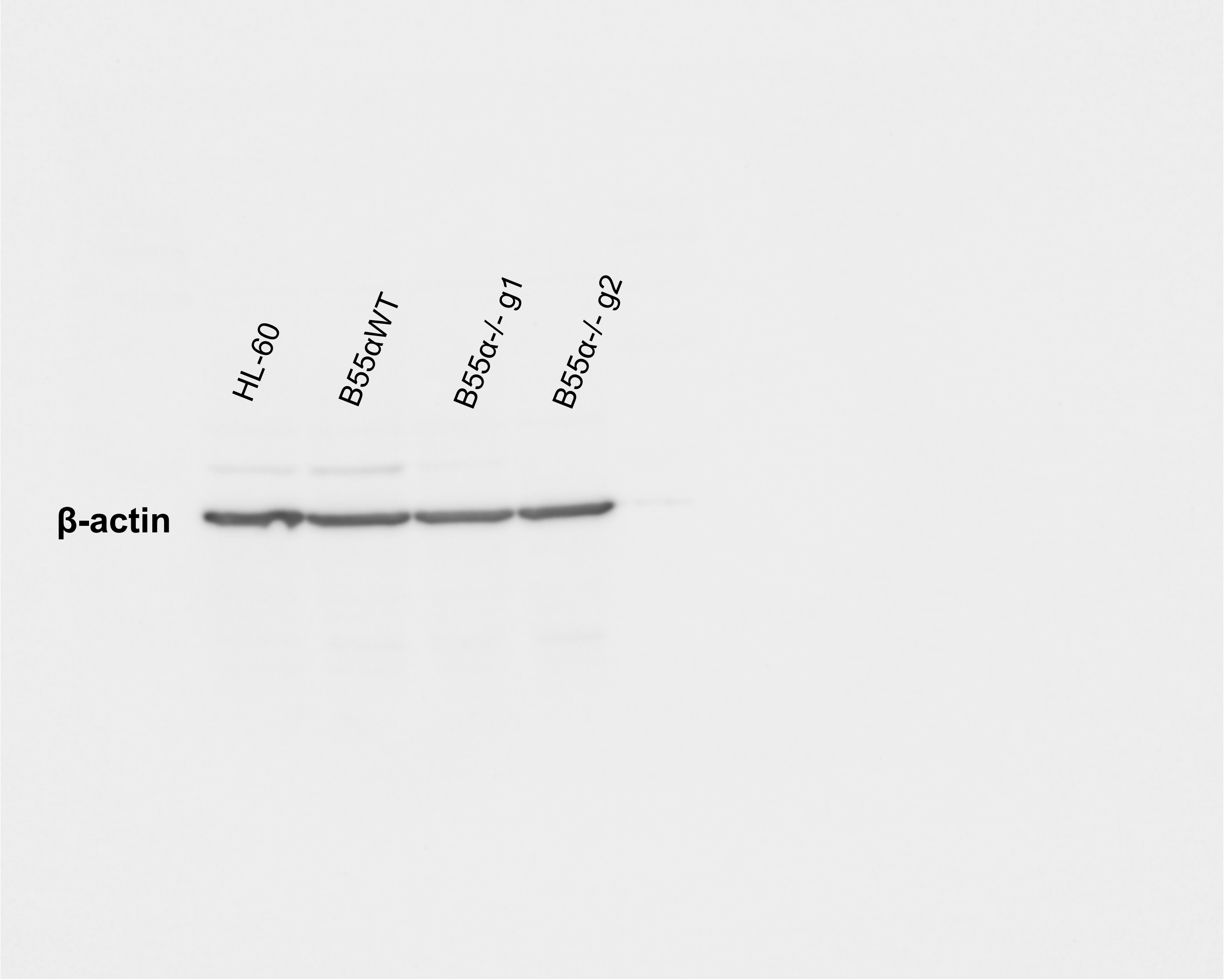

Full unedited gel for Figure S10A

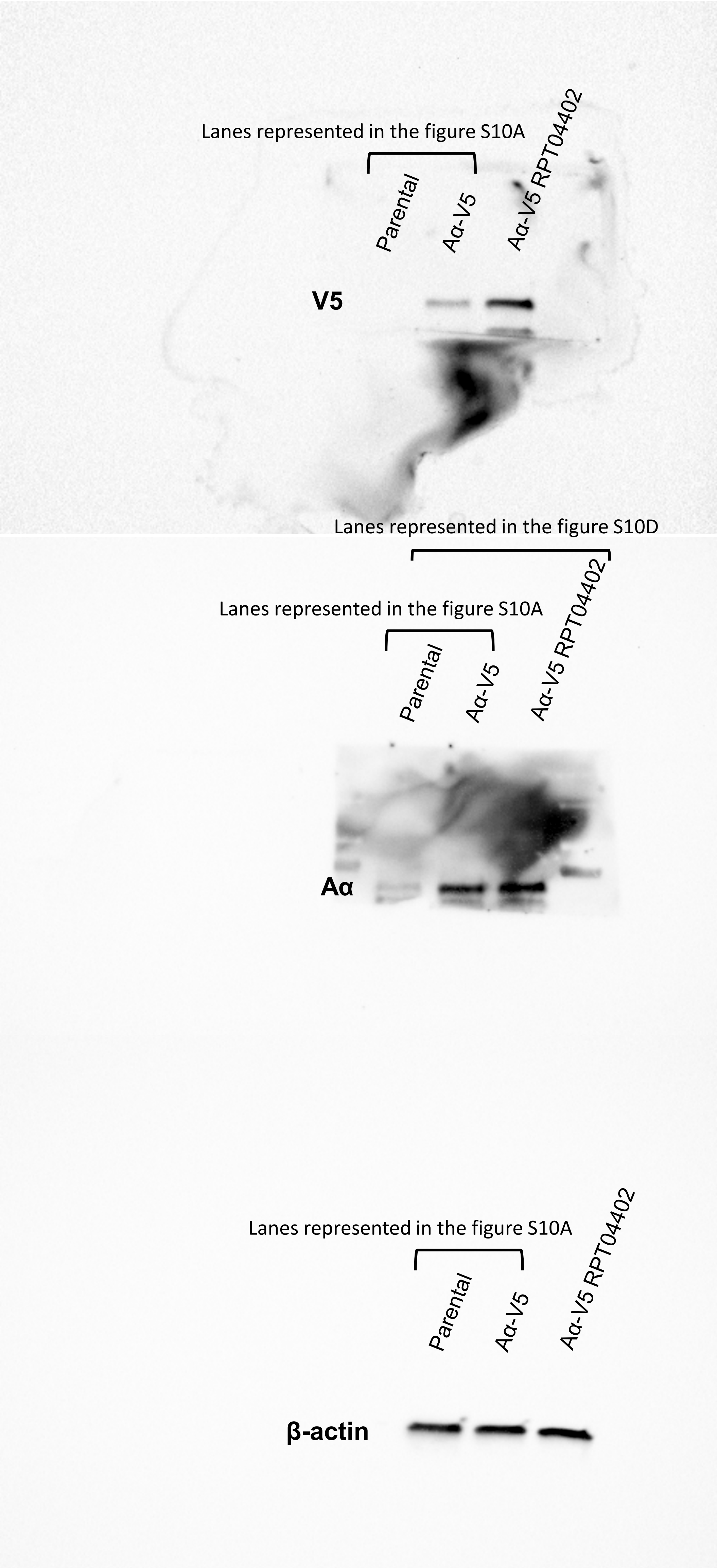

Full unedited gel for Figure S10D

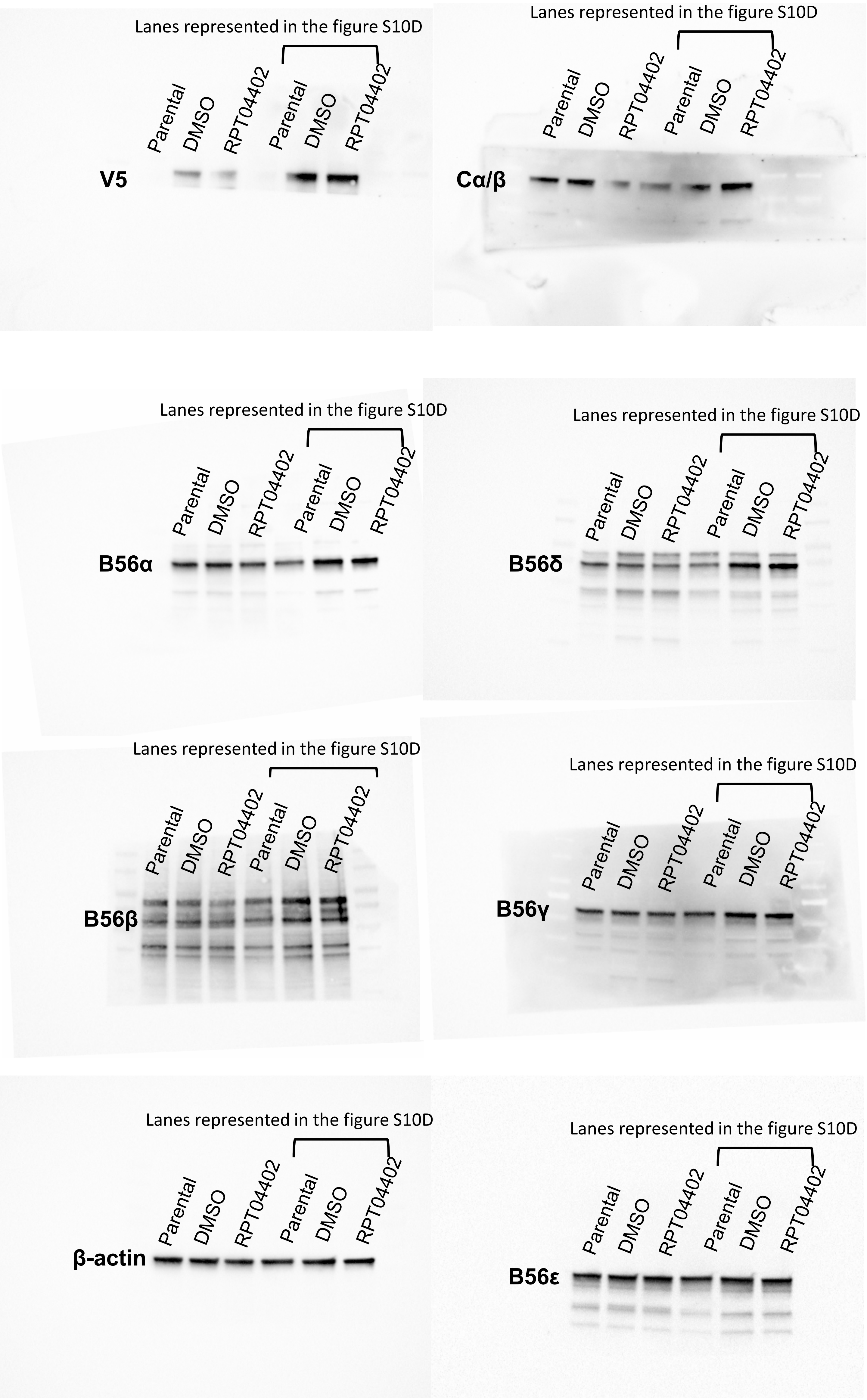

Full unedited gel for Figure S12B

Lanes represented in the figure S12B

Lanes represented in the figure S12B

HL-60 Hl-60-R HL-60 Hl-60-R HL-60 Hl-60-R OCI-AML3 OCI-AML3-R

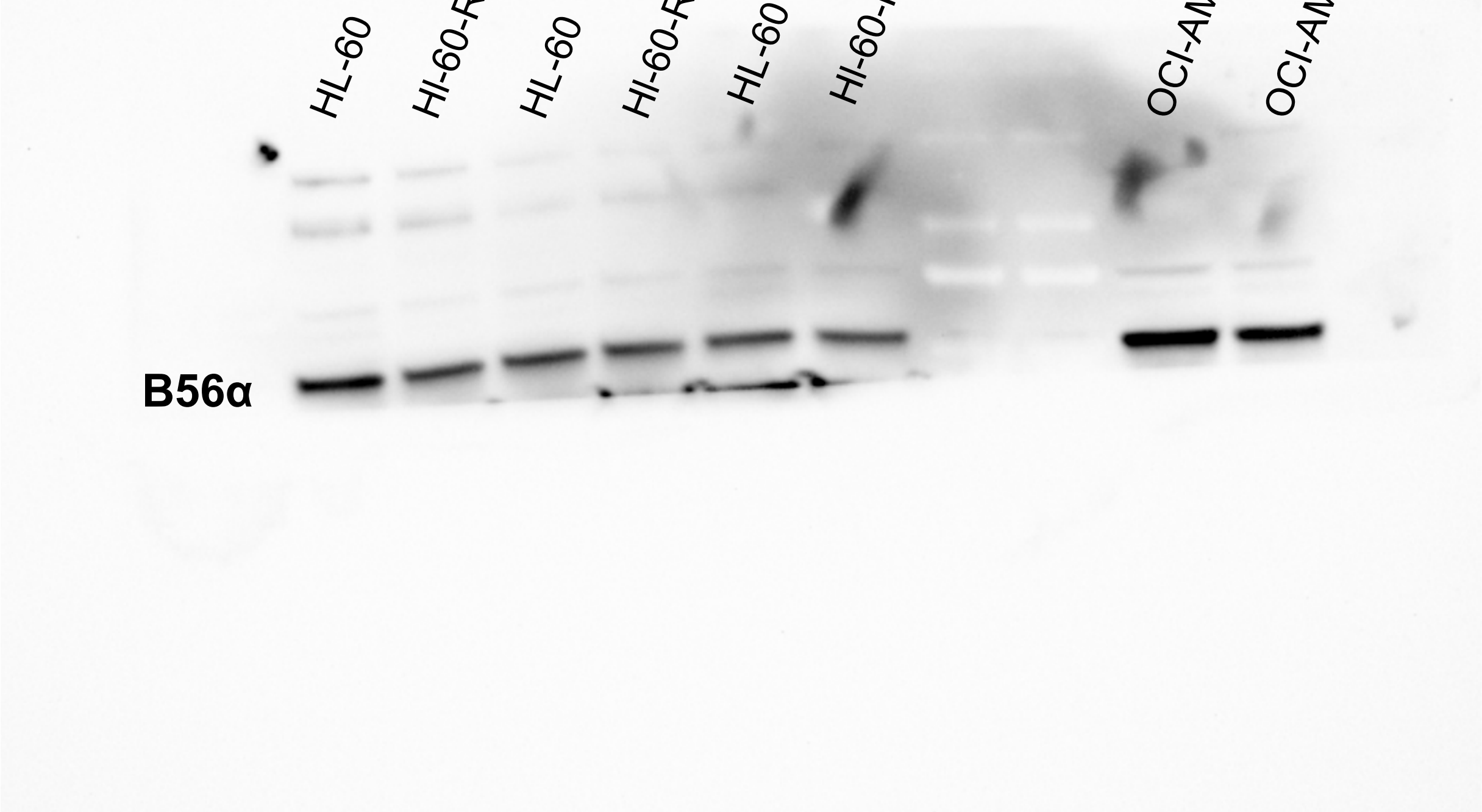

B56α

Lanes represented in the figure S12B

Lanes represented in the figure S12B

HL-60 Hl-60-R HL-60 Hl-60-R HL-60 Hl-60-R OCI-AML3 OCI-AML3-R

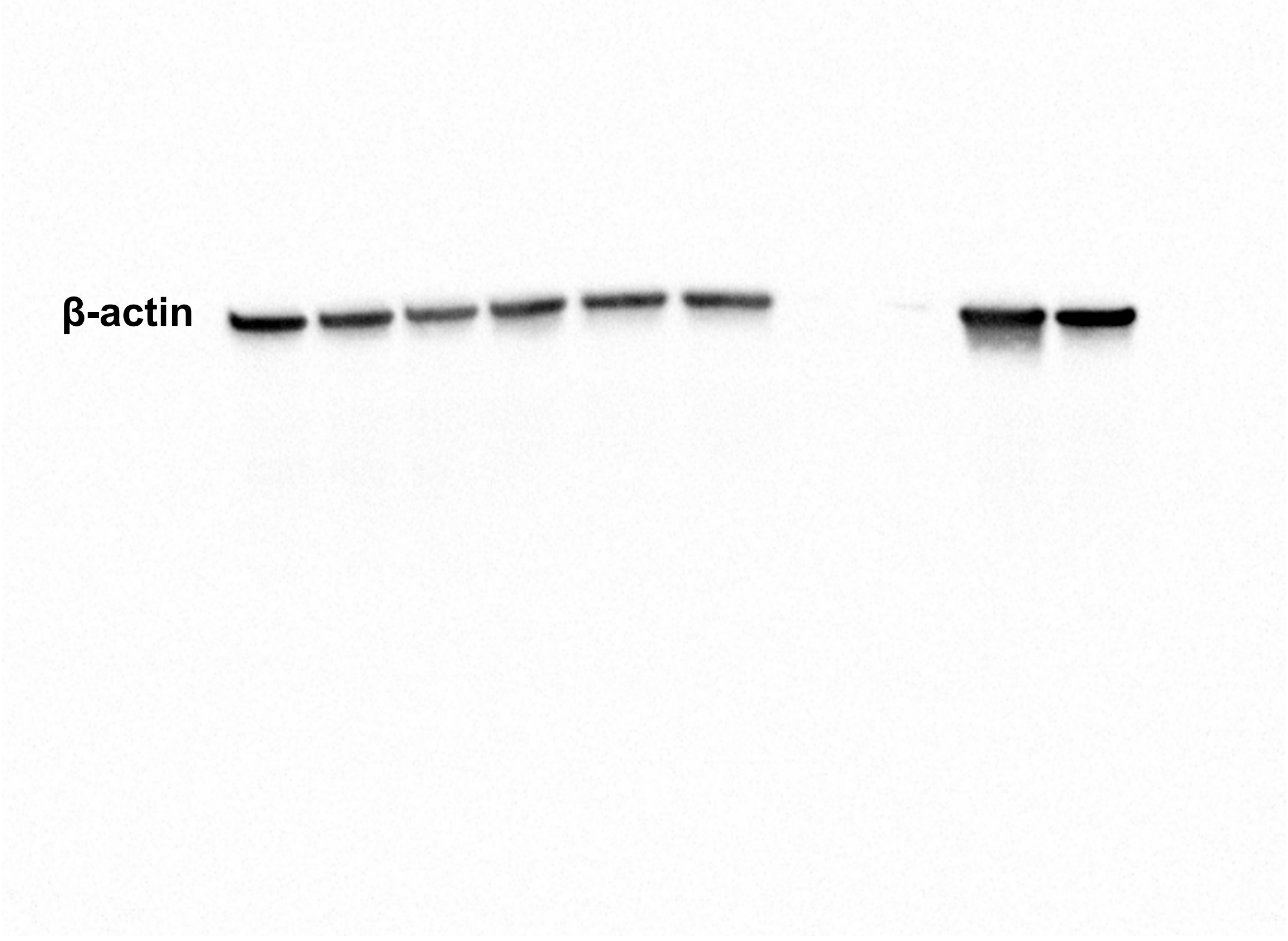

β-actin

Full unedited gel for Figure S12B

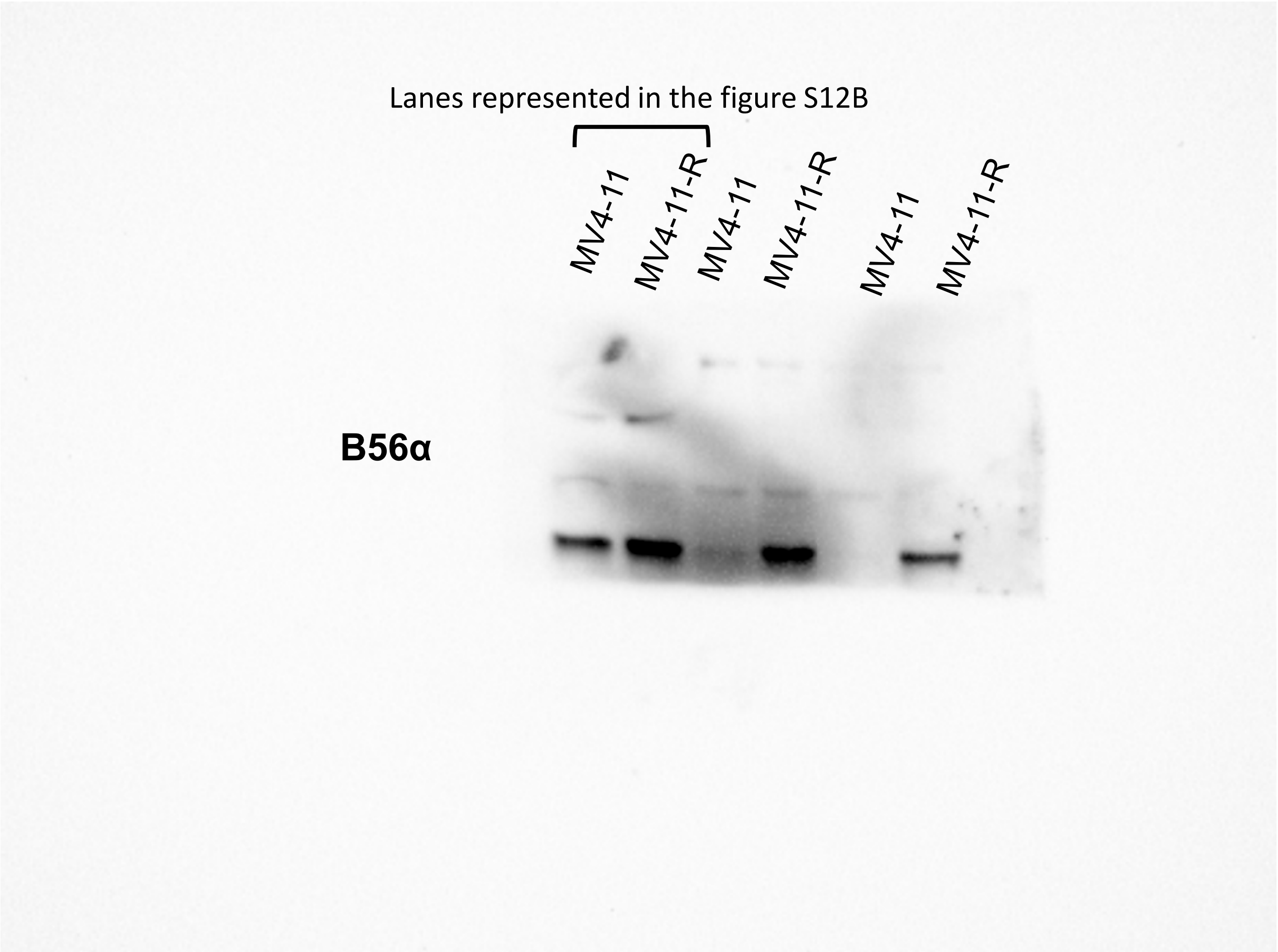

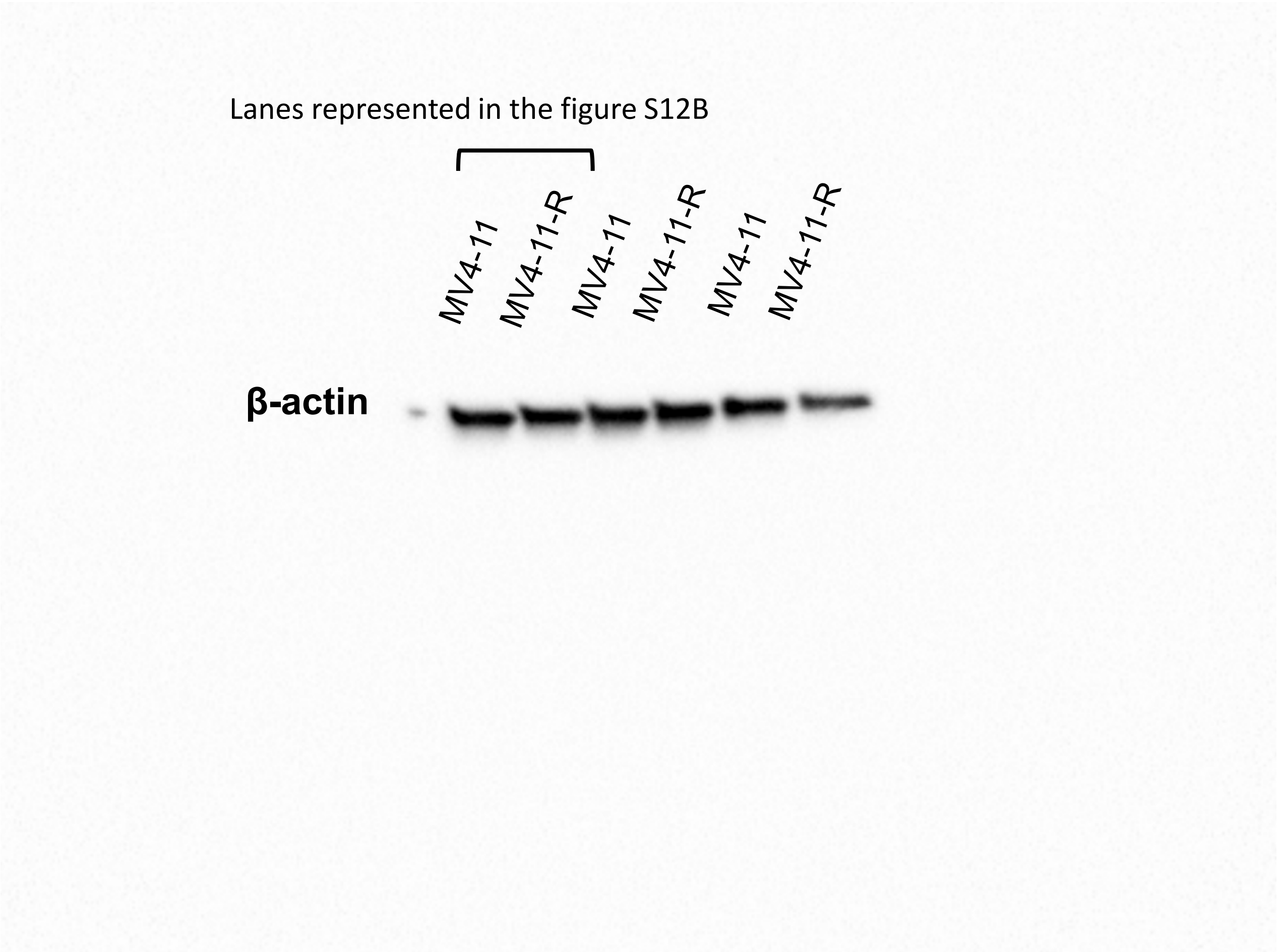

Supplement: Supplementary file 2 [file jciinsight-11-205218-s155.pdf]
